# Multigenerational Influences of the *Fut2* Gene on the Dynamics of the Gut Microbiota in Mice

**DOI:** 10.3389/fmicb.2017.00991

**Published:** 2017-06-08

**Authors:** Philipp Rausch, Sven Künzel, Abdulhadi Suwandi, Guntram A. Grassl, Philip Rosenstiel, John F. Baines

**Affiliations:** ^1^Group Evolutionary Genomics, Max Planck Institute for Evolutionary BiologyPlön, Germany; ^2^Institute for Experimental Medicine, Christian-Albrechts-Universität zu KielKiel, Germany; ^3^Department of Evolutionary Genetics, Max Planck Institute for Evolutionary BiologyPlön, Germany; ^4^German Center for Infection Research, Hannover-Braunschweig SiteHannover, Germany; ^5^Institute for Medical Microbiology and Hospital Epidemiology, Hannover Medical SchoolHannover, Germany; ^6^Institute of Clinical Molecular Biology, Christian-Albrechts-Universität zu KielKiel, Germany

**Keywords:** *Fut2*, microbiota, network analysis, community robustness, transgenerational effects, legacy effects, microbial community succession

## Abstract

The *FUT2* gene encodes an α-1,2-fucosyltransferase responsible for the expression of ABO histo-blood-group antigens on mucosal surfaces and bodily secretions. Individuals who carry at least one functional allele are known as “*secretors,*” whereas those homozygous for loss-of-function mutations are known as “*non-secretors.*” *Non-secretor* individuals are more susceptible to chronic inflammatory disorders such as Crohn’s Disease, which may be mediated by alterations in the microbiota. Here, we investigated the dynamics of microbial community assembly with respect to genotype using a *Fut2*-deficient mouse model, taking the genotype of the maternal lineage over two generations into account. We found strong differences in community assembly of microbial communities over time, depending on the *Fut2* genotype of the host and that of their progenitors. By applying network analyses, we further identified patterns of specialization and stabilization over time, which are influenced by the host and parental genotype during the process of community development. We also show genotype- and breeding-dependent patterns of community susceptibility to disturbance in a novel *in silico* approach integrating ecological- and network analysis. Our results indicate that it may be important to investigate the influence of *Fut2* genotype in a familial context in order to fully understand its role in the etiology of chronic inflammatory disorders.

## Introduction

The host-associated microbiota represent a complex phenotype composed of diverse microbial taxa and functions with important contributions to host fitness. Bacteria provide basic functions to the host such as the absorption, breakdown, and generation of nutrients (Walker et al., 2005; Tasse et al., 2010), immune regulation (Hill et al., 2012), pathogen resistance (Endt et al., 2010), and developmental cues (Hooper, 2004). Changes in community composition are linked to adverse health effects such as obesity and inflammatory bowel disease (Rausch et al., 2011), making it a target for treatment and prevention. How the host’s genetic makeup and lifestyle influence bacterial assemblages and the functions they provide is a subject of intensive research(Spor et al., 2011; Goodrich et al., 2014; Falony et al., 2016; Wang et al., 2016). Genes determining the glycan composition of mucosal surfaces are an important example of the influence of host genetic variation on microbial communities (Rausch et al., 2011; Staubach et al., 2012). The well-known α-1,2-fucosyltransferase encoded by the *FUT2* gene is responsible for the presence of ABH blood-group antigens in bodily secretions and displays widespread variation among human populations (Liu et al., 1998; Koda et al., 2000; Pang et al., 2001), characterized by loss-of-function mutations that disrupt glycan fucosylation (*i.e.*, the “*non-secretor*” phenotype) (Koda et al., 2000). These sugar chains represent an important source of nutrients and attachment sites for resident bacteria (Hooper and Gordon, 2001), but also represent a target for pathogens (Thom et al., 1989; Ilver et al., 1998; Lindesmith et al., 2003; Ruiz-Palacios et al., 2003). Furthermore, *non-secretor* status is associated with increased susceptibility to chronic inflammatory disorders such as Crohn disease (McGovern et al., 2010), possibly due to changes in the intestinal microbiota (Rausch et al., 2011; Wacklin et al., 2011, 2014; Tong et al., 2014).

Importantly, fucosylated glycans also have the potential to influence parent-offspring dynamics of microbial communities through the mucosal surface of the vaginal tract and glycosylated components of breast milk, which are some of the first sources of bacteria during and after birth (Thom et al., 1989; Newburg et al., 1990; Ruiz-Palacios et al., 2003). These first colonization steps influence long-term composition and functionality with potential health effects (Dominguez-Bello et al., 2010; Trosvik et al., 2010). Thus, it is important to understand the potential interactions between parental and offspring *FUT2* genotype. Furthermore, although fucosylated glycans serve as an important endogenous nutrient for the intestinal microbiota, particularly during periods of stress (Pickard et al., 2014), little is known about the consequences of their presence/absence on the interactions between species within a community. Thus, the main aims of this study are to (i) explore both the direct- and transgenerational effect of the *Fut2* gene on the developing murine microbial community and (ii) investigate the influence of *Fut2* on species interactions and community resistance by applying community network analyses and introducing a heuristic test *in silico*.

## Materials and Methods

### Animal Husbandry

We used the B6.129X1-*Fut2^tm1Sdo^*/J mouse model (Domino et al., 2001), kept in independently ventilated cages under specific pathogen free conditions and with *ad libitum* water and chow. The initial B6.129X1-*Fut2^tm1Sdo^*/J (*Fut2^-/-^*) mice were purchased from the Jackson Laboratory and interbred with wildtype C57BL/6J (*Fut2^+/+^*) mice. Animals were intercrossed by mating a *Fut2^-/-^* male or female (which we distinguish by “breeding direction”) with a respective wildtype mouse. The resulting heterozygous offspring were mated to obtain the experimental animals of different *Fut2* genotypes and the offspring kept together according to gender. Feces were sampled first maximally 5 days after weaning (4 weeks after birth), and then every 7 days for 11 weeks and dissected after the final sampling (see Supplementary Table S1 for an overview of mice and respective breeding and sample information; TP-time points). Fecal samples were collected on ice and immediately transferred to -80°C for storage. To obtain the mucosa associated microbial communities after dissection (aged 11 weeks after weaning, “TP11”) the tissue was gently washed in 4 ml RNAlater^®^ (Ambion^®^; Carlsbad, CA, United States) and stored separately from the luminal content in a fresh tube containing 1.5 ml RNAlater^®^. Samples preserved in RNAlater^®^ were left over night at 4°C, were spun down, and depleted of supernatant before storage at -80°C. To avoid cross-contamination, instruments were rinsed and cleaned with 70% ethanol between anatomic sites. The approval for mouse husbandry was obtained from the local veterinary office. All animals were kept under a 12 h light-dark cycle and *ad libitum* water and standard chow diet. All experiments were performed following the German Regulations of Animal Welfare as approved by the “Ministerium für Landwirtschaft, Umwelt und ländliche Räume” (Kiel, Germany).

### Histochemistry

Tissue preserved in RNAlater^®^ was washed twice in PBS, fixed in 10% formalin for 24 h and embedded in paraffin. Five micrometer sections were deparaffinized and subjected to antigen retrieval in citrate buffer. Sections were stained with rhodamine-labeled *Ulex europaeus* agglutinin-1 (UEA-1; 1:100, Vector Laboratories, Burlingame, CA, United States) for 1h at room temperature, washed and mounted in Prolong Gold containing DAPI (Molecular Probes) and investigated at 100 × magnification.

### DNA Extraction and 16S rRNA Gene Pyrosequencing

DNA was extracted from feces and RNAlater^®^-preserved mucosa samples (stored at -80°C) with the Qiagen Stool DNA Isolation Kit (Qiagen, Hilden, Germany) according to the manufacturer’s instructions. The 16S rRNA gene was amplified using forward (5′-**CTATGCGCCTTGCCAGCCCGCTCAG***TC*AGAGTTTGATCCTGGCTCAG-3′) and reverse (5′-**CGTATCGCCTCCCTCGCGCCATCAG**XXXXXXXXXX*CA*TGCTGCCTCCCGTAGGA*GT*-3′) primers flanking the V1 and V2 hypervariable regions (27F-338R) and was sequenced following the methods described in Rausch et al. (2011). The 454 Life Sciences primer B (forward) and A (reverse) adapter sequences are denoted in boldface, and the underlined sequences represent the broadly conserved bacterial primers 27F and 338R. A 2-base linker sequence (TC/CA; shown in italics) was added as recommended by Roche^®^ (454^®^) (Margulies et al., 2005). A unique 10-base multiplex identifier (designated as XXXXXXXXXX) was added to the reverse primer to tag each PCR product. Raw sequence information including metadata can be accessed at the European Bioinformatics Institute ^1^ under the accession number PRJEB13483.

### Sequence Processing and Quality Control

Raw sequences were trimmed using mothur 1.31.2 (Schloss et al., 2009) requiring no ambiguous bases, a mean quality score of ≥ 35 and a minimum length of 200 nucleotides for the V1-V2 region. Chimeric sequences were detected by Usearch 4.25 (overlap of *de novo* and database-driven detection) (Edgar, 2010) and removed. Sequences were subsampled to 1500 sequences per sample, classified via RDP classifier (RDP 9, as provided by P. Schloss) with ≥ 60% bootstrap threshold (Cole et al., 2003; Wang et al., 2007) and aligned to the SILVA core database. Operational Taxonomic Units (OTUs) were binned in mothur using the average distance clustering. Phylogenetic tree construction on representative OTU sequences was carried out using FastTree 2.1 with a gamma corrected CAT substitution model (Price et al., 2010). With an average sequence count of 1494.45 ± 36.78 SD per sample, we achieved a high species coverage of 88.49 ± 6.15% over all samples, for fecal samples 87.70 ± 4.02% at TP1, 83.81 ± 7.32% at TP3, 86.13 ± 6.96% at TP5, and 84.26 ± 4.26% at TP11, respectively, where “TP#” stands for the number of weeks after weaning. Mucosa associated microbial communities were covered at the species level on average by 87.31 ± 3.81% for cecal tissue, 92.17 ± 3.13% for colonic tissue, 94.11 ± 2.45% for the jejunum, and 94.98 ± 2.39 for ileal tissue.

### Statistical Analysis

Species diversity indices (species richness, Shannon–Weaver index, as well as the phylogenetic diversities) were calculated in R 3.0.1 using the *vegan* and *picante* packages (Kembel et al., 2010; Oksanen et al., 2011; R Core Team, 2016). Phylogenetic measures were derived using species occurrences and their respective relative phylogenetic relatedness, resulting in unweighted NRI (Net Relatedness Index) and NTI (Nearest Taxon Index) (Webb, 2000). These measures represent phylogenetic effect sizes inferred by contrasting the observed relatedness patterns in a respective sample and a null model, by maintaining species occurrence frequency and sample species richness over 999 iterations (Gotelli, 2000). The phylogenetic measures of beta diversity, unweighted UniFrac, were calculated in *mothur* and provide insight into differentially present or abundant phylogenetic lineages between communities (Lozupone and Knight, 2005). Beta diversity metrics based on shared OTU presence (Jaccard distance), or shared abundance (Bray–Curtis distance) were calculated in the *vegan* package for R. For constrained ordination (Redundancy Analysis, RDA) the OTU tables were Hellinger-transformed (Rao, 1964; Legendre and Gallagher, 2001). We further employed distance-based Redundancy Analysis based on Jaccard-, UniFrac-, and Bray–Curtis distances (Legendre and Anderson, 1999). The significance of factors and axes was assessed via a permutative ANOVA approach (5000 permutations, stratified by time point or gastrointestinal (GIT) location in global analyses). All distances were square root-transformed to avoid negative eigenvalues. The analyses of community variability were performed using the *betadisper* function with a correction for subset size, as implemented in *vegan* (Anderson, 2006). Univariate analyses of repeated measurements (*i.e.*, time course, whole GIT) was conducted using linear mixed models (LMM) with mouse ID as a random variable and a cage-dependent variance structure, and optimized by model selection using *cAIC* and its weights (Bartoń, 2016) (for example code see Supplementary Methods). For the analyses within a certain time point or GIT location, cage was identified as an important random variable for LMM analysis. All models were reduced under normal maximum likelihood (ML) and finally refitted using REML (Zuur et al., 2009; Pinheiro et al., 2011) (for example code see Supplementary Methods). Indicator species analysis was based on 10000 permutations using the generalized indicator value (*IndVal.g*) to assess the predictive value of a taxon for each respective host phenotype/category (occurrence in at least 10% of samples) (De Cáceres et al., 2010). *P*-values of the genus and OTU associations were deemed significant below a *q*-value of 0.05 (Storey et al., 2015).

Consensus genera and species-level OTU networks were generated using the SparCC algorithm (100 iterations, 10000 permutations, without singleton OTUs) implemented in *mothur* to avoid spurious correlations induced by compositionality (Friedman and Alm, 2012). The networks were constructed from the correlation matrices and weighted by the correlation coefficient in *igraph* 1.0.1 for R using only associations with *P* ≤ 0.005 (genera) or *P* ≤ 0.001 (species level OTUs) (Csardi and Nepusz, 2006). Calculation of relative centrality scores and network manipulation were also carried out in *igraph*, and weights were transformed to real values for the derivation of centralities (Freeman, 1979), modularity (Clauset et al., 2004; Newman and Girvan, 2004) and other network characteristics. Network robustness tests were performed by random attacks on networks by sequentially removing 25% of network nodes randomly over 1000 iterations, and mean values of the network characteristics for each fraction were used for further analysis. Targeted attacks were performed by sequentially removing the randomized 25% highest connected bacteria (highest node degree/highest number of connections) from the networks. We further simulated network attacks based on the association strength of bacteria to a host characteristic (*Fut2* genotype, breeding direction), as measured by *IndVal.g* (generalized indicator value) (De Cáceres et al., 2010). The top 25% of associated taxa were used for sequential removal and permuted 1000 times to exclude any effect of removal order. We identified the most influential bacteria on network stability by the average real values of relative change in network characteristics after removal (diameter, number of network clusters, size of network clusters, transitivity, closeness). Random networks of similar sizes were repeatedly simulated (100 iterations) based on a similar degree distribution as the empirical network (Viger and Latapy, 2005) by randomly and evenly distributing associations between nodes (Erdős and Rényi, 1959) and by partially rewiring random networks (*k* = 0.6, 0.8; *i.e.*, small world network with *k* as rewiring probability). The scale-free networks of similar size were constructed with a power law degree distribution (*p* = 1, 2, 4) (Goh et al., 2001), as were the exponential networks (*p* = 4, 6, 8) (Barabási and Albert, 1999). These networks were subjected to random and targeted attack as described above (100 iterations in each constructed network) and the resulting decay distribution was compared to original networks with the two-sided Kolmogorov–Smirnov test.

## Results

Throughout the study, analyses performed with respect to genotype status are noted accordingly: *Fut2^+/+^*, *Fut2^+/-^*, *Fut2^-/-^*; which account for differences in gene dose. Analyses performed with respect to the presence/absence of *Fut2* glycans are indicated as “*secretor*” (*Fut2^+/+^* and *Fut2^+/-^*) versus “*non-secretor*” (*Fut2^-/-^*). The fucosylation phenotypes of the mice were verified as previously described by Goto et al. (2014), with a common UEA-1 lectin binding pattern among Paneth cells in the small intestine irrespective of genotype and a strong Goblet cell signal in *secretor* individuals in the large intestine, which is absent in *non-secretor* individuals (see Supplementary Figure S1; Goto et al., 2014). Analyses considering breeding direction (*Fut2^+/+^* grand dam or *Fut2^-/-^* grand dam) are based on whether animals were bred by mating a *Fut2^-/-^* male or female with a respective wildtype C57BL/6J (*Fut2^+/+^*) mouse to obtain the heterozygotes from which the experimental animals originate (see Supplementary Table S1 for a sample overview).

### Phylum and Indicator Analyses Reveal Direct and Indirect Genotype Associations at Different Taxonomic Levels

**FIGURE 1:**
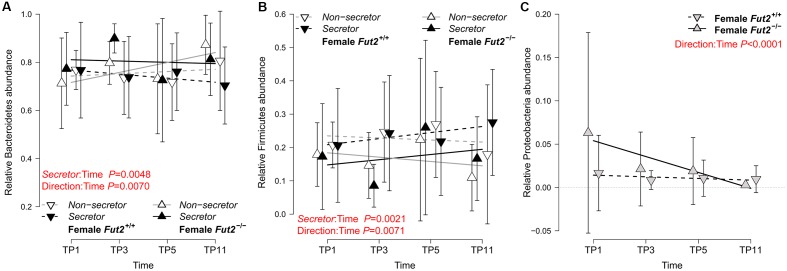
Analysis of the major bacterial phyla over the fecal sampling time points incorporating *Fut2* genotype/*secretor* status and the mouse breeding lineage (founded by *Fut2* *^-/-^* or *Fut2^+/+^* grand dam). Bacteroidetes **(A)**, Firmicutes **(B)**, and Proteobacteria **(C)** abundances over time are displayed with respect to *secretor* status and breeding direction. Phylum abundances significantly change over time, mainly dependent on breeding direction and to a smaller extend on *secretor* status (mean ± SD; see **Table 1** for detailed results). Analyses of single time points and mucosal communities are included in **Table 1** and Supplementary Table S2, respectively.

To explore the microbial communities at a basic level, we first investigated the dynamics of the major microbial taxonomic groups over time (feces) and among locations of the gastrointestinal tract (GIT; intestinal tissue sampled in mature mice, approx. 15 weeks of age/11 weeks after weaning). These analyses, based on LMMs considering cage effects, reveal interesting patterns among the major phyla Bacteroidetes, Firmicutes, and Proteobacteria with respect to *secretor* status and initial breeding direction (see **Figure 1** and **Table 1**). Firmicutes and Bacteroidetes develop significant differences over time between *secretor* types depending on breeding direction, specifically toward the last time point. Proteobacteria appear to be mainly influenced by the initial breeding direction and decrease over time in mice with a *Fut2^-/-^* grand dam, resulting in a significant difference toward the end of the time course (see **Figure 1C** and **Table 1**).

**Table 1:** Linear mixed model results of phylum abundances of the three most abundant phyla across the whole fecal time course and for each sampling time point individually.

Time points	Phylum	Factor	*DF*	*F*-value	*P*-value	marg. *R*^2^
TP1-TP11	Bacteroidetes^∗^	*Intercept*	1,99	1770.732	<0.0001	0.053
		*Secretor*	1,31	0.077	0.7829	
		Timepoint^∗∗^	1,99	2.159	0.1449	
		Direction	1,31	3.725	0.0628	
		*Secretor* × Time point	1,99	8.326	0.0048	
		Time point × Direction	1,99	7.573	0.0070	
	Firmicutes^#^	*Intercept*	1,99	2257.109	<0.0001	0.153
		*Secretor*	1,31	0.061	0.8066	
		Time point	1,99	1.772	0.1862	
		Direction	1,31	11.468	0.0019	
		*Secretor* × Time point	1,99	9.937	0.0021	
		Time point × Direction	1,99	7.548	0.0071	
	Proteobacteria^†^	*Intercept*	1,100	235.528	<0.0001	0.243
		Direction	1,32	0.650	0.4260	
		Time point	1,100	4.739	0.0318	
		Direction × Time point	1,100	24.502	<0.0001	
TP1	Firmicutes^†^	*Intercept*	1,24	128.956	<0.0001	
TP3	Firmicutes^†^	*Intercept*	1,24	148.341	<0.0001	
		Direction	1,8	5.020	0.0554	
TP5	Firmicutes^†^	*Intercept*	1,24	156.233	<0.0001	
TP11	Firmicutes^†^	*Intercept*	1,23	66.117	<0.0001	
		*Secretor*	1,23	4.993	0.0355	
TP1	Bacteroidetes^∗^	*Intercept*	1,24	199.426	<0.0001	
TP3	Bacteroidetes	*Intercept*	1,24	900.370	<0.0001	
		Direction	1,8	3.328	0.1056	
TP5	Bacteroidetes^∗^	*Intercept*	1,24	163.012	<0.0001	
TP11	Bacteroidetes^∗∗∗^	*Intercept*	1,23	48.595	<0.0001	
		*Secretor*	1,23	6.823	0.0156	
TP1	Proteobacteria^†^	*Intercept*	1,24	57.255	<0.0001	
TP3	Proteobacteria^†^	*Intercept*	1,24	89.235	<0.0001	
TP5	Proteobacteria^†^	*Intercept*	1,24	57.559	<0.0001	
TP11	Proteobacteria^†^	*Intercept*	1,24	115.108	<0.0001	
		Direction	1,8	4.587	0.0646	

*^∗^X^*2*^ transformed; ^#^X^*1/4*^ transformed; ^†^log(X+1) transformed; ^†^X^*1/2*^ transformed; ^∗∗∗^X^*3*^ transformed; ^∗∗^Time point- time coded as order of observation (0–3)*.

Phylum abundances within the mucosal communities show a weak decrease in Firmicutes and increase in Bacteroidetes in the ileal mucosa of *Fut2^+/-^* mice. Proteobacteria abundances in the ileal mucosa on the other hand display an interaction between *secretor* type and breeding direction, with a higher abundance in *secretors* of the *secretor*-founded mouse line compared to *non-secretors*, and the opposite trend in the *non-secretor*-founded mouse line. Proteobacteria are the only bacterial group in the jejunum which show an effect of *Fut2* genotype, particularly an increase in abundance with the number of functioning *Fut2* alleles. In the proximal regions of the GIT (cecum, colon), the effects of the *Fut2* genotype/*secretor* status on bacterial phyla decreases. Only Proteobacteria in the cecum are influenced by *Fut2* genotype, with a lower abundance of Proteobacteria in *non-secretor* animals (Supplementary Table S2). The major phylum in the cecum, Bacteroidetes, is less abundant in the mucosal community of animals inoculated by *Fut2^-/-^* grand dams (Supplementary Table S2).

Next, we further investigated the microbial communities for species and genera that are characteristic of *Fut2* genotype, *secretor* status, or breeding direction via indicator species analysis (De Cáceres et al., 2010). The majority of significant associations is present with regard to breeding direction and include taxa such as *Prevotella*, *Parabacteroides*, *Lactobacillus,* and *Lachnobacterium* (**Table 2** and Supplementary Tables S3, S4 and Figures S2, S3). However, several significant associations are also present for *Fut2* genotype and/or *secretor* status, including *Odoribacter* and *Propionibacterium* (*Fut2*^+/+^) and *Prevotella*, *Lachnobacterium* and *Ruminococcus* (*non-secretors*) (**Table 2** and Supplementary Table S4).

**Table 2:** Significant indicator genera for *Fut2* genotype, *secretor* status, and breeding direction across the fecal time course (bold taxa have multiple associations to a similar category).

Factor	Location/Time point	RDP Classification	Association	*IndVal.g*	*P*-value	*q*-value
*Fut2*	TP1	–	–	–	–	–
	TP3	–	–	–	–	–
	TP5	–	–	–	–	–
	TP11	***Odoribacter***	*Fut2* ^+/+^	0.8117	0.0001	0.0045
	TP1-TP11	–	–	–	–	–
*Secretor*	TP1	–	–	–	–	–
Status	TP3	–	–	–	–	–
	TP5	–	–	–	–	–
	TP11	–	–	–	–	–
	TP1-TP11	*Ruminococcus*	*Non-sec*	0.4882	0.0008	0.0400
Breeding	TP1	–	–	–	–	–
Direction	TP3	*Parasutterella*	*Fut2^-/-^* dam	0.8826	0.0010	0.0450
	TP5	***Prevotella***	*Fut2^-/-^* dam	0.8834	0.0003	0.0147
	TP11	–	–	–	–	–
	TP1-TP11	***Bacteroidetes uncl.***	*Fut2^-/-^* dam	0.7712	0.0002	0.0033
		*Escherichia/Shigella*		0.4772	0.0012	0.0150
		***Prevotella***		0.7026	0.0002	0.0033
		*Prevotellaceae uncl.*		0.7805	0.0001	0.0033
		*Bacteroides*	*Fut2*^+/+^ dam	0.8236	0.0026	0.0233
		*Barnesiella*		0.6493	0.0069	0.0493
		*Lachnospiraceae uncl.*		0.7868	0.0028	0.0233
*Fut2*	Jejunum	–	–	–	–	–
	Ileum	–	–	–	–	–
	Cecum	–	–	–	–	–
	Colon	–	–	–	–	–
	All locations	***Odoribacter***	*Fut2* ^+/+^	0.6713	0.0008	0.0186
		*Propionibacterium*		0.4360	0.0023	0.0267
*Secretor*	Jejunum	–	–	–	–	–
Status	Ileum	–	–	–	–	–
	Cecum	–	–	–	–	–
	Colon	–	–	–	–	–
	All locations	–	–	–	–	–
Breeding	Jejunum	–	–	–	–	–
Direction	Ileum	*Acetanaerobacterium*	*Fut2^-/-^* dam	0.7038	0.0007	0.0325
		***Staphylococcus***		0.7338	0.0003	0.0279
	Cecum	–	–	–	–	–
	Colon	–	–	–	–	–
	All locations	*Anaerotruncus*	*Fut2^-/-^* dam	0.5484	0.0072	0.0141
		***Bacteroidetes uncl.***		0.7511	0.0057	0.0123
		*Odoribacter*		0.7373	0.0055	0.0123
		***Prevotella***		0.6645	0.0016	0.0065
		*Prevotellaceae uncl.*		0.7560	0.0001	0.0011
		*Propionibacterium*		0.4332	0.0018	0.0065
		*Pseudoflavonifractor*		0.6850	0.0031	0.0083
		*Ruminococcaceae uncl.*		0.7853	0.0003	0.0022
		***Staphylococcus***		0.5581	0.0001	0.0011
		*Syntrophococcus*		0.6748	0.0331	0.0445
		*TM7 genus incertae sedis*		0.5239	0.0006	0.0032
		*Butyrivibrio*	*Fut2*^+/+^ dam	0.4478	0.0252	0.0387
		*Clostridium IV*		0.4482	0.0129	0.0213
		*Dorea*		0.5104	0.0330	0.0445
		*Firmicutes uncl.*		0.8957	0.0026	0.0080
		*Lachnobacterium*		0.4249	0.0128	0.0213

### Development of Phylogenetic and Species Diversity Is Dependent on Immediate and Transgenerational Genotype Effects

**FIGURE 2:**
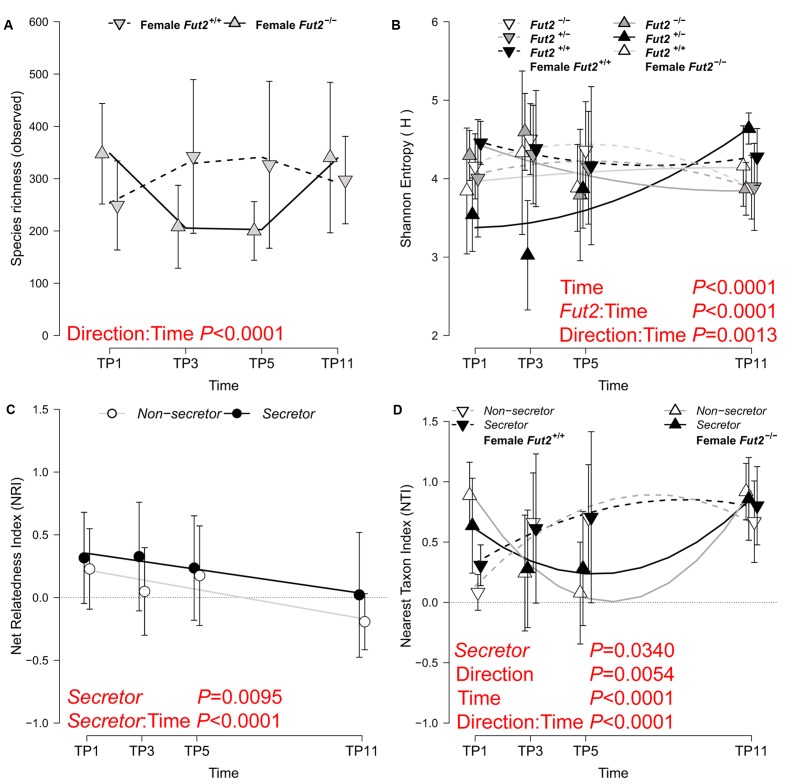
Analyses of alpha diversities based on species richness **(A)**, community complexity (**B**; Shannon Entropy), broad phylogenetic clustering (**C**; NRI), and terminal phylogenetic clustering (**D**; NTI) of fecal communities over time. The best statistical model for each metric is plotted, whereby significantly different diversity trajectories according to breeding direction **(A,B,D)**, *secretor* status **(C)**, or their interaction **(B,D)** are shown (see **Table 3** for model results). The mean ± SD of the alpha diversity values within the respective categories are plotted together with lines representing fitted linear- **(C)** or the second order polynomial fit of the models (**A,B,D**; see **Table 3** for detailed results). Model terms highlighted in red signify the most influential factors for the respective alpha diversity metric.

Community complexity is reflected by the diversity of taxa within samples (*i.e.*, alpha diversity) and is an important factor to understand ecological resilience and productivity. We measured the complexity of our community in complementary ways by describing the number of observed species (species richness), their distribution (Shannon entropy), and their relative phylogenetic relatedness (Net Relatedness Index- NRI, Nearest Taxon Index- NTI) (Webb, 2000). We identified an influence of maternal transmission on fecal microbial communities by all three alpha diversity measures considering potential cage effects, showing comparable starting diversities, strong deviations at subsequent time points and a final convergence of diversity at the end of the time course (**Figure 2** and **Table 3**). Species richness decreases after weaning in animals whose grand dam was a *non-secretor*/*Fut2^-/-^*, but equilibrates between breeding lines toward the end of the time course (TP11; Supplementary Figure S4 and Table S5). The orderedness of the species abundance distributions, as described by Shannon entropy, shows different trajectories over time according to breeding direction and *Fut2* genotype. This results in significant differences in diversity among *Fut2* genotypes at the last time point (*Fut2^+/+^* > *Fut2^+/-^* > *Fut2^-/-^*; Supplementary Figure S4 and Table S5) and evident differences of *Fut2^+/+^* mice between breeding directions.

**Table 3:** Linear mixed model results of alpha diversity across all fecal sampling time points based on species richness (observed number of species), abundance distribution (Shannon H), and phylogenetic community structure (NRI/NTI).

Alpha diversity	Model factors	*DF*	*F*-value	*P*-value	marg. *R^2^*
Species richness	*Intercept*	1,98	509.246	<0.0001	0.180
Observed	Direction	1,32	1.798	0.1894	
	Time point (poly) ^∗^^#^	2,98	0.862	0.4257	
	Direction × Time point (poly)	2,98	24.137	<0.0001	
Shannon entropy	*Intercept*	1,94	813.694	<0.0001	0.146
(X^2^ transformed)	*Fut2*	2,30	0.049	0.9522	
	Time span (poly) ^†^	2,94	21.978	<0.0001	
	Direction	1,30	1.748	0.1961	
	*Fut2* × Time span (poly)	4,94	34.422	<0.0001	
	Time span (poly) × Direction	2,94	7.169	0.0013	
Net relatedness	*Intercept*	1,101	25.602	<0.0001	0.097
Index (NRI)	*Secretor*	1,32	7.615	0.0095	
	Time span	1,101	22.348	<0.0001	
Nearest taxon	*Intercept*	1,98	568.972	<0.0001	0.760
Index (NTI)	*Secretor*	1,31	4.919	0.0340	
	Direction	1,31	8.963	0.0054	
	Time span (poly) ^†^	2,98	29.053	<0.0001	
	Direction × Time span (poly)	2,98	30.749	<0.0001	

*^∗^Time point- time coded as order of observation (0–3); ^†^Time span coded as time in weeks (0, 2, 4, 10); ^#^fitted as second order polynomial*.

With respect to the phylogenetic structure of the bacterial communities, we found stronger phylogenetic clustering in *secretor*- than in *non-secretor* mice. This phylogenetic clustering deteriorates over time until the communities become phylogenetically unstructured (*secretors*, NRI ∼ 0) to overdispersed in *non-secretors* (NRI < 0). *Secretor* status thus influences the trajectory of community assembly on a broad phylogenetic scale. The analysis of NRI shows that succession of bacterial communities results in phylogenetically less restricted communities (phylogenetic dispersion), specifically in *non-secretors*. The phylogenetic relatedness among closely related species (NTI), however, is increased in mice derived from a *Fut2^+/+^* grand dam, which indicates a phylogenetically restricted set of bacteria. These communities cluster quickly and remain clustered, while the bacterial communities derived from a *Fut2^-/-^* grand dam diversify (decrease of NTI) and become more restricted (increase of NTI) in later time points. The microbial communities passed from either breeding line converge to the end of the time course, but take different trajectories during this development. However, analyzing the diversification of phylogenetic groups over time (LMM for NRI: time span- *F*_1,101_ = 21.018, *P* < 0.0001, *R*^2^_marginal_ = 0.067; Spearman correlation: ρ = -0.3298, *P* = 8.80 × 10^-5^) indicates colonization and the establishment of more distantly related groups (*e.g*., classes, orders). The corresponding increase of NTI over time (LMM for NTI: time points- *F*_1,101_ = 23.153, *P* < 0.0001, *R*^2^_marginal_ = 0.250; Spearman correlation: ρ = 0.2967, *P* = 0.0005), however, indicates a decrease in phylogenetic distance among species and genera within a respective host. Mucosa-associated microbial communities at different locations along the GIT mostly do not differ according to breeding direction or genotype. The breeding lineage founded by *Fut2^+/+^* grand dams shows a significant decrease in species richness and entropy only in the ileum (Supplementary Table S6 and Figure S5).

In summary, community complexity significantly changes with respect to time point after weaning and host characteristics. This implies that the succession of microbial communities take different trajectories depending on the initial direction of the experimental cross, but is also influenced by *Fut2* genotype within the respective breeding lineages.

### Microbial Community Composition Differs According to Breeding Direction and *Fut2* Genotype

**FIGURE 3:**
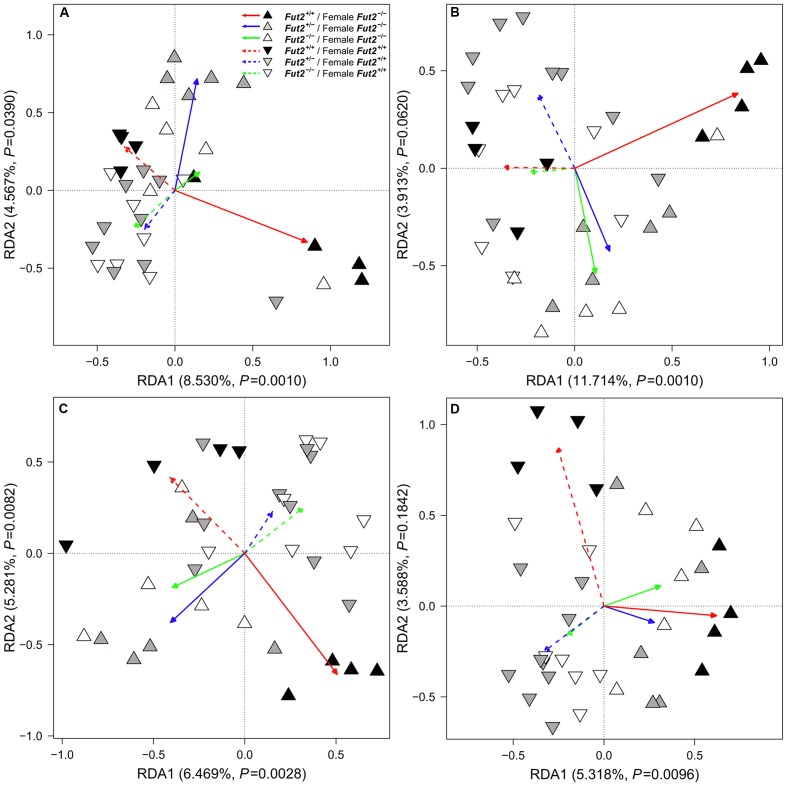
Redundancy analysis of microbial communities for each individual fecal time point **(A)** TP1, **(B)** TP3, **(C)** TP5, **(D)** TP11 according to *Fut2* genotype, breeding direction, and their interactions (arrows, see also **Table 4** for statistical model results). The main influential factor is the interaction of genotype and breeding direction.

To investigate differences between microbial communities we employed beta diversity measures based on differential species occurrence, abundance, and phylogenetic relatedness among samples (Jaccard, Bray–Curtis, and UniFrac, respectively). Interestingly, formal tests of community differentiation according to *Fut2* genotype or *secretor* status revealed no significant differences between communities in the overall dataset [Redundancy Analysis (Rao, 1964), distance-based Redundancy Analysis (Legendre and Anderson, 1999)]. However, breeding direction consistently influences microbial communities in feces and the mucosa. Further, when we consider the interaction of *Fut2* genotype and breeding direction we find mainly differences according to differential abundance in the fecal bacterial communities 1-, 3-, and 5-weeks after weaning (**Figure 3** and Supplementary Tables S7, S8). The effect of this interaction decreases toward the end of the time course, while breeding direction remains significant (Supplementary Tables S7, S8). This differentiation, specifically between breeding directions is correlated with time (Bray–Curtis: direction- *F*_1,132_ = 3.0753, *P* = 0.0002, time span- *F*_1,132_ = 3.9970, *P* = 0.0002, direction × time span- *F*_1,132_ = 1.4997, *P* = 0.0144, *adj. R*^2^ = 0.0396; Jaccard: direction- *F*_1,132_ = 2.1468, *P* = 0.0002, time span- *F*_1,132_ = 1.8771, *P* = 0.0002, direction × time span- *F*_1,132_ = 1.2623, *P* = 0.0046, *adj. R*^2^ = 0.0167). However, the analysis of all time points together (permutations stratified by time points) reveals significant community differentiation with respect to *Fut2* genotype within and between breeding directions (**Table 4** and Supplementary Figure S6). Analyses of the mucosal microbial communities of single sites in the GIT reveals mainly an effect of breeding direction on the mucosal microbial communities (Supplementary Table S9), while in the cecal mucosa differences arise between *secretor* and *non-secretor* individuals (Supplementary Table S10). A combined analysis of all mucosal microbial communities reveals genotype effects in addition to interactions with breeding direction, further emphasizing the direct and indirect influence of ABH antigens in the mucosa (**Table 4**).

**Table 4:** Community differentiation between *Fut2* genotype and breeding direction among all fecal sampling time points combined (distance-based Redundancy Analysis, Redundancy Analysis), based on shared abundance (Bray–Curtis), shared presence (Jaccard), phylogenetic relatedness (unweighted UniFrac) and the distribution of species (Redundancy Analysis).

Dataset	Distance	Factors	*DF*	*F*-value	*P*-value	*R*^2^	adj. *R*^2^
Fecal time points	Bray–Curtis	*Fut2*	2,130	1.0072	0.3198	0.0484	0.0118
(TP1-11)		Direction	1,130	2.1329	0.0002		
		*Fut2* × Direction	2,130	1.2335	0.0002		
	Jaccard	*Fut2*	2,130	1.0036	0.349	0.0477	0.0111
		Direction	1,130	2.0928	0.0002		
		*Fut2* × Direction	2,130	1.2071	0.0002		
	UniFrac	*Fut2*	2,130	1.0976	0.1394	0.0578	0.0216
	(Unweighted)	Direction	1,130	3.1389	0.0002		
		*Fut2* × Direction	2,130	1.3213	0.0102		
	Redundancy	*Fut2*	2,130	0.9681	0.4208	0.0757	0.0402
	Analysis	Direction	1,130	4.5829	0.0002		
	(Hellinger distance)	*Fut2* × Direction	2,130	2.066	0.0002		
Gastrointestinal	Bray–Curtis	*Fut2*	2,136	1.2161	0.0008	0.0508	0.0159
Tract		Direction	1,136	2.5043	0.0002		
(Jejunum, Ileum,		*Fut2* × Direction	2,136	1.1730	0.0010		
Cecum, Colon)	Jaccard	*Fut2*	2,136	1.1832	0.0012	0.0511	0.0162
		Direction	1,136	2.6433	0.0002		
		*Fut2* × Direction	2,136	1.1570	0.0034		
	UniFrac	*Fut2*	2,136	1.105	0.0322	0.0551	0.0204
	(Unweighted)	Direction	1,136	3.1875	0.0002		
		*Fut2* × Direction	2,136	1.2662	0.0018		
	Redundancy	*Fut2*	2,136	1.7277	0.0004	0.070	0.0358
	Analysis	Direction	1,136	4.0415	0.0002		
	(Hellinger distance)	*Fut2* × Direction	2,136	1.3690	0.0106		

*P-values are derived by permutations stratified by single time points and gastrointestinal tract locations*.

In addition to the differentiation between communities over time, we observe a decrease in overall community variability, and thus a homogenization of bacterial community composition. Differences in community variability become stronger between *Fut2* genotypes and *secretor* status over time (TP11- *secretor* status: Jaccard: *P* = 0.0344, Bray–Curtis: *P* = 0.0342, UniFrac: *P* = 0.3002; Euclidean: *P* = 0.0435). These differences occur specifically between homozygous *secretors* (*Fut2^+/+^*) and *non-secretors* (*Fut2^-/-^*; Jaccard: *P* = 0.0189; Bray–Curtis: *P* = 0.0217; UniFrac: *P* = 0.1548; Euclidean: *P* = 0.0756; Supplementary Figure S7). This implies a less variable community composition in *Fut2^-/-^* mice. Breeding direction, on the other hand has almost no observable effect on the variability of fecal communities. In summary, we observe strong community differentiation between breeding lines, with an additional separation of communities according to *Fut2* genotype, the latter of which is limited to the mucosa.

### Analyzes of Bacterial Co-occurrence Networks Reveals Processes of Community Development and Genotype Specific Differences of Community Stability

To infer potential interactions among bacterial community members we used co-occurrence networks of bacterial consensus genera and species, based on the correlation of their abundances. To construct networks for each time point, we split the abundance table accordingly and calculated measures of determination on the same set of bacteria and animals for each stratum using a correlation procedure considering compositionality (Friedman and Alm, 2012). We next measured different bacterial network characteristics (importance/centrality of network components) to approximate the structural importance of the single bacterial taxa within each respective network, including the number of interactions of each bacterium in the network (node degree), the PageRank^TM^ index (Brin and Page, 1998; Allesina and Pascual, 2009), and the well-known “betweenness”- and “closeness” centralities (Freeman, 1979). In simple terms, PageRank^TM^ will assign a high importance to genera that interact with other important genera recursively, while betweenness centrality measures the number of interactions in which the respective genus is a mediator (on the shortest path between any pair of taxa). Closeness centrality, on the other hand, measures how distant the node is from all other nodes in the network, whereby high scores indicate a small distance toward the rest of the network members.

**FIGURE 4:**
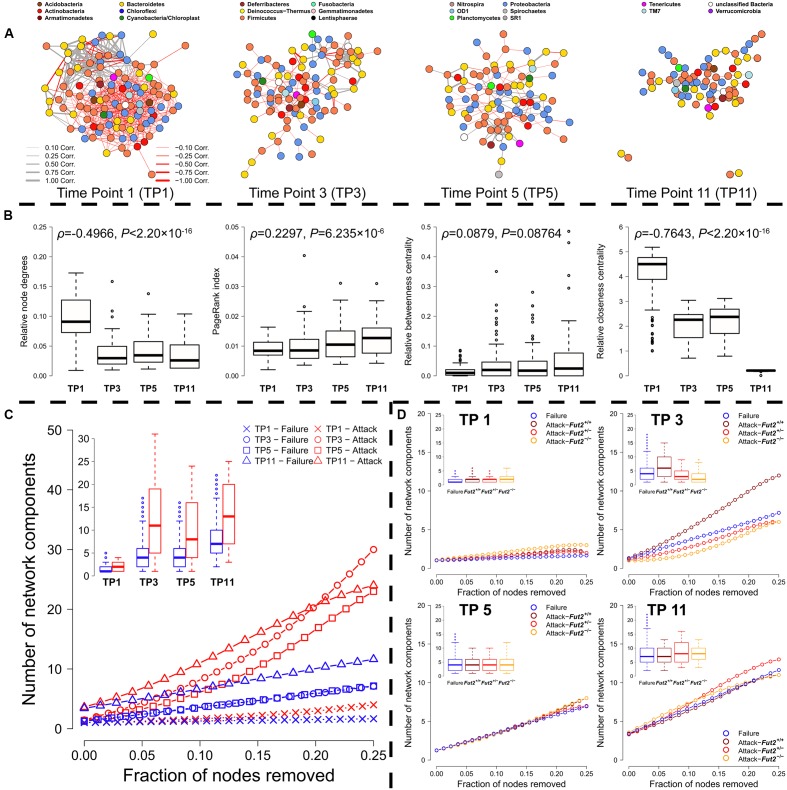
**(A)** Correlation networks of time point 1 to 11 reveal a decreasing network density (TP1 = 0.0948, TP3 = 0.0359, TP5 = 0.0402, TP11 = 0.0343) as well as decreasing centralization (TP1 = 33.196, TP3 = 18.080, TP5 = 11.801, TP11 = 11.101, Kleinberg’s hub score), but increasing diameter (TP1 = 1.381, TP3 = 2.037, TP5 = 1.974, TP11 = 3.197). **(B)** Analyses of the change of node characteristics within co-abundance networks across fecal time points including their correlation, based on the number of connections of single genera (node degree), their importance based on the quality of their connectedness (PageRank^TM^), their distance to other community members (closeness), and the importance of single bacteria as mediators within the networks (betweenness). **(C)** Analysis of network robustness based on sequential random removal (network failure-blue) and targeted removal (red) of the most integrated genera (highest number of connections/degree). The decay of the networks into smaller components is plotted as the mean decay over 1000 permutations of node removal (see Supplementary Figure S10 for additional network characteristics). **(D)** The right panels show network decay after random removal of nodes (blue) and targeted removal of either the top 25% *secretor*- (*Fut2^+/+^*- dark red, *Fut2^+/^*^-^- red) or *non-secretor* (*Fut2^-/-^*- yellow) associated genera for each time point separately (mean of 1000 iterations).

When we compare the networks between single time points, patterns resembling community succession and stabilization emerge (**Figure 4A**, for species networks see Supplememtary Figure S8A). The number of interactions between bacterial genera decreases over time (genera node degree: ρ = -0.497, *P* < 1.00 × 10^-15^; **Figure 4B**), while the species node degree increases (ρ = 0.144, *P* < 2.20 × 10^-16^; Supplementary Figure S8B). However, not only the number of connections, but also the direction of interactions changes from a higher number of negative interactions to an almost even distribution of positive and negative interactions on the genus level (positive/negative interactions; genus level: TP1: 0.292, TP3: 0.492, TP5: 0.791, TP11: 0.776; species level: TP1: 1.433, TP3: 1.170, TP5: 1.394, TP11: 1.405). Positive interactions are, however, on average stronger than negative ones (Supplementary Figure S9). Furthermore, we could identify an increase in the overall importance of single genera within these assemblages. The average mediator role of bacterial genera and species does not significantly change over time (genera betweenness: ρ = 0.0879, *P* = 0.0876, species betweenness: ρ = -0.0035, *P* = 0.8228), and the relative distance of links between community members increases with time due to reduced connectedness (ρ = –0.7643, *P* < 2.20 × 10^-16^; see **Figure 4B**), while distances among species decrease over time (ρ = 0.1692, *P* < 2.20 × 10^-16^; Supplementary Figure S8B). The PageRank^TM^ as a generalized importance index reveals an increase of importance with time in genera networks (ρ = 0.148, *P* = 2.21 × 10^-6^; **Figure 4B**), a pattern also present within species networks (ρ = 0.127, *P* = 2.31 × 10^-16^; Supplementary Figure S8B). All these results hint toward an increasing stabilization of a core set of strongly interacting bacteria over time, with increasing mutualistic- and less competitive interactions between single taxa, which increases modularity (see Supplementary Figure S10 for genus-based network modularity; OTU network modularity: TP1: 0.838, TP3: 0.936, TP5: 0.962, TP11: 0.960) (Clauset et al., 2004; Newman and Girvan, 2004).

To test the robustness of the microbial communities against different disturbance regimes, we simulated random loss of community members or targeted depletion of important keystone members (*i.e.*, network hubs- based on the number of their interactions/node degree) (Albert et al., 2000). For each respective co-occurrence network we measured the resulting changes in the network such as fragmentation (number and size of connected components), size (diameter), and clustering (transitivity, closeness). As expected for ecological communities, which are mainly classified as scale-free networks, we found high resistance against random failures, but fast deterioration of communities after removing small fractions of important, highly connected nodes (see **Figure 4C** and Supplementary Figure S11, attack *vs.* random failure). We observed the highest resistance against community collapse shortly after weaning. In this stage many weak interactions seem to prevent the community from rapid deterioration (**Figure 4** and Supplementary Figures S10, S11). In later time points bacterial interactions appear to solidify, while others are lost during succession. This differentiation increases the importance of single community members (see the increase of PageRank^TM^ above) and therefore the vulnerability of the system. Thus, the targeted removal of important mediators (targeted attack) disturbs communities much stronger than random network failures, as shown for example in the stronger network fragmentation (see **Figure 4C** and Supplementary Figure S8C) and size of the biggest connected component (Supplementary Figures S11A,B). The transitivity/clustering of the networks deteriorates much quicker under targeted removal compared to random removal throughout the time course (Supplementary Figures S11E,F), as does network diameter/size (Supplementary Figures S11C,D) and closeness centrality (increase of distance between nodes; Supplementary Figures S11G,H). In sum, these analyses reveal high community resistance in the early phases of community development and a high susceptibility of climax communities to the removal of key players.

The most influential bacteria (top 10 members for each time point) on network decay were relatively universal and also include indicator taxa for genotype, *secretor* status, and breeding direction. Several taxa appear to have common large effects on most network parameters (diameter, size of biggest cluster, transitivity, closeness) over more than a single time point, such as *Alistipes* (OTU-36), *Rikenella* (OTU-9), and *Paludibacter* (OTU-120) in species networks, or *Alistipes, Enterorhabdus*, *Lachnospiraceae*, *Parasutterella*, and *Ruminococcus* in the genus-level networks (Supplementary Tables S11, S12). Several taxa with a large influence on network decay are also indicators for host factors (globally or time point specific), such as *Ruminococcus,* which is associated to the *Fut2^-/-^* genotype/*non-secretor* status (**Table 2**). Indicators for breeding direction are also found among structurally important bacteria including *Lachnospiraceae*, *Barnesiella*, and *Bacteroides* as indicators for the *Fut2*^+/+^ grand dam, or *Prevotellaceae*, *Parasutterella*, and *Escherichia/Shigella* as indicators for the *Fut2^-/-^* grand dam (**Table 2** and Supplementary Table S11). Similarly, several of the most important OTUs in the species level networks are indicators of breeding direction such as *Paludibacter*, *Helicobacter*, *Oscillibacter*, and *Anaerophaga* (*Fut2*^+/+^ grand dam), or *Meniscus,* *Tannerella,* and *Bacteroides* as indicators for the *Fut2^-/-^* grand dam (Supplementary Tables S3, S12). These findings imply a relatively large structural role of bacteria influenced by breeding legacy and transgenerational genotype effects, but also some direct genotype effects (*e.g.*, *Ruminococcus* association). Furthermore, the behavior of the empirical networks under random error- and targeted attack regimes is similar to simulated networks of a comparable degree distribution (small world, random, degree sequence, power law), but resembles most closely the behavior of “small world”-like networks of similar size (Supplementary Figure S12 and Table S13). Interestingly, the co-occurrence network derived from the last time point appears more resilient than any of the simulated network topologies tested (Supplementary Figure S12).

To investigate the influence of the *Fut2* genotype, *secretor* status, and breeding direction on the robustness of microbial communities, we adapted the heuristics described above and sequentially removed the top 25% of genera and species associated to the respective host factors (as measured by their respective indicator values (De Cáceres et al., 2010) from the network. The removal of bacteria associated to *secretor* genotypes influences network fragmentation (*i.e.*, number of connected components) earlier and stronger than the removal of *non-secretor* associated bacteria (**Figure 4C** and Supplementary Figures S13, S14). Especially in early community development (TP1, TP3) the average number of resulting subnetworks is higher than after removal of *non-secretor* associated bacteria. *Secretor*-dependent effects on the network characteristics are most obvious in the species networks, which also show a higher influence of *secretor*-associated bacteria (**Figure 4** and Supplementary Figure S14). We observe similar patterns with respect to *Fut2* genotype, with the strongest effect on network characteristics when removing *Fut2*^+/+^-associated bacteria (Supplementary Figures S8D, S15, S16). Further, removal of bacteria associated to a *Fut2*^+/+^ grand dam disturbed the network earlier than the removal of bacteria associated to the *Fut2^-/-^* grand dam (Supplementary Figures S17, S18). However, disturbances introduced by the removal of bacteria (genera/species) associated to the *Fut2^-/-^* grand dam are on average higher than the removal of bacteria associated to the *Fut2*^+/+^ grand dam or random removal. Thus, community stability appears to significantly differ with respect to breeding direction in our experimental cohort of mice.

In summary, our results imply that changes in network characteristics over time resemble patterns of community succession, including an increase in taxon importance, attack vulnerability, and taxonomic/phylogenetic heterogeneity. Furthermore, we find that host genotype and legacy effects can significantly influence network stability. These results illustrate the importance of specialized key members in microbial communities and may suggest new strategies to evaluate community stability and dynamics.

## Discussion

Host-associated microbial communities can be viewed as a complex and plastic phenotype influenced by numerous factors including host genetics, initial community initiation (mode of delivery), community disturbances (antibiotics) and diet. Glycans represent a major part of the mucosa acting as attachment sites for the microbial community and also as essential nutrient sources (Hooper and Gordon, 2001). Furthermore genes responsible for glycan synthesis appear essential for host-bacteria homeostasis as their expression is directly triggered by the resident microbial communities (Nanthakumar et al., 2003; Meng et al., 2007). Glycan liberation through the microbial community is a highly collaborative process (Rakoff-Nahoum et al., 2016) that can influence the colonization success of commensals and pathogens (Ng et al., 2013). Liberated fucose seems to represent a danger response of intestinal epithelial cells to buffer disturbances in microbial communities during infection, through a modulation of quorum sensing- and virulence- mechanisms (Pacheco et al., 2012; Pickard et al., 2014), and thus appears to represent a direct link between the host and its resident communities.

In an effort to further assess the role of fucosylated glycans on species interactions and community resistance, we focused on taxa associated to *Fut2* genotype- and/or *secretor* status. Although the evidence provided by our study is indirect, the relevance of the associated taxa we detect is in part supported by previous studies. The single indicators detected for *secretor* genotypes, including *Odoribacter,* are known to digest fucose (Hardham et al., 2008; Nagai et al., 2010). Interestingly, the *Fut2^-/-^* associated genus *Ruminococcus* displays high variability in its glucosidase repertoire and can even lack fucosidases altogether (Crost et al., 2013). Members of the *Lachnospiraceae, Ruminococcaceae* (*e.g.*, *Ruminococcus*), and *Prevotellaceae* (*e.g.*, *Prevotella*) were also shown to differ according to *secretor* status in a large study on fecal microbial communities, although were not statistically significant after correction for multiple testing (Davenport et al., 2016). Further, although there is taxonomic overlap (*e.g.*, members of *Lachnospiraceae*, *Ruminococcaceae*) between the current and other previous studies (Kashyap et al., 2013; Tong et al., 2014), the direction of the association with respect to *Fut2* genotype differs.

Several explanations can be provided for the discrepancies between the current and other studies. On the one hand, our study is based on a native lab mouse microbiota, whereas Kashyap et al. (2013) and Tong et al. (2014) used humanized mouse models and humans in their analyses. On the other hand, conflicting results between different human studies have also been reported (Rausch et al., 2011; Davenport et al., 2016). As pointed out by Davenport et al. (2016), it is important to control for diet and disease status. However, we note that it is additionally important to consider the source material for metagenomic analysis, as previous studies of *FUT2* were based on mucosal biopsies (Rausch et al., 2011), where a greater host genotype effect may be expected (Spor et al., 2011; Linnenbrink et al., 2013), in contrast to fecal material (Davenport et al., 2016). The observations of *Fut2* genotype-dependent effects of the current study garner support from both mucosal and fecal sampling, for which the environment and breeding scheme were carefully controlled, as well as by performing longitudinal sampling and analysis.

Indeed, the temporal development of ecological communities is another important and dynamic process, which we addressed through longitudinal sampling. Accordingly, we identified a pattern of succession among *non-secretors* that changes from a phylogenetically clustered community (co-occurring bacteria closer related than expected by chance) to a pattern of phylogenetic overdispersion (co-occurring bacteria more distantly related than expected by chance). Early in development microbial communities are phylogenetically clustered, possibly due to their common origin from *secretor* parents (*Fut2^+/-^*). Over time *secretor* communities develop into neutrally assembled communities, whereas those of *non-secretor* individuals appear to be overdispersed. This may be interpreted as a sign of competitive exclusion of moderately related bacteria with comparable metabolic dependencies within *non-secretor* communities (Zaneveld et al., 2010; Violle et al., 2011), or as a sign of mutualistic interactions between distantly related species (Valiente-Banuet and Verdú, 2007; Violle et al., 2011). The fucose provided by *secretor* mothers will eventually dissipate in *non-secretor* offspring, which increases the competition for other available glycans and may open niches for functional and phylogenetically distant bacteria. *Secretor* status mainly influences broad phylogenetic patterns over time, while breeding direction influences the number of species and co-occurrence of closely related species. NTI increases over time, whereas NRI decreases. Closely related taxa thus appear to exclude each other over time, while more distantly related taxa can co-occur through reduced niche overlap (Zaneveld et al., 2010; Violle et al., 2011). Most bacteria in the investigated communities show weak competitive interactions, which are a characteristic and stabilizing pattern in ecological communities (Mougi and Kondoh, 2012; Tang et al., 2014). The reactions of taxa negatively associated to each other are asynchronous and balance the reduction of one species by the complementary increase of other community members (Tilman, 1996). This so called “portfolio”- or “insurance” effect can decrease the influence of environmental disturbances by a release of competition among community members (Yachi and Loreau, 1999; Loreau, 2010). Host genotype in interaction with environmental factors has been shown to change community function in the context of *Fut2* variation (Kashyap et al., 2013). Microbial communities can also change *Fut2* expression, *e.g.*, through different LPS concentrations generated by community composition differences or bacterial load (Nanthakumar et al., 2003; Meng et al., 2007; Pickard et al., 2014) and thus may modify *Fut2* genotype differences, similar to factors like nutrition (Kashyap et al., 2013) and be reflected in immunological and susceptibility differences, or community stability/resilience (Goto et al., 2014; Pham et al., 2014; Rausch et al., 2015b).

Community resilience is another cornerstone of homeostasis and is suspected to play a major role in the development of dysbiosis of gut bacterial communities (Tamboli et al., 2004; Rausch et al., 2015b). We tested the resistance of communities *in silico* through random removal of single bacteria compared to targeted attacks on central community members to simulate natural community fluctuation (random removal, random failure), and the introduction of a highly competitive pathobiont, predator, or an antibiotic (targeted removal). Such strategies were previously employed to investigate the stability of complex networks like the internet, cellular- (protein interaction, metabolic networks) and ecological networks (Albert et al., 2000; Jeong et al., 2000; Memmott et al., 2004; Widder et al., 2014). Increasing modularity, as observed in our microbial co-occurrence networks, was shown to allow for a higher total abundance of community members and diversity, which implies a higher productivity of structured communities by reducing interspecific competition (Bastolla et al., 2009). Modular, differentiated networks tend to be more robust to random fluctuations, as perturbations are compartmentalized within the modular structure and do not spread fast throughout the network (Albert et al., 2000; Memmott et al., 2004). Our analyses further highlight characteristics of error tolerance and attack susceptibility in the empirical networks comparable to small world- and exponential networks (Albert et al., 2000). However, the microbial communities appear astoundingly robust against random attacks/failure, outperforming all simulated topologies, especially in the later stages of community development. By investigating the average scores obtained from the repeated random node removals we could identify several taxa which show higher than average importance on network stability with characteristics such as bile resistance/synthesis [*e.g.*, *Ruminococcus*, *Alistipes*, *Rikenella* (Bomar et al., 2011; Graf, 2014; Devlin and Fischbach, 2015)], quinone synthesis [*e.g.*, *Parasutterella* (Nagai et al., 2009)], or mucus/glycan association [*e.g.*, *Alistipes*, *Rikenella, Ruminococcus* (Bomar et al., 2011; Crost et al., 2013; Graf, 2014); see also Supplementary Tables S11, S12]. We extended the concept of network attack to specifically test the influence of specific host characteristics by removing bacteria associated to a specific *Fut2* genotype, *secretor* status, and breeding direction. These heuristics revealed a higher structural importance of bacteria associated to *secretor* genotypes, or a *non-secretor* grand dam. This difference in stability may be a product of mutual relationships between bacteria essential to liberate or use fucosylated ABH blood-group antigens from the mucosal surfaces (Rakoff-Nahoum et al., 2016), especially when we consider that several of the structurally important bacteria can be influenced by fucosylated glycans, *e.g.*, *Barnesiella* (Weiss et al., 2014), *Lactobacillus* (Uchida et al., 2006), *Odoribacter* (Hardham et al., 2008; Nagai et al., 2010), *Helicobacter* (Magalhaes et al., 2009), or *Robinsoniella* (Cotta et al., 2009; Shen et al., 2010). Robustness differences associated to genotype or breeding direction may have consequences for host fitness when considering the potential for dysbiosis after environmental disturbances. A loss of key members/functions could include the loss of, *e.g.*, glycan liberation for other bacteria (Ng et al., 2013), or a loss of colonization resistance (Eklof and Ebenman, 2006; Pham et al., 2014; Rausch et al., 2015b). Thus, depending on the underlying community network, dysbiotic changes may be buffered by, *e.g.*, weak negative interactions, or escalate into cascading community changes through a loss of central hub bacteria.

Although our analyses offer important insight into the role of a host-derived glycan in species interactions and community resilience, it should be recognized that the co-occurrence networks constructed in this study are only approximations of real dependencies, and the interactions we observe certainly contain a degree of indirect and/or false positive interactions. Thus, conclusions based on this type of data should necessarily consider these shortcomings (Weiss et al., 2016). However, addressing community dynamics using network approaches remains important, as they have the potential to critically enhance our understanding of microbial communities at the level of interactions between taxa and may ultimately help explain the origins of dysbiosis and disease susceptibility. Our approach of testing microbial communities *in silico* may thus provide interesting new perspectives for microbial community research.

## Author Contributions

PRa, PRo, and JB designed the research; PRa, AS, GG, and SK performed the experiments; PRa analyzed data; PRa and JB wrote the paper.

## Conflict of Interest Statement

The authors declare that the research was conducted in the absence of any commercial or financial relationships that could be construed as a potential conflict of interest.

## Footnotes

1 www.ebi.ac.uk
