# Supplementary material for: Multigenerational Influences of the Fut2 Gene on the Dynamics of the Gut Microbiota in Mice
Source: Front Microbiol. 2017 Jun 8;8:991. doi: 10.3389/fmicb.2017.00991 (PMC5463037; doi:10.3389/fmicb.2017.00991)
Supplement: Supplementary file 1 [file Data_Sheet_1.pdf]

Title: Multigenerational influences of the *Fut2* gene on the dynamics of the gut microbiota in mice

Authors: Philipp Rausch[1,2], Sven Künzel[3], Abdulhadi Suwandi[4,5], Guntram A. Grassl[4,5], Philip Rosenstiel[6], John F. Baines[1,2,*]

[1] Department of Evolutionary Genomics, Max Planck Institute for Evolutionary Biology, Plön, Germany

[2] Institute for Experimental Medicine, Christian-Albrechts-University of Kiel, Kiel, Germany

[3] Department of Evolutionary Genetics, Max Planck Institute for Evolutionary Biology, Plön, Germany

[4] German Center for Infection Research (DZIF), Hannover-Braunschweig Site, Carl-Neuberg-Str. 1, 30625 Hannover, Germany

[5] Institute of Medical Microbiology and Hospital Epidemiology, Hannover Medical School, Carl-Neuberg-Str. 1, 30625 Hannover, Germany

[6] Institute of Clinical Molecular Biology, Christian-Albrechts-University of Kiel, Kiel, Germany

Dr. Philipp Rausch (rausch@evolbio.mpg.de)

Dr. Sven Künzel (kuenzel@evolbio.mpg.de)

Dr. Abdulhadi Suwandi (Suwandi.Abdulhadi@mh-hannover.de)

Prof. Guntram A. Grassl (Grassl.Guntram@mh-hannover.de)

Prof. Philip Rosenstiel (p.rosenstiel@mucosa.de)

Prof. John F. Baines* (baines@evolbio.mpg.de)

Correspondence:

Prof. John F. Baines* (baines@evolbio.mpg.de)

Supplementary Methods:

Examplary R code for model selection employed for the fecal time course analysis and analysis of the whole gastrointestinal tract via linear mixed models (alpha diversity, phylum abundances).

```
library("nlme")
library("MuMIn")
###############################################################
###############################################################
###############################################################
### EXAMPLE: fecal time course (TP1-TP11), applies to analysis
### of whole gastrointestinal tract as well (exchange
### timepoint by GIT_location (jejunum-ileum-cecum-colon))
###############################################################
###############################################################
###############################################################
# Factors:
# Sex - sex of individual mouse (factor)
# Secretor_status: Secretor(Fut2+/+/Fut2+/-)/nonsecretor(Fut2-/-)
# (factor)
# Fut2_genotype: Fut2+/+/Fut2+/-/Fut2-/- (factor)
# Breeding_direction: grand dam Fut2+/+/grand dam Fut2-/-
# (factor)
# Timepoint: numeric 1/3/5/11 weeks after weaning (numeric)
# GIT_location: jejunum/ileum/cecum/colon mucosal surface
# sampled (factor)

###
###Define random/correlation part for data
###

### Baseline model without nestedness, but REML fitted
### (restricted log-likelihood maximization)

aa_null <-
gls(Diversity~Sex*Secretor_status*Breeding_direction*Timepoint,
data=data_set_diversity, method="REML",
control=lmeControl(opt="optim",maxIter=1000,msMaxIter=1000))

### nested structure in saturated model

nested_a1 <-
lme(Diversity~Sex*Secretor_status*Breeding_direction*Timepoint,
data=data_set_diversity,random=(~1|Mouse_ID),method="REML")

nested_a2 <-
lme(Diversity~Sex*Secretor_status*Breeding_direction*Timepoint,
data=data_set_diversity,random=(~1|Cage),method="REML")
```

```
nested_a3 <-
lme(Diversity~Sex*Secretor_status*Breeding_direction*Timepoint,
data=data_set_diversity,random=(~1|Breeding_cage),method="REML"
)

nested_a4 <-
lme(Diversity~Sex*Secretor_status*Breeding_direction*Timepoint,
data=data_set_diversity,random=(~Timepoint|Mouse_ID),method="RE
ML")

nested_a5 <-
lme(Diversity~Sex*Secretor_status*Breeding_direction*Timepoint,
data=data_set_diversity,random=(~Timepoint|Cage),method="REML")

nested_a6 <-
lme(Diversity~Sex*Secretor_status*Breeding_direction*Timepoint,
data=data_set_diversity,random=(~Timepoint|Breeding_cage),metho
d="REML")

### nested structure & correlation structure ("weights") in
### saturated model

nested_corr_a1 <-
lme(Diversity~Sex*Secretor_status*Breeding_direction*Timepoint,
data=data_set_diversity,random=(~1|Mouse_ID),method="REML"
,weights=varIdent(form=~1|Cage))

nested_corr_a2 <-
lme(Diversity~Sex*Secretor_status*Breeding_direction*Timepoint,
data=data_set_diversity,random=(~1|Cage),method="REML"
,weights=varIdent(form=~1|Cage))

nested_corr_a3 <-
lme(Diversity~Sex*Secretor_status*Breeding_direction*Timepoint,
data=data_set_diversity,random=(~1|Breeding_cage),method="REML"
,weights=varIdent(form=~1|Cage))

nested_corr_a4 <-
lme(Diversity~Sex*Secretor_status*Breeding_direction*Timepoint,
data=data_set_diversity,random=(~Timepoint|Mouse_ID),method="RE
ML" ,weights=varIdent(form=~1|Cage))

nested_corr_a5 <-
lme(Diversity~Sex*Secretor_status*Breeding_direction*Timepoint,
data=data_set_diversity,random=(~Timepoint|Cage),method="REML"
,weights=varIdent(form=~1|Cage))

nested_corr_a6 <-
lme(Diversity~Sex*Secretor_status*Breeding_direction*Timepoint,
data=data_set_diversity,random=(~Timepoint|Breeding_cage)
,method="REML" ,weights=varIdent(form=~1|Cage))

###
```

```
### selecting optimal nesting/correlation structure by AIC and
### Likelihood test
###

anova(
aa_null,nested_a1,nested_a2,nested_a3,nested_a4,nested_a5
,nested_a6,nested_corr_a1,nested_corr_a2,nested_corr_a3
,nested_corr_a4,nested_corr_a5,nested_corr_a6
)

###
### Model selection using best random/correlation structure
### under "LM" (log-likelihood maximization) instead of "REML"
### (best model/correlation structure (varIdent(form=~1|Cage)),
### nestedness structure ###(random=(~1|Mouse_ID)))
###

nested_corr_a1 <-
lme(Diversity~Sex*Secretor_status*Breeding_direction*Timepoint,
data=data_set_diversity,random=(~1|Mouse_ID),method="ML"
,weights=varIdent(form=~1|Cage))

selection_nested_corr_a1 <- dredge(nested_corr_a1, beta=FALSE,
evaluate=TRUE, rank="AICc",fixed=NULL, trace=TRUE)

nested_corr_a2 <-
lme(Diversity~Sex*Secretor_status*Breeding_direction*poly(Timep
oint,2),data=data_set_diversity,random=(~1|Mouse_ID)
,method="ML" ,weights=varIdent(form=~1|Cage))

selection_nested_corr_a2 <- dredge(nested_corr_a2, beta=FALSE,
evaluate=TRUE, rank="AICc",fixed=NULL, trace=TRUE)

nested_corr_a3 <-
lme(Diversity~Sex*Secretor_status*Breeding_direction*Timepoint,
data=data_set_diversity,random=(~1|Mouse_ID),method="ML"
,weights=varIdent(form=~1|Cage))

selection_nested_corr_a3 <- dredge(nested_corr_a3, beta=FALSE,
evaluate=TRUE, rank="AICc",fixed=NULL, trace=TRUE)

nested_corr_a4 <-
lme(Diversity~Sex*Secretor_status*Breeding_direction*
poly(Timepoint,2),data=data_set_diversity,random=(~1|Mouse_ID),
method="ML" ,weights=varIdent(form=~1|Cage))

selection_nested_corr_a4 <- dredge(nested_corr_a4, beta=FALSE,
evaluate=TRUE, rank="AICc",fixed=NULL, trace=TRUE)

### Fut2 genotype instead secretor status

nested_corr_geno_a1 <-
lme(Diversity~Sex*Fut2_genotype*Breeding_direction*Timepoint,
```

```
data=data_set_diversity,random=(~1|Mouse_ID),method="ML"
,weights=varIdent(form=~1|Cage))

selection_nested_corr_geno_a1 <- dredge(nested_corr_geno_a1,
beta=FALSE, evaluate=TRUE, rank="AICc",fixed=NULL, trace=TRUE)

nested_corr_geno_a2 <-
lme(Diversity~Sex*Fut2_genotype*Breeding_direction*poly(Timepoi
nt,2), data=data_set_diversity,random=(~1|Mouse_ID),method="ML"
,weights=varIdent(form=~1|Cage))

selection_nested_corr_geno_a2 <- dredge(nested_corr_geno_a2,
beta=FALSE, evaluate=TRUE, rank="AICc",fixed=NULL, trace=TRUE)

nested_corr_geno_a3 <-
lme(Diversity~Sex*Fut2_genotype*Breeding_direction*Timepoint,
data=data_set_diversity,random=(~1|Mouse_ID),method="ML"
,weights=varIdent(form=~1|Cage))

selection_nested_corr_geno_a3 <- dredge(nested_corr_geno_a3,
beta=FALSE, evaluate=TRUE, rank="AICc",fixed=NULL, trace=TRUE)

nested_corr_geno_a4 <-
lme(Diversity~Sex*Fut2_genotype*Breeding_direction*
poly(Timepoint,2),
data=data_set_diversity,random=(~1|Mouse_ID),method="ML"
,weights=varIdent(form=~1|Cage))

selection_nested_corr_geno_a4 <- dredge(nested_corr_geno_a4,
beta=FALSE, evaluate=TRUE, rank="AICc",fixed=NULL, trace=TRUE)

###
### Get top 10 models based on lowest AICc and model weights as
### defined by "dredge function"
###

S_nested_corr_a1 <- lme(Diversity~1,
data=data_set_diversity,random=(~1|Mouse_ID),method="ML"
,weights=varIdent(form=~1|Cage))

S_nested_corr_a2 <- lme(Diversity~Breeding_direction,
data=data_set_diversity,random=(~1|Mouse_ID),method="ML"
,weights=varIdent(form=~1|Cage))

S_nested_corr_a3 <-
lme(Diversity~Secretor_status+Breeding_direction,
data=data_set_diversity,random=(~1|Mouse_ID),method="ML"
,weights=varIdent(form=~1|Cage))

…

AICc(S_nested_corr_a1, S_nested_corr_a2, S_nested_corr_a3, …)
```

```
anova(S_nested_corr_a1, S_nested_corr_a2, S_nested_corr_a3, …)

### Refitting of final model under REML to obtain final
### P-Values

S_nested_corr_a2 <- lme(Diversity~Breeding_direction,
data=data_set_diversity,random=(~1|Mouse_ID),method="REML"
,weights=varIdent(form=~1|Cage))

anova(S_nested_corr_a2)

shapiro.test(resid(S_nested_corr_a2))

### has to be ###insignificant, otherwise transformation and
### redo selection
### Likelihood-ratio based pseudo-R-squared of final model
r.squaredLR(S_nested_corr_a2)
```

Examplary R code of the model selection approach used for the analysis of single time points as well as single gastrointestinal tract locations via linear mixed models (alpha diversity, phylum abundances).

```
library("nlme")
library("MuMIn")
###############################################################
###############################################################
###############################################################
### EXAMPLE: Single fecal time points, applies also to the
### analysis of single locations of the gastrointestinal tract
###############################################################
###############################################################
###############################################################

### Define random/correlation part for data
### no nested structure in saturated model, but REML fitted

### Baseline model without nestedness, but REML fitted
### (restricted log-likelihood maximization)

aa_null <-
gls(Diversity~Sex*Secretor_status*Breeding_direction,
data=data_set_diversity_TP1, method="REML")

### nested structure in saturated model

nested_a1 <-
lme(Diversity~Sex*Secretor_status*Breeding_direction,
data=data_set_diversity_TP1, random=(~1|Cage),method="REML")
```

```
nested_a2 <-
lme(Diversity~Sex*Secretor_status*Breeding_direction,
data=data_set_diversity_TP1,random=(~1|Breeding_cage)
,method="REML")

nested_a3 <-
lme(Diversity~Sex*Secretor_status*Breeding_direction,
data=data_set_diversity_TP1,random=(~1|Breeding_cage/Cage)
,method="REML")

###
### selecting nestedness structure by AIC and Likelihood test
###

anova( aa_null,nested_a1, nested_a2, nested_a3 )

###
### Model selection using best random/correlation structure
### under "LM" (log-likelihood maximization) instead of "REML"
###

nested_a1 <-
lme(Diversity~Sex*Secretor_status*Breeding_direction,
data=data_set_diversity_TP1,random=(~1|Cage),method="ML"
,weights=varIdent(form=~1|Cage))

selection_nested_a1 <- dredge(nested_ a1, beta=FALSE,
evaluate=TRUE, rank="AICc",fixed=NULL, trace=TRUE)

### Fut2 genotype instead secretor status

nested_geno_a1 <-
lme(Diversity~Sex*Fut2_genotype*Breeding_direction,
data=data_set_diversity_TP1,random=(~1|Mouse_ID),method="ML"
,weights=varIdent(form=~1|Cage))

selection_nested_geno_a1 <- dredge(nested_geno_a1, beta=FALSE,
evaluate=TRUE, rank="AICc",fixed=NULL, trace=TRUE)

###
###
### Get top 10 models based on lowest AICc and model weights as
### defined by "dredge" function
###
###

S_nested_a1 <- lme(Diversity~1,
data=data_set_diversity_TP1,random=(~1|Cage),method="ML")

S_nested_a2 <- lme(Diversity~Breeding_direction,
data=data_set_diversity_TP1,random=(~1|Cage),method="ML")
```

```
S_nested_a3 <-
lme(Diversity~Secretor_status+Breeding_direction,
data=data_set_diversity_TP1,random=(~1|Cage),method="ML")

…

AICc(S_nested_ a1, S_nested_a2, S_nested_a3, …)
anova(S_nested_a1, S_nested_a2, S_nested_a3, …)
### Refitting of final model under REML to obtain final
### P-Values

S_nested_a2 <- lme(Diversity~Breeding_direction,
data=data_set_diversity_TP1,random=(~1|Cage),method="REML")

### has to be insignificant, otherwise transformation and redo
### selection
shapiro.test(resid(S_nested_a2))

### Likelihood-ratio based pseudo-R-squared of final model
r.squaredLR(S_nested_a2)
```

Supplementary Figures:

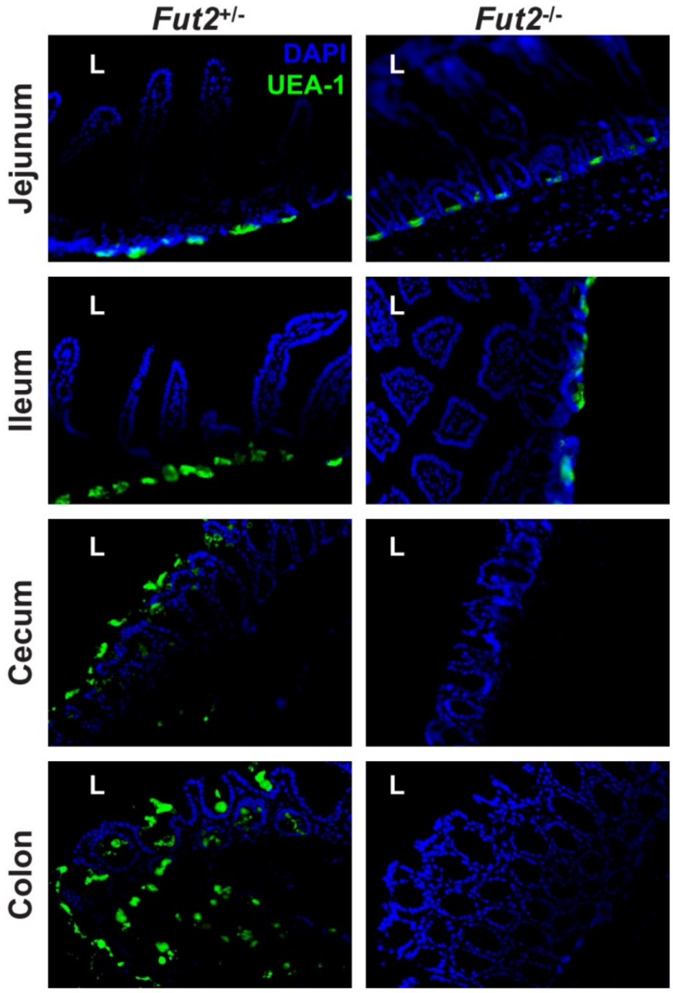

Figure S1: Validation of *secretor* status along the gastrointestinal tract via fluorescently labled *Ulex europaeus* agglutinin-1 staining (UEA-1, green) specifc to α-1,2-fucosylated glycan residues and 4,6-Diamidin-2-phenylindol (DAPI, blue) counterstaining (100×magnification, L- Lumen). *Secretor* individuals (*Fut2*$^{+/-}$) show glycan staining of Paneth cells (jejunum, ileum) and Goblet cells (cecum, colon), while *non-secretor* mice *(Fut2*$^{-/-}$) show residual staining of paneth cells in the jejunum and ileum as previously described by Goto et al. (Goto et al., 2014).

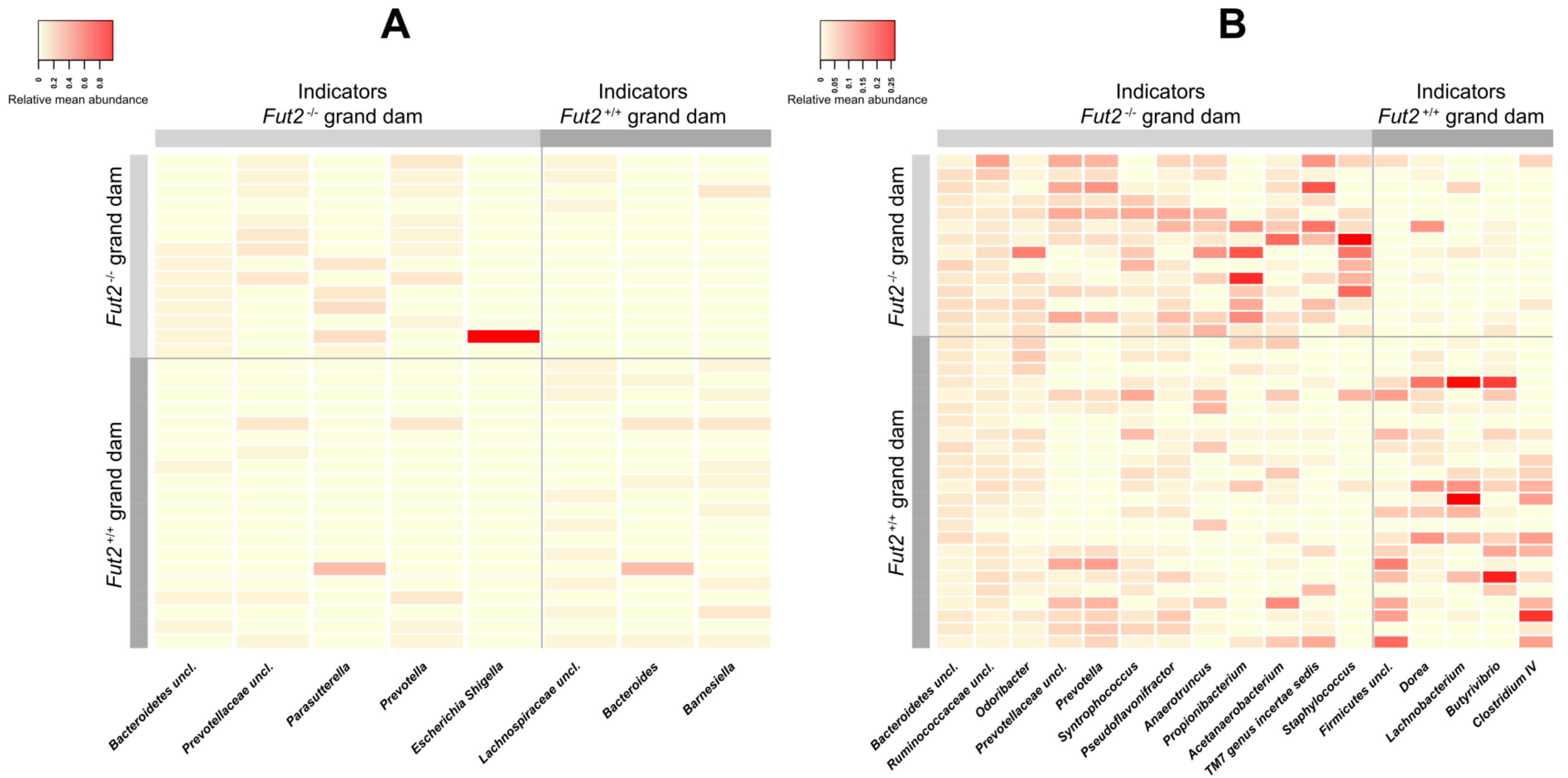

Figure S2: Abundance of significant (A) fecal and (B) mucosal indicator genera for breeding directions (q-value ≤ 0.05), corresponding to the results in Table 2. Intensity is colored as relative abundance of the respective taxon among individual mice, based on the mean abundance across time points/mucosal sites.

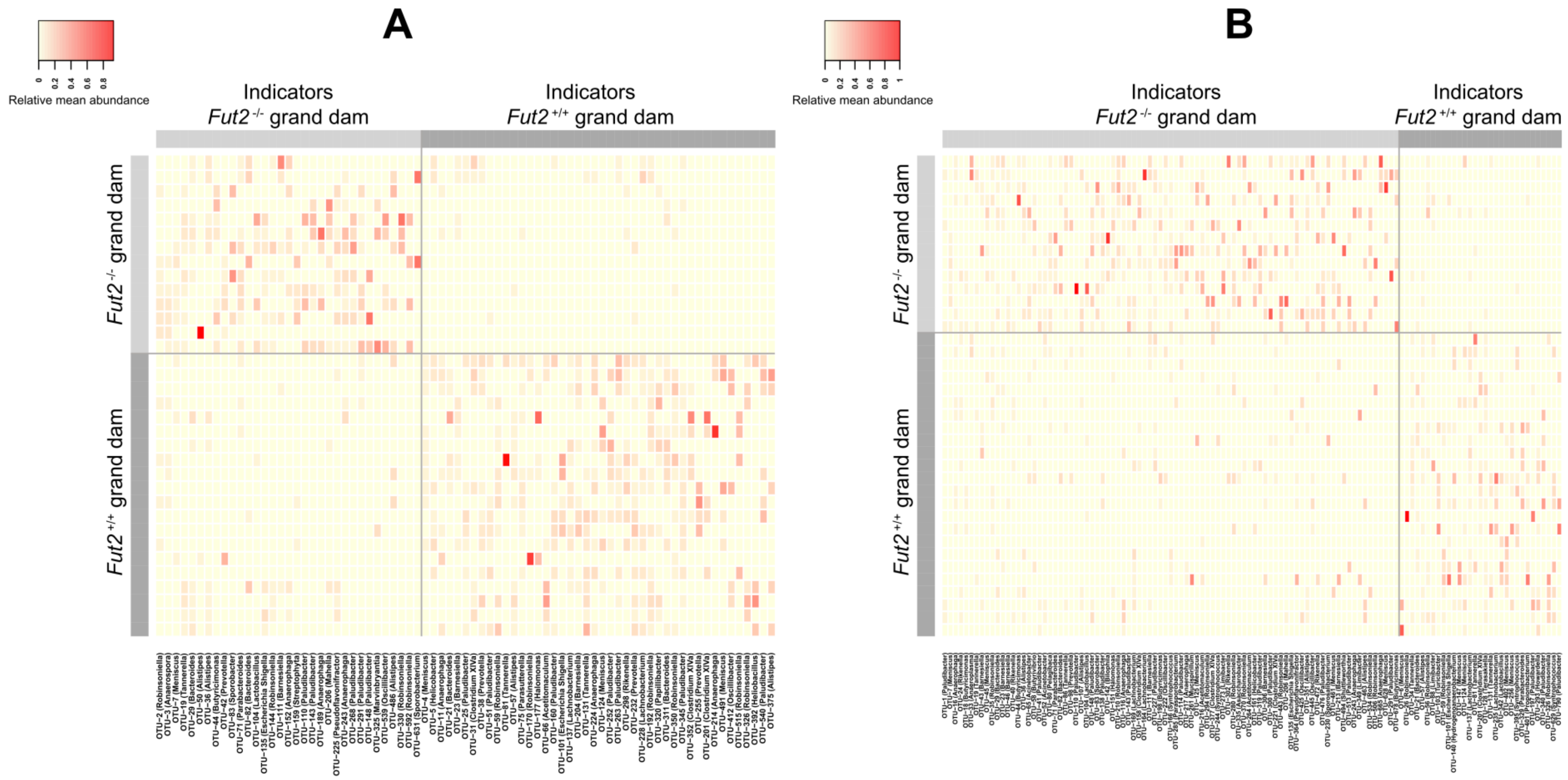

Figure S3: Abundance of significant (q-value ≤ 0.05) indicator OTUs for breeding direction in the fecal time (A) points and mucosa (B), corresponding to the results in Table S3 (A) and Table S4 (B). Intensity is colored as relative abundance of the respective taxon among individual mice, based on the mean abundance across time points/mucosal sites.

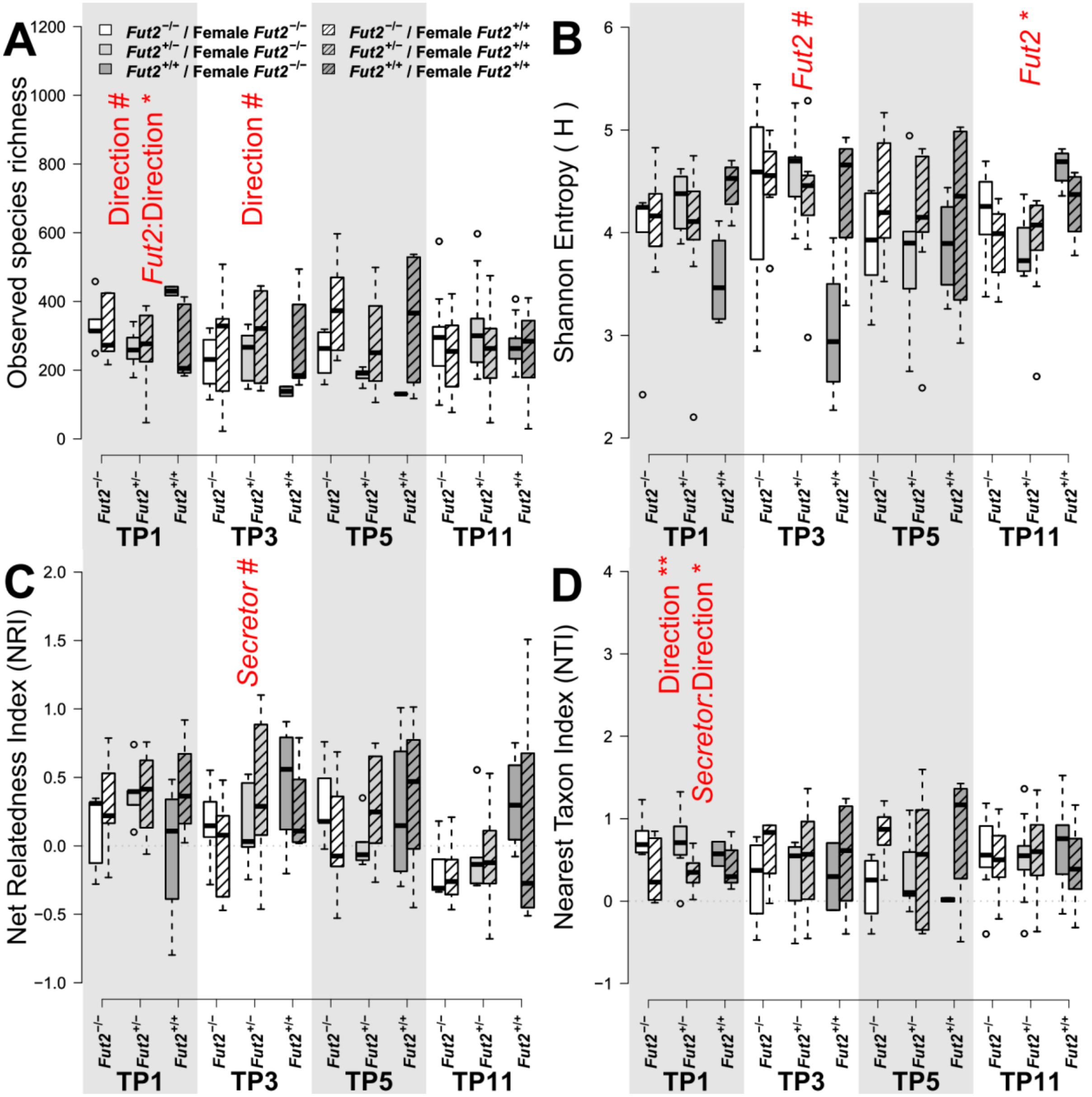

Figure S4: Alpha diversity differences of fecal bacterial communities corresponding to the results in Table S10, focusing on the number of species (A), their distribution (B), and their phylogenetic relatedness (NRI/NTI; C, D). Factors highlighted in red signify the most influential factors for the respective alpha diversity metric (*** $P<0.001$, ** $P<0.010$, * $P<0.050$, # $P<0.1000$).

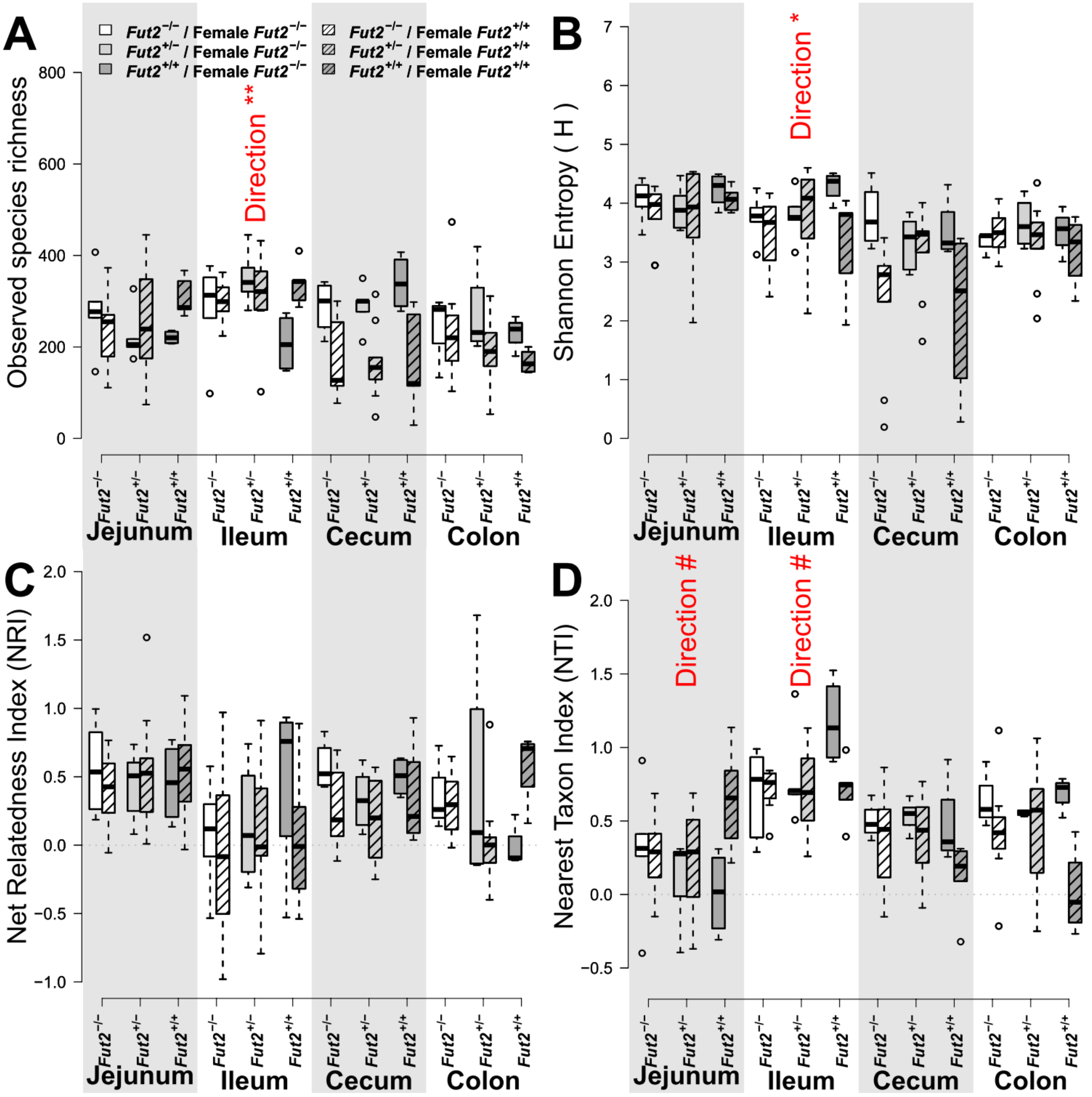

Figure S5: Alpha diversity differences of mucosa associated bacterial communities corresponding to the results in Table S11 focusing on the number of species (A), their distribution (B), and their phylogenetic relatedness (NRI/NTI; C, D). Factors highlighted in red signify the most influential factors for the respective alpha diversity metric (*** P<0.001, ** P<0.010, * P<0.050, # P<0.1000).

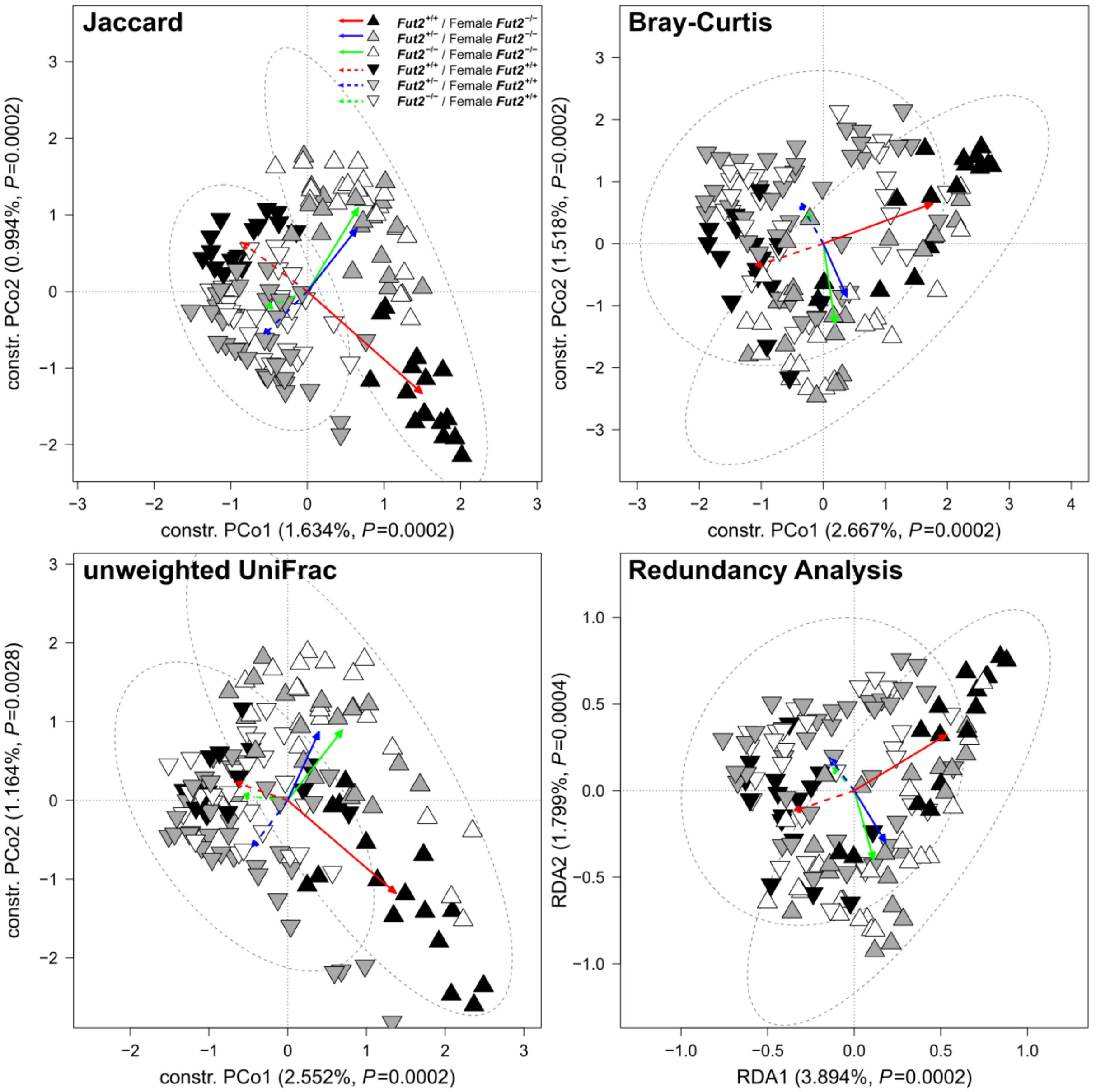

Figure S6: Community clustering among *Fut2* genotypes and breeding direction over the combined fecal time course regarding community composition (Jaccard), structure (Bray-Curtis, Redundancy Analysis/Euclidean) and phylogenetic composition (unweighted UniFrac) by constrained Principle Coordinate Analysis (arrows indicate direction of interaction *Fut2* genotpe-breeding direction, see Table 4).

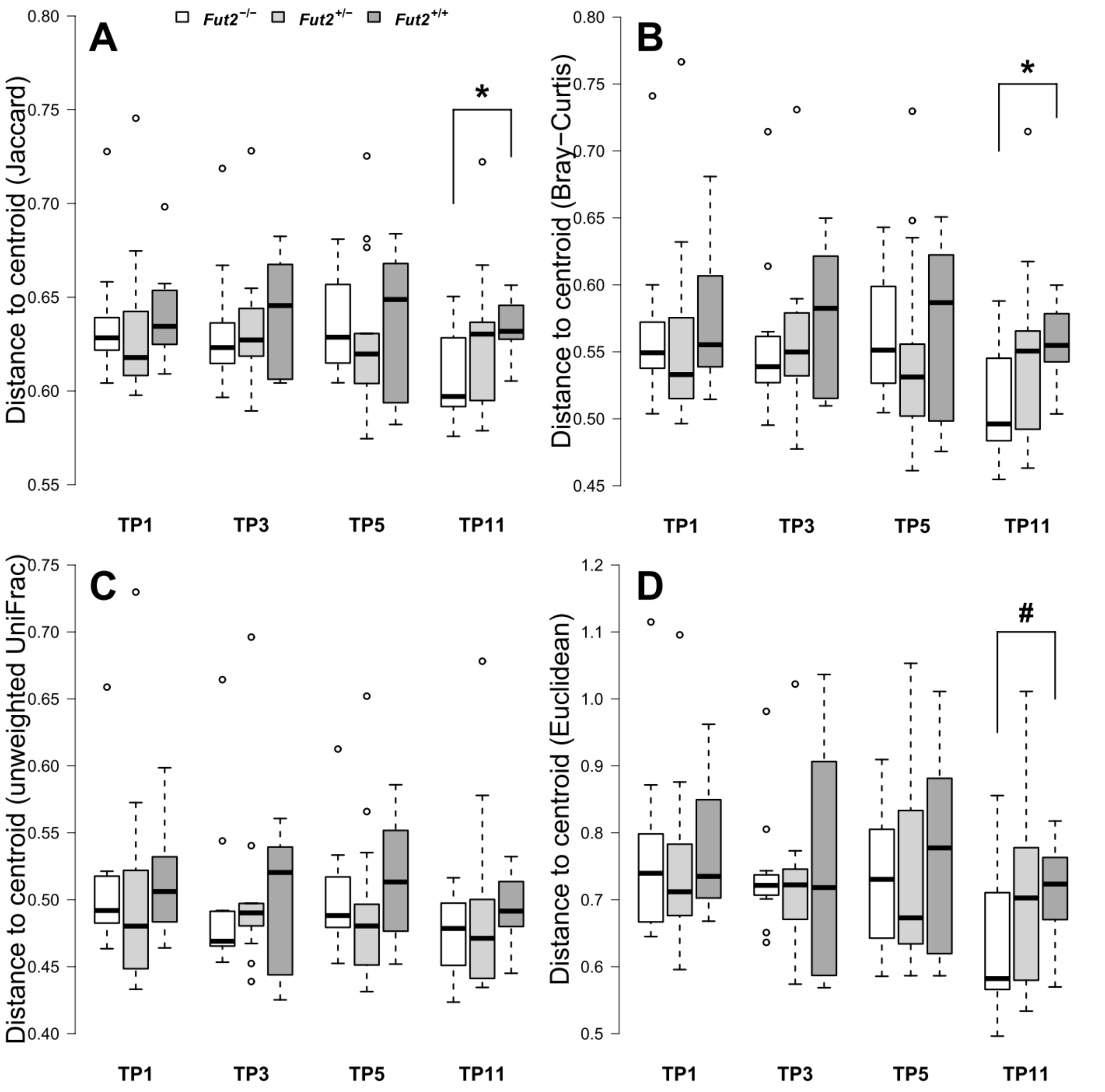

Figure S7: Community variability among *Fut2* genotypes over time regarding community composition (A), structure (B, D) and phylogenetic composition (C; *** P<0.001, ** P<0.010, * P<0.050, # P<0.1000).

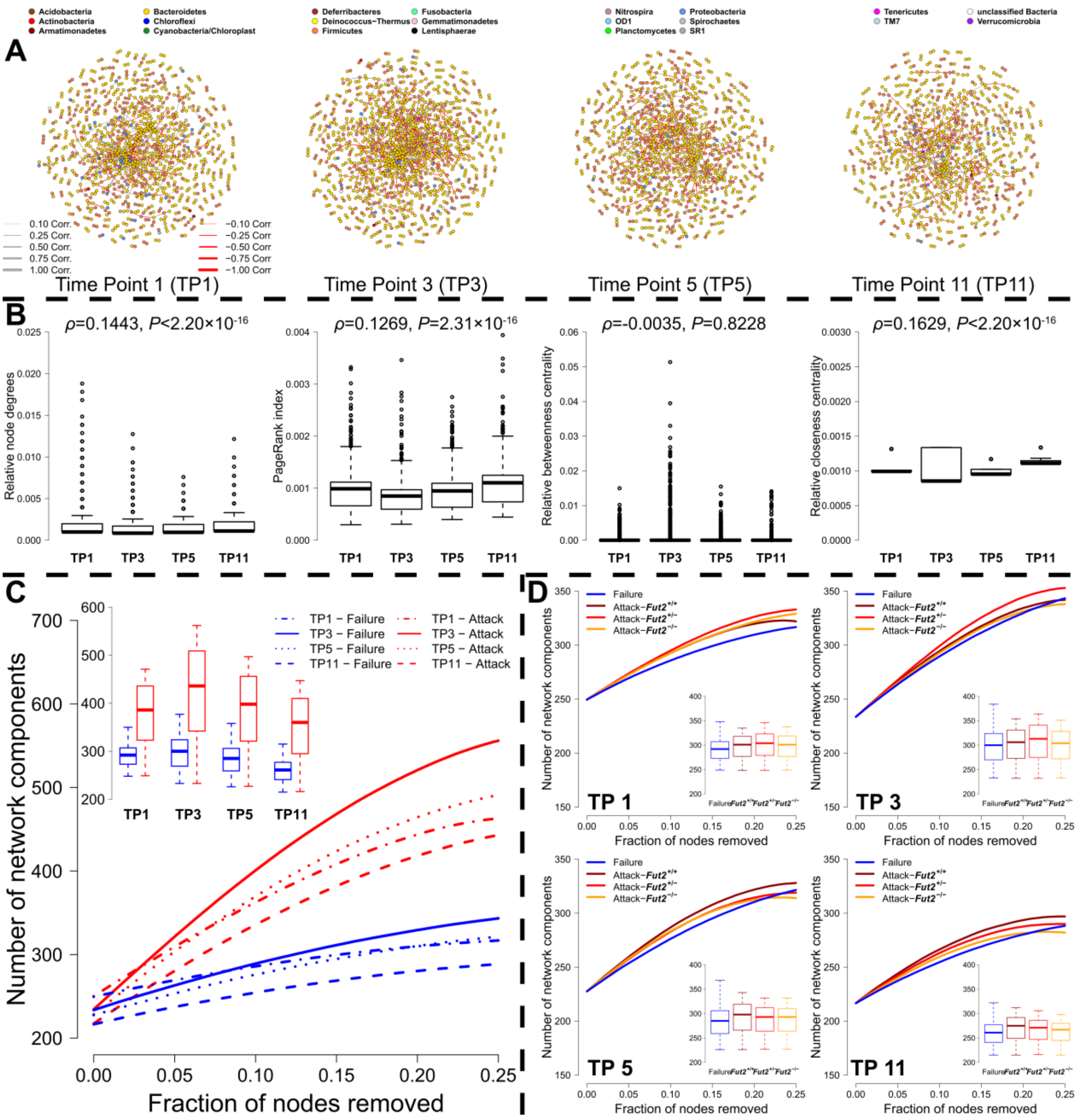

Figure S8: (A) Species correlation networks of time point 1 to 11, with a decreasing network density (TP1=0.00177, TP3=0.00145, TP5=0.00153, TP11=0.00174), as well as decreasing centralization (TP1=28.4996, TP3=9.0666, TP5=4.1775, TP11=6.8168, Kleinberg's hub score), but somehow increasing diameter (TP1=5.5521, TP3=9.7386, TP5=9.1423, TP11=6.8903). (B) Analyses of node characteristics among the interaction networks between time points focusing on the number of connections of single genera (node degree), their importance based on the quality of its connectedness (PageRank^{TM}), and the importance of single bacteria as mediators between assemblages (betweeness). (C) Analysis of network robustness based on sequential random removal (failure, blue) and targeted attack (red) on the most integrated species level OTUs (highest number of connections/degree). Plotted is the mean decay of the networks into smaller components (based on 1000 permutations) after the removal of a fraction of nodes (see Figure S10 for additional network characteristics). (D) The right panels show network decay after random removal (blue, failure), and targeted removal of either the top 25% *secretor* (*Fut2* $^{+/+}$- dark red, *Fut2* $^{+/-}$- red) or *non-secretor* (*Fut2* $^{-/-}$- yellow) associated species (mean of 1000 permutations).

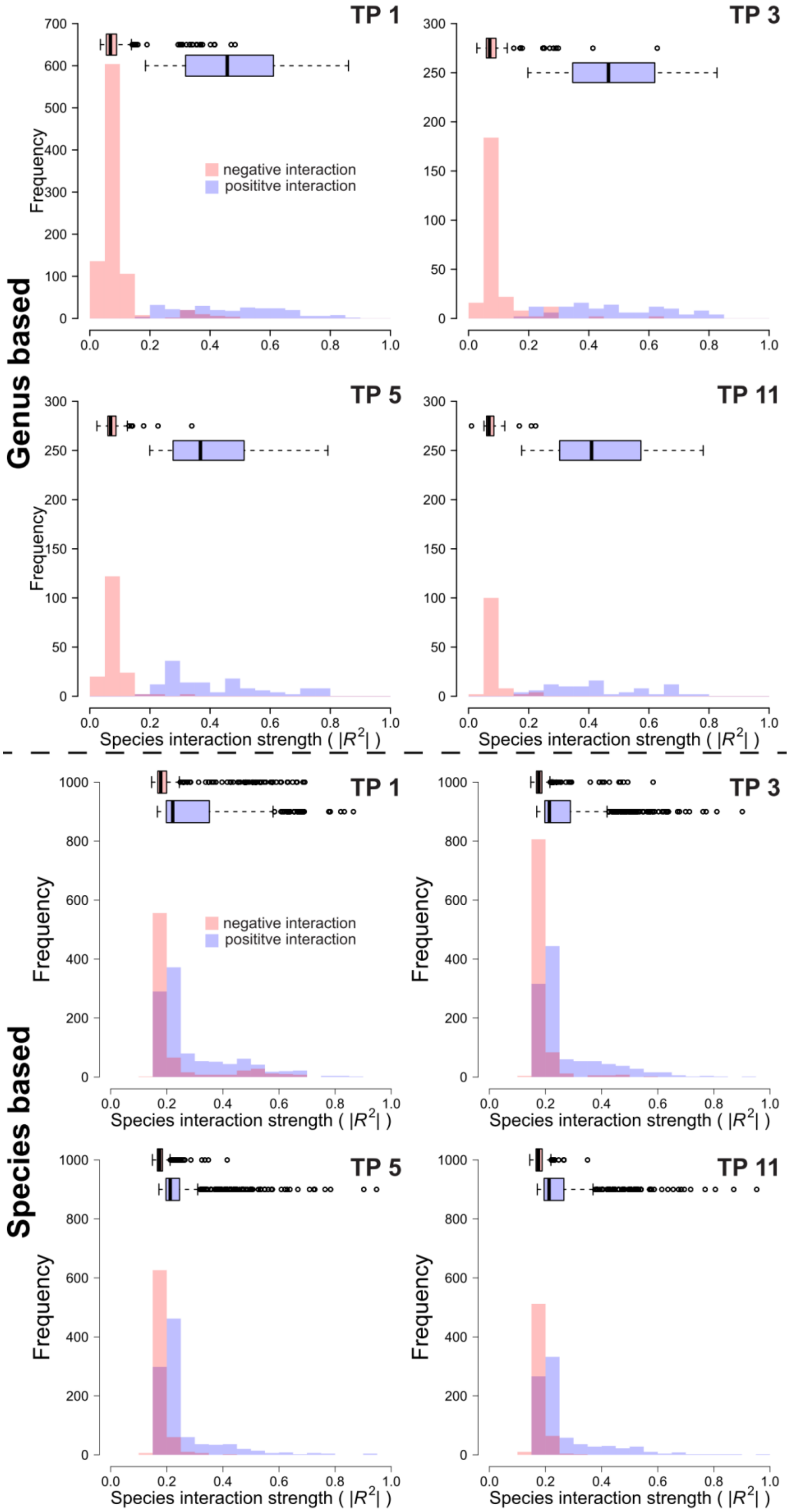
Genus based
Species based
TP 1
TP 3
TP 5
TP 11
negative interaction
posititve interaction
Frequency
Species interaction strength (|R^2|)

Figure S9: Spectrum of genera and species interactions based on significant pairwise SparCC correlations (genera networks: $P \leq 0.005$, species networks: $P \leq 0.001$). Positive interactions are consistently stronger than negative interactions in the genera- and species networks (genera: TP1-W=231200, $P<2.20 \times 10^{-16}$; TP3-W=29756, $P<2.20 \times 10^{-16}$; TP5-W=23260, $P<2.20 \times 10^{-16}$; TP11-W=10416, $P<2.20 \times 10^{-16}$; species: TP1-W=635290, $P<2.20 \times 10^{-16}$; TP3-W=901910, $P<2.20 \times 10^{-16}$; TP5-W=639410, $P<2.20 \times 10^{-16}$; TP11-W=443500, $P<2.20 \times 10^{-16}$)

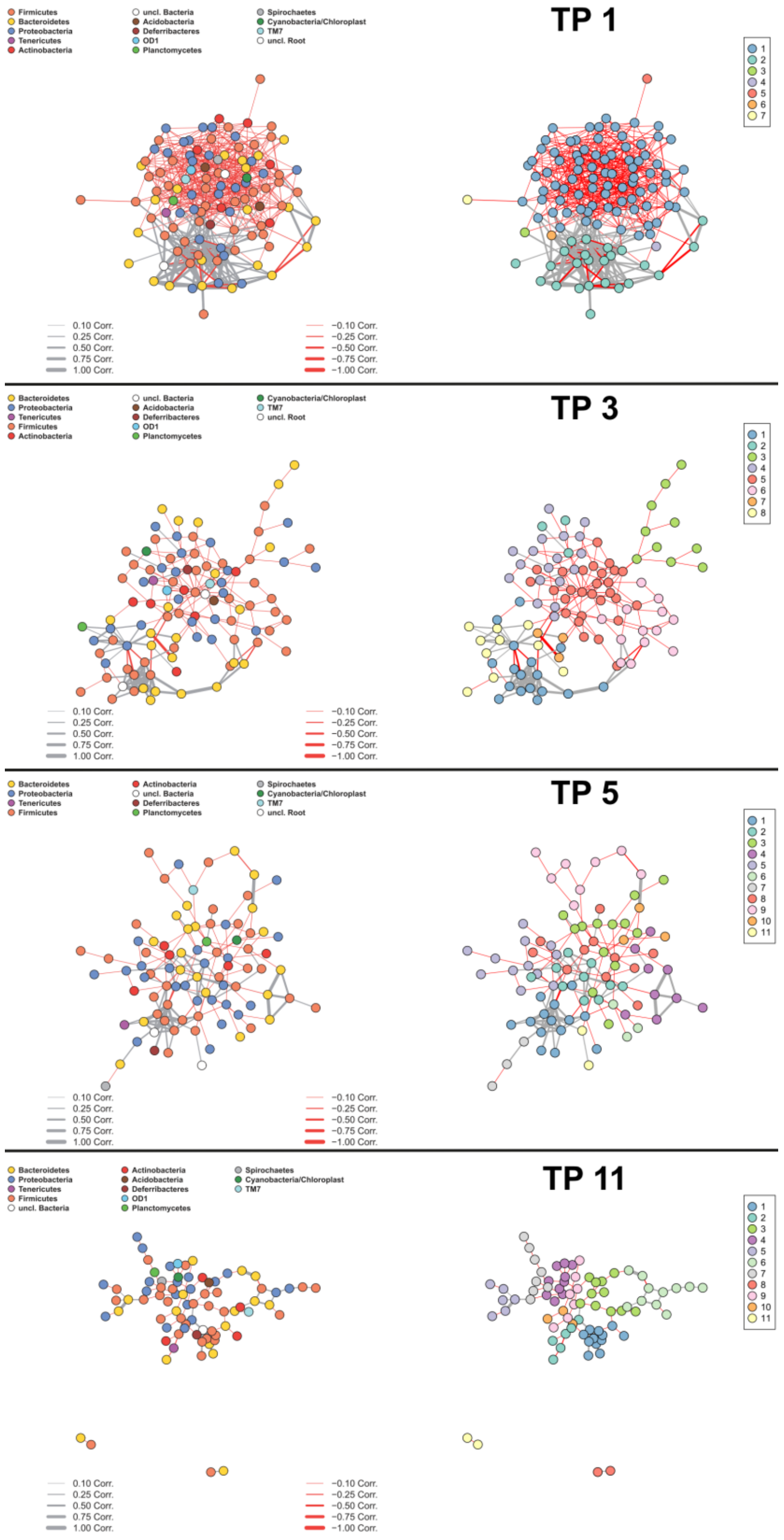

Figure S10: Analysis of network modularity based on the importance of single interactions in each time point of the genera networks (modularity; TP1: 0.311, TP3: 0.488, TP5: 0.580, TP11: 0.562). The network layout is based on the Kamada-Kawai-algorithm and the right side shows the different modules based on on edge betweenness in color code (Clauset et al., 2004;Newman and Girvan, 2004).

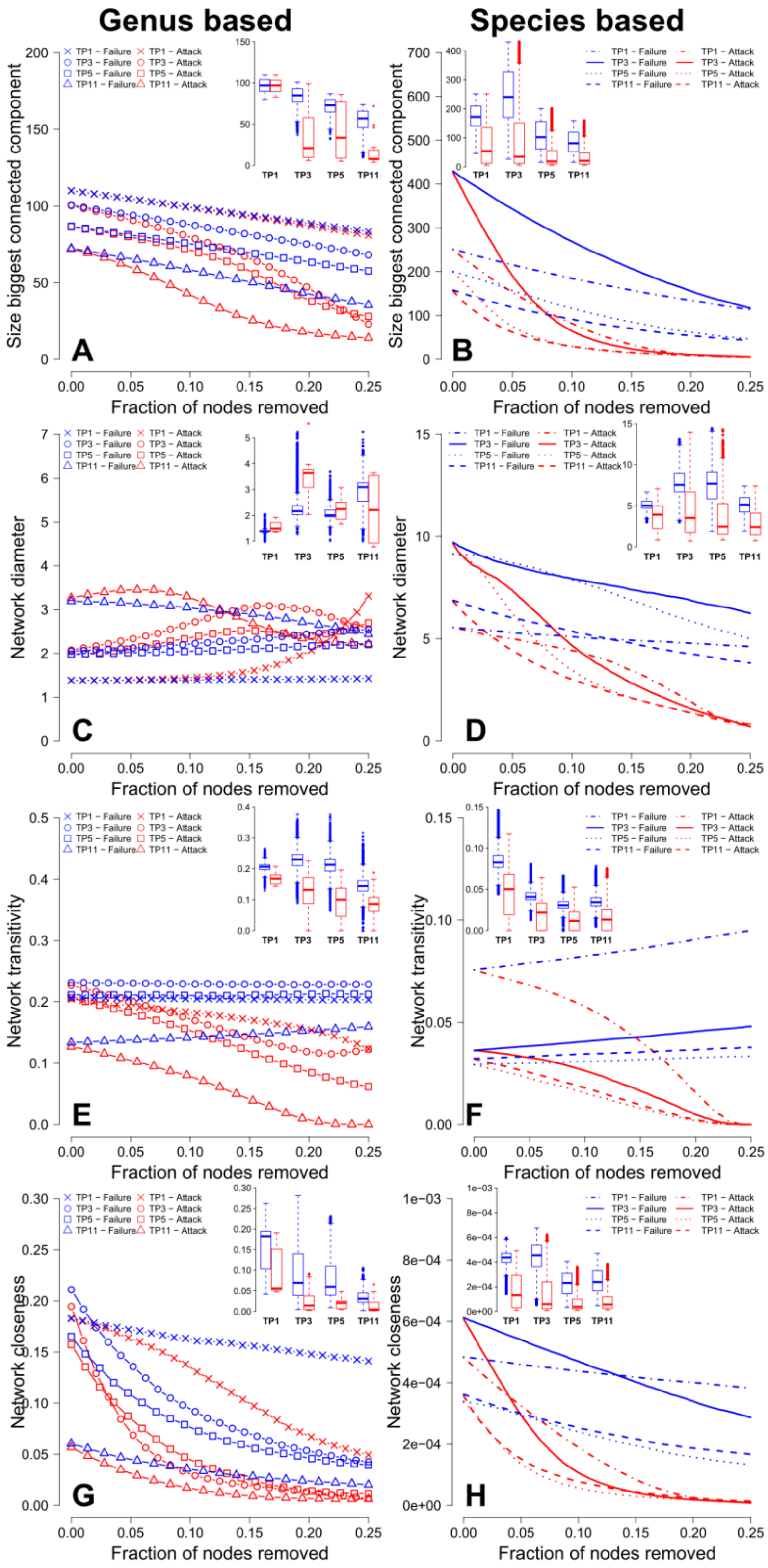

Figure S11: Analysis of network robustness based on sequential random removal (failure, blue) and targeted attack (red) on the most integrated genera/species (highest number of connections) measured as the decay of the networks based on the average size of the biggest connected subnetwork (A, B), network diameter (C, D), transitivity/clustering of the networks (E, F), and average node distance (G, H; closeness).

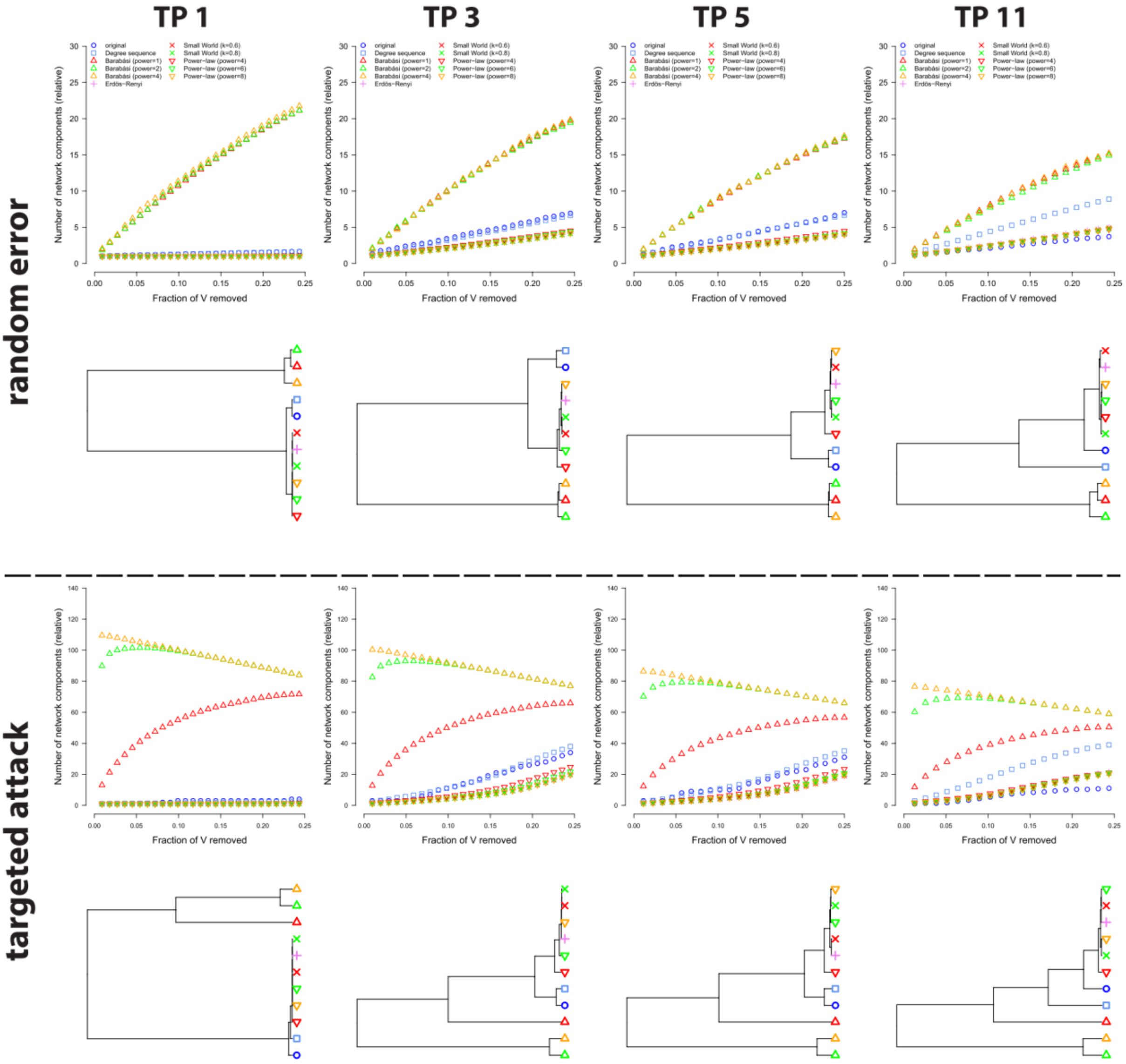

Figure S12: Analysis of network robustness based on sequential random removal (network failure) and targeted attack on the most integrated genera (highest degree). Random networks of different characteristics, but similar size were constructed for each time point 100 times and sequential removal of up to 25% of vertices was performed by removal of the most connected node (targeted attack) or iteratively 1000 times for the measurement of network decay by random error (mean over 1000 iterations). Dendrograms visualize the euclidean distance between decay profiles of the original (blue circle) and simulated networks (other symbols). Network decay is based on the relative average number of subnetworks (number of network components, see Table S11).

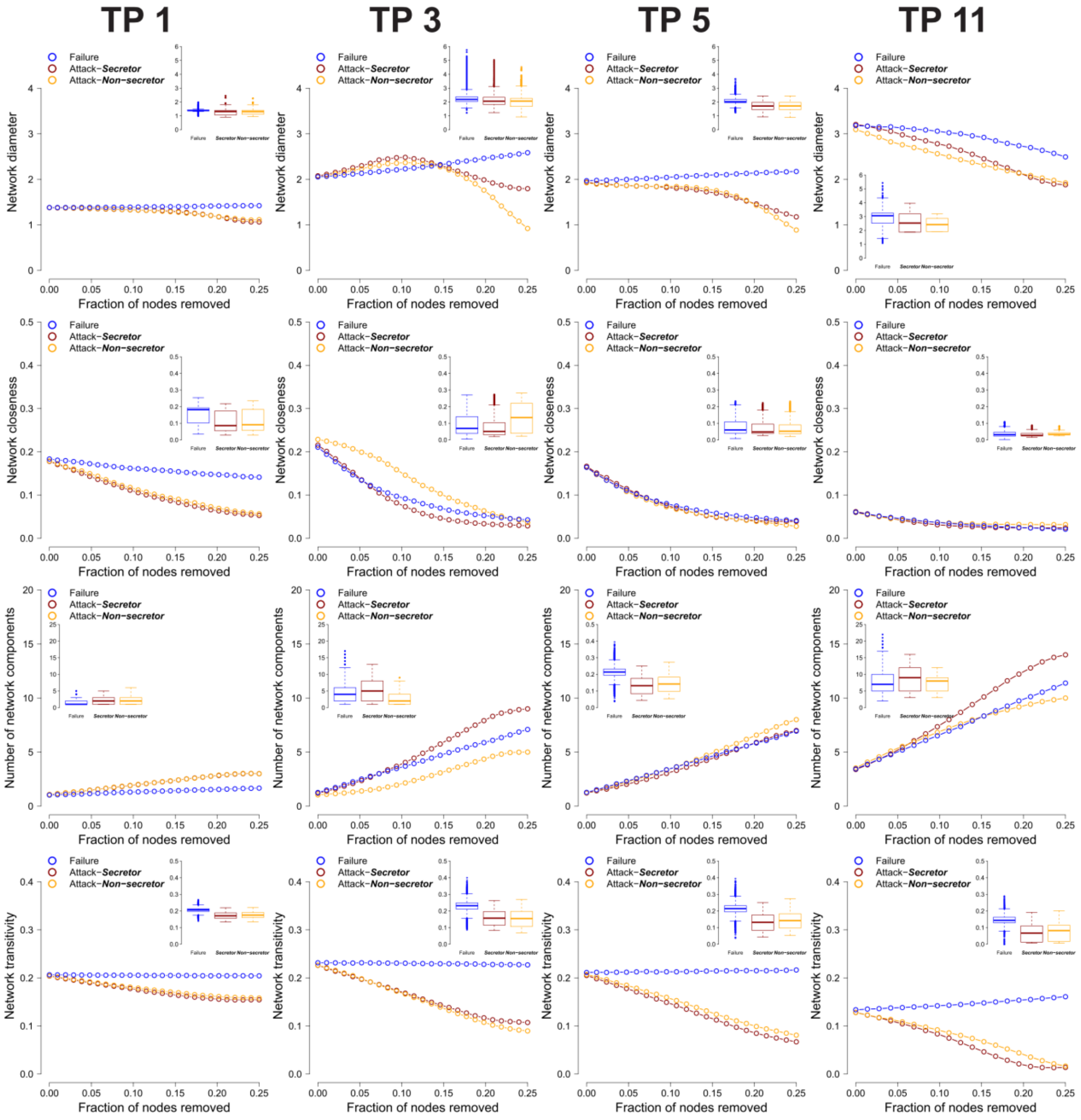

Figure S13: Average network characteristics (network diameter, closeness centrality, number of subnetworks, transitivity) under random node removal (blue) and targeted removal of the upper 25% of *secretor* associated consensus genera (dark red) and *non-secretor* associated bacteria (yellow), based on the average of 1000 iterations.

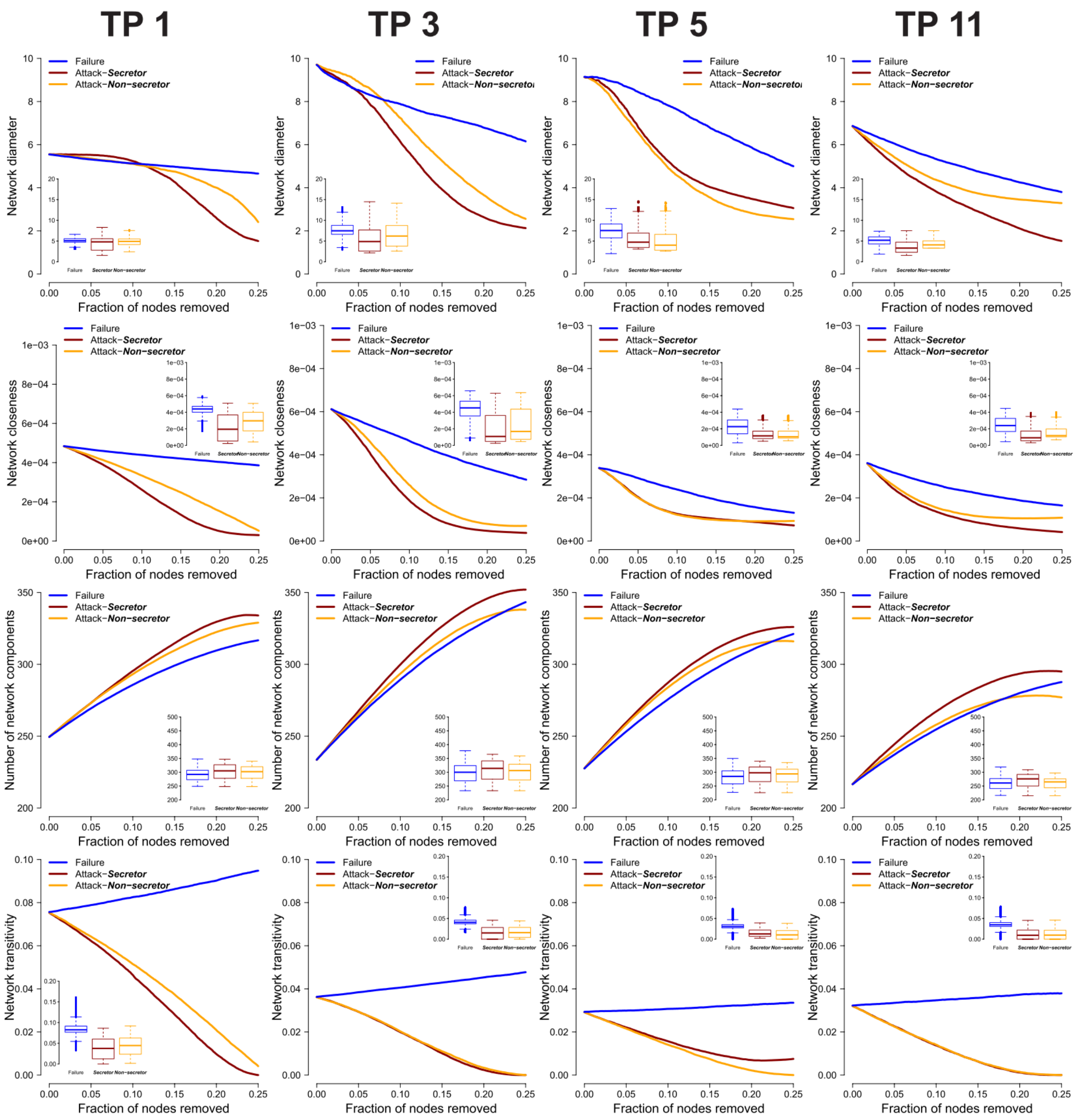

Figure S14: Average network characteristics (network diameter, closeness centrality, number of subnetworks, transitivity) under random node removal (blue) and targeted removal of the upper 25% of *secretor* associated species level OTUs (dark red) and *non-secretor* associated bacteria (orange), based on the average of 1000 iterations.

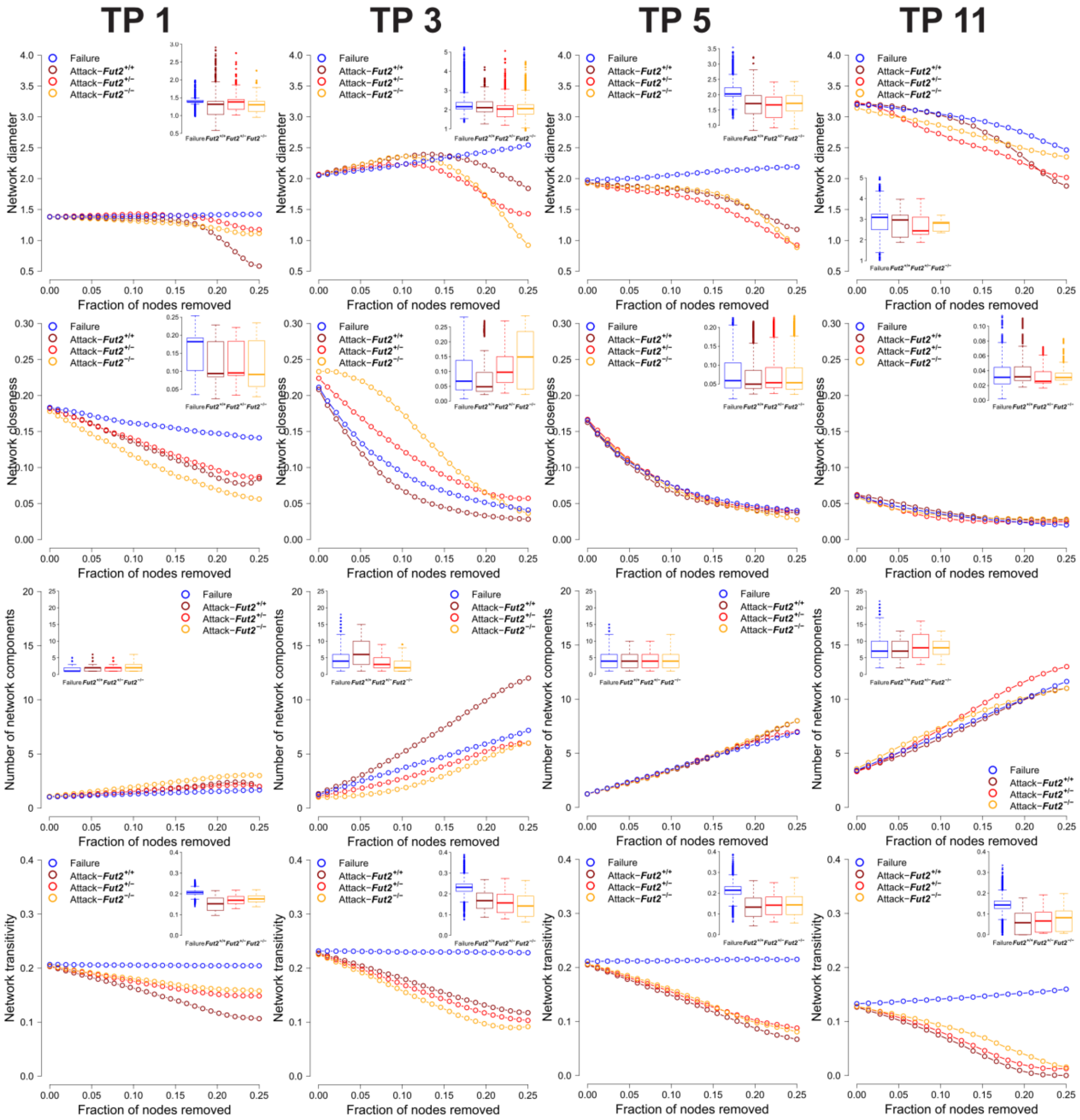

Figure S15: Network characteristics (number of subnetworks, size of the biggest subnetwork, transitivity) under random node removal (blue) and targeted removal of the top 25% of consensus genera associated to the *Fut2* $^{-/-}$ (dark red), *Fut2* $^{+/-}$ (red) or *Fut2* $^{-/-}$ associated bacteria (yellow), based on the average of 1000 iterations.

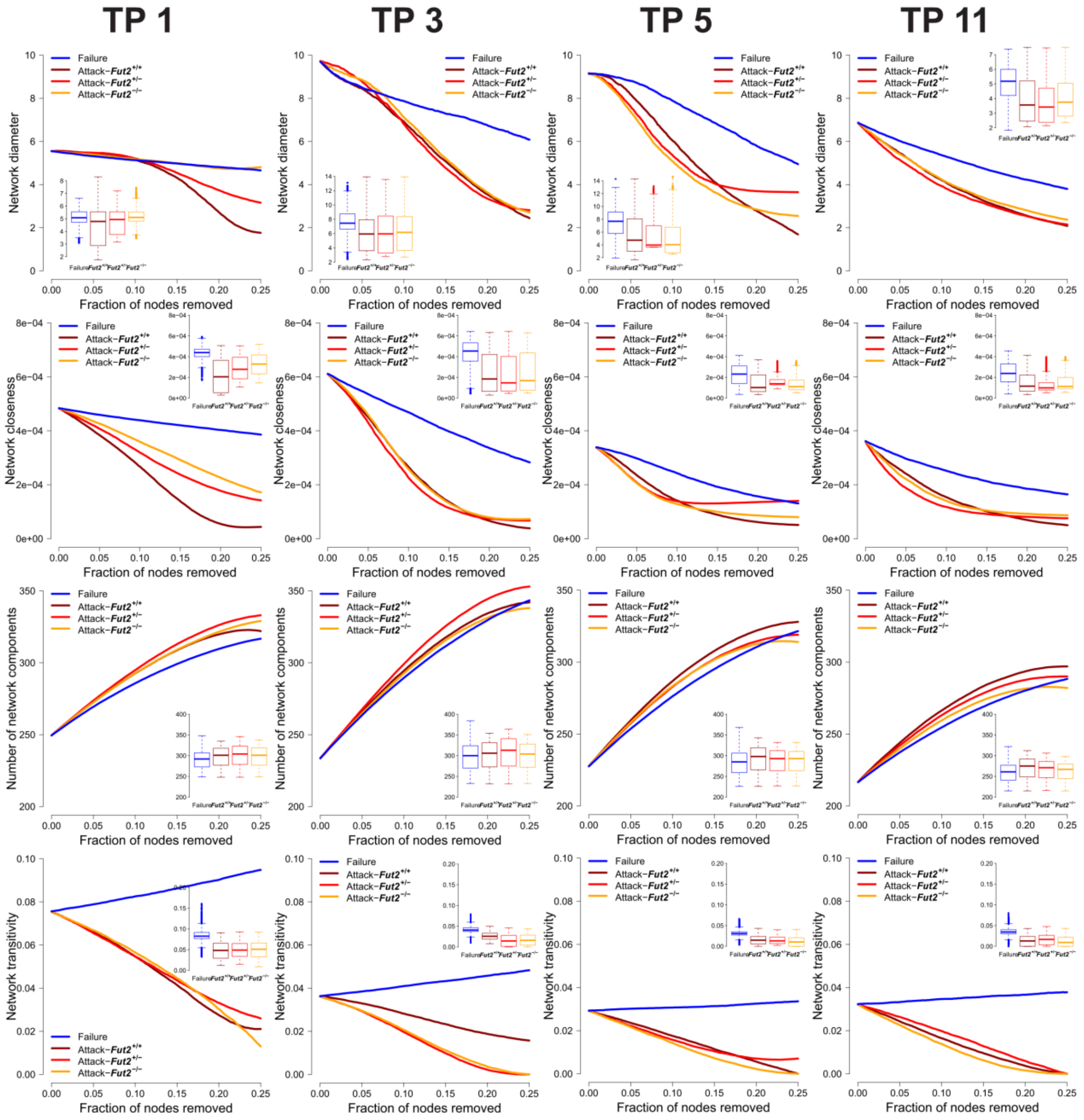

Figure S16: Network characteristics (number of subnetworks, size of the biggest subnetwork, transitivity) under random node removal (blue) and targeted removal of the top 25% of species level OTUs associated to the *Fut2* $^{-/-}$ (dark red), *Fut2* $^{+/-}$ (red) or *Fut2* $^{-/-}$ associated bacteria (yellow), based on the average of 1000 iterations.

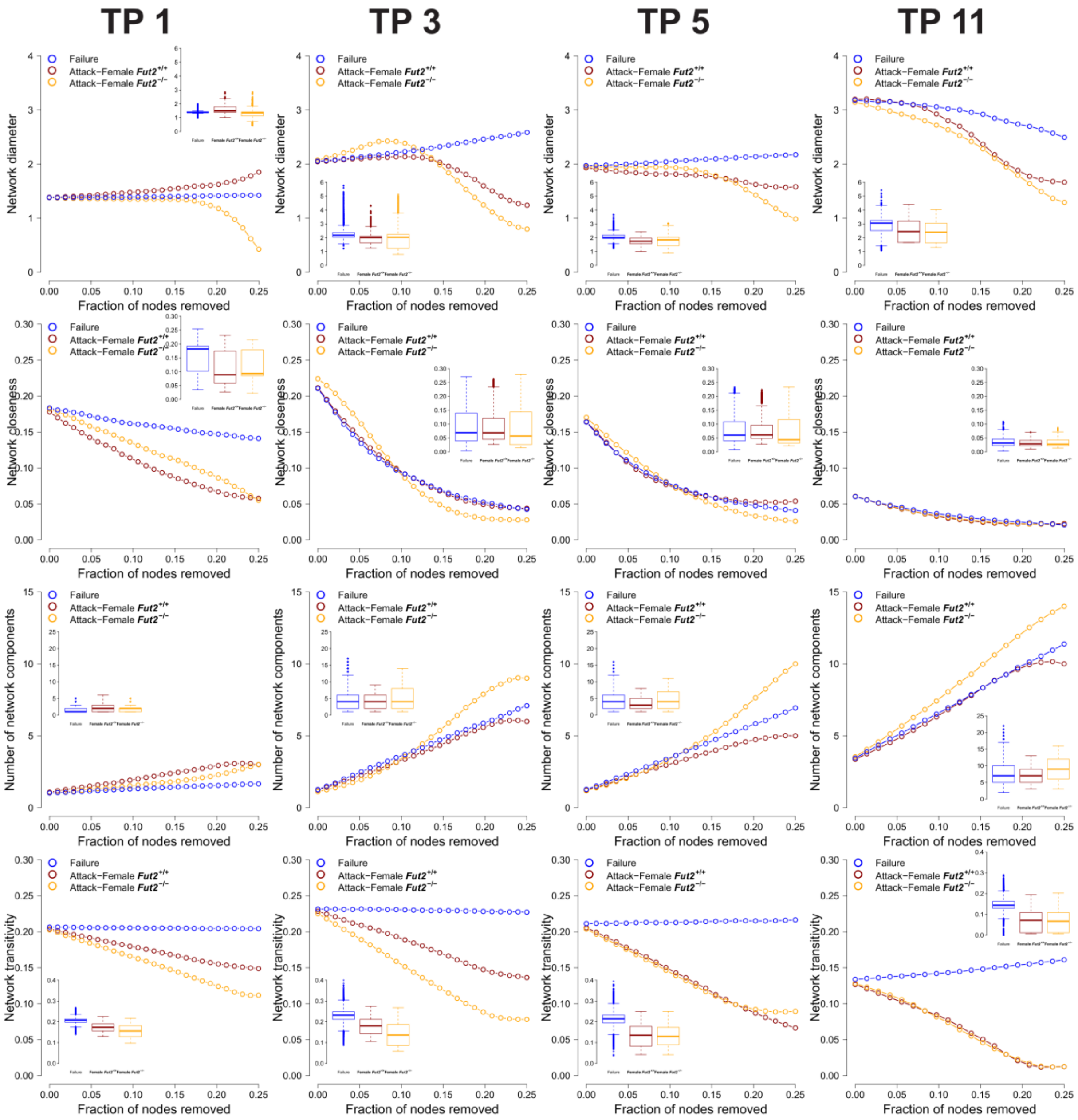

Figure S17: Network characteristics (number of subnetworks, size of the biggest subnetwork, transitivity) under random node removal (blue) and targeted removal of consensus genera associated to the *Fut2* $^{-/-}$ grand dam breeding line (dark red) or bacteria associated to *Fut2* $^{+/+}$ grand dam breeding line (yellow), based on the average of 1000 iterations.

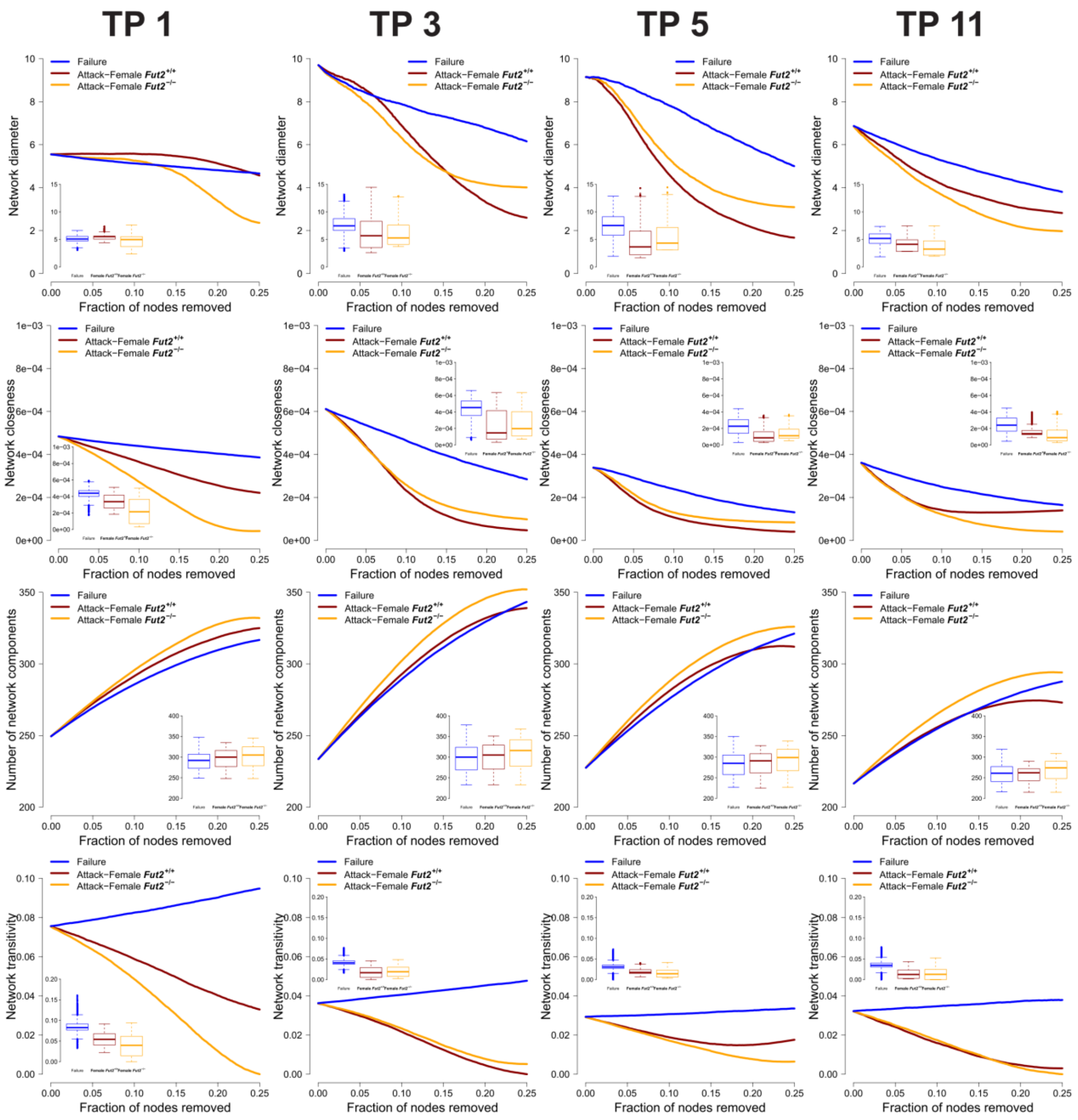

Figure S18: Network characteristics (number of subnetworks, size of the biggest subnetwork, transitivity) under random node removal (blue) and targeted removal of species level OTUs associated to the *Fut2* $^{-/-}$ grand dam breeding line (dark red) or bacteria associated to *Fut2* $^{+/+}$ grand dam breeding line (yellow), based on the average of 1000 iterations.

Supplementary Tables:

Table S1: Overview of individual mice with their respective sex, genotype, breeding cage and direction. Cage and samples collected from the respective fecal time points and tissues of the gastrointestinal tract (sampled marked with ×). The last row summarizes the table contents.

Mouse ID	Sex	*Fut2* Genotype	Breeding Cage ID	Breeding direction	Cage ID	Fecal TP1	Fecal TP3	Fecal TP5	Fecal TP11	Jejunum	Ileum	Cecum	Colon
137	M	-/-	1	*Fut2* $^{+/+}$	1	×	×	×	×	×	×	×	×
139		-/-		grand dam		×	×	×	×	×	×	×	×
136		+/-				×	×	×	×	×	×	×	×
138		+/-				×	×	×	×	×	×	×	×
135		+/+				×	×	×	×	×	×	×	×
121	M	-/-	2	*Fut2* $^{+/+}$	4	×	×	×	×	×	×	×	×
125		-/-		grand dam		×	×	×	×	×	×	×	×
124		+/-				×	×	×	×	×	×	×	×
123		+/+				×	×	×	×	×	×	×	×
110	F	-/-	3	*Fut2* $^{+/+}$	9	×	×	×	×	×	×	×	×
109		+/-		grand dam		×	×	×	×	×	×	×	×
108		+/+				×	×	×	×	×	×	×	×
132	F	-/-	1	*Fut2* $^{+/+}$	13	×	×	×	×	×	×	×	×
131		+/-		grand dam		×	×	×	×	×	×	×	×
133		+/-				×	×	×	×	×	×	×	×
134		+/-				×	×	×	×	×	×	×	×
169	M	-/-	4	*Fut2* $^{-/-}$	35	×	×	×	×	×	×	×	×
168		+/-		grand dam		×	×	×	×	×	×	×	×
182	F	-/-	5	*Fut2* $^{+/+}$	43					×	×	×	×
183		-/-		grand dam							×	×	×
185		+/-										×	
184		+/+									×	×	×
203	M	-/-	5	*Fut2* $^{+/+}$	50	×	×	×	×	×	×	×	×
204		+/-		grand dam		×	×	×	×	×	×	×	×
205		+/-				×	×	×	×	×	×	×	×
206		+/+				×	×	×	×	×	×	×	×
220	M	-/-	6	*Fut2* $^{-/-}$	56	×	×	×	×		×	×	×

221		+/-		grand dam		×	×	×	×	×	×	×	×
223	F	-/-	6	*Fut2* $^{-/-}$	57	×	×	×	×		×	×	×
222		+/-		grand dam		×	×	×	×		×	×	×
224	M	-/-	7	*Fut2* $^{-/-}$	58	×	×	×	×	×	×	×	×
226		+/-		grand dam		×	×	×	×	×	×	×	×
225		+/+				×	×	×	×		×	×	×
227		+/+				×	×	×	×	×	×	×	×
228		+/+				×	×	×	×	×	×	×	×
230	F	-/-	7	*Fut2* $^{-/-}$	59	×	×	×	×	×		×	×
229		+/-		grand dam		×	×	×	×	×	×	×	×
231		+/+				×	×	×	×	×	×	×	×
∑ 38		*Fut2*$^{-/-}$ ∑ 14<br>*Fut2*$^{+/-}$ ∑ 15<br>*Fut2*$^{+/+}$ ∑ 9	∑ 7	*Fut2*$^{-/-}$ GD: ∑ 5<br>*Fut2*$^{+/+}$ GD: ∑ 6	∑ 11	∑ 34	∑ 34	∑ 34	∑ 34	∑ 31	∑ 36	∑ 38	∑ 37

Table S2: Analysis of the major bacterial phyla within the different gastrointestinal tract locations incorporating the *Fut2* genotype/*secretor* status, or the mouse breeding lineage (founded by *Fut2* $^{-/-}$ or *Fut2* $^{+/+}$ grand dam).

Phylum	Tissue location	Factor	*DF*	*F*-value	*P*-value
Firmicutes	Ileum#	*Intercept*	1,23	392.907	<0.0001
		Fut2	2,23	2.909	0.0747
	Jejunum‡	*Intercept*	1,21	90.712	<0.0001
	Cecum	*Intercept*	1,27	224.717	<0.0001
		Direction	1,9	2.998	0.1174
	Colon‡	*Intercept*	1,26	429.929	<0.0001
Bacteroidetes	Ileum*	*Intercept*	1,23	46.461	<0.0001
		Fut2	2,23	2.928	0.0736
	Jejunum***	*Intercept*	1,21	60.061	<0.0001
	Cecum	*Intercept*	1,27	794.952	<0.0001
		Direction	1,9	5.517	0.0434
	Colon	*Intercept*	1,26	274.419	<0.0001
Proteobacteria†	Ileum	*Intercept*	1,23	321.686	<0.0001
		Direction	1,9	0.611	0.4546
		Secretor	1,23	1.726	0.2019
		Direction × *Secretor*	1,23	7.236	0.0131
	Jejunum	*Intercept*	1,19	387.581	<0.0001
		Fut2	2,19	11.360	0.0006
	Cecum	*Intercept*	1,26	1400.570	<0.0001
		Secretor	1,26	6.613	0.0162
	Colon	*Intercept*	1,26	146.536	<0.0001

* X^2 transformed; # $X^{1/4}$ transformed; † log(X+1) transformed; ‡ $X^{1/2}$ transformed; *** X^3 transformed

Table S3: Indicator species level OTUs associated to *Fut2* genotype/*secretor* status, or the mouse breeding direction (founded by *Fut2* $^{-/-}$ or *Fut2* $^{+/+}$ grand dam) in the single and combined fecal time points (gray shading highlights taxa with multiple associations of a similar category).

Factors	Time points	OTU-ID	Association	*IndVal.g*	*P*-value	*q*-value	OTU Classification (RDP)
Fut2	TP1	-	-	-	-	-	-
	TP3	-	-	-	-	-	-
	TP5	-	-	-	-	-	-
	TP11	-	-	-	-	-	-
	TP1-TP11	-	-	-	-	-	-
Secretor	TP1	-	-	-	-	-	-
	TP3	-	-	-	-	-	-
	TP5	-	-	-	-	-	-
	TP11	-	-	-	-	-	-
	TP1-TP11	-	-	-	-	-	-

Breeding Direction	TP1	2	*Fut2*$^{-/-}$ dam	0.9338	0.0002	0.0374	*Robinsoniella(100)*
		110		0.7559	0.0003	0.0374	*Paludibacter(100)*
		23	*Fut2*$^{+/+}$ dam	0.8837	0.0003	0.0374	*Barnesiella(100)*
		59		0.9159	0.0001	0.0374	*Robinsoniella(100)*
	TP3	42	*Fut2*$^{-/-}$ dam	0.8718	0.0003	0.0460	*Prevotella(100)*
		143		0.7559	0.0001	0.0460	*Paludibacter(100)*
		268		0.7071	0.0004	0.0490	*Paludibacter(100)*
		51	*Fut2*$^{+/+}$ dam	0.8726	0.0003	0.0460	*Paludibacter(100)*
		204		0.8367	0.0003	0.0460	*Barnesiella(100)*
	TP5	-	-	-	-	-	-
	TP11	7	*Fut2*$^{-/-}$ dam	0.8981	0.0001	0.0150	*Meniscus(100)*
		71		0.8583	0.0004	0.0301	*Parabacteroides(100)*
		83		0.7559	0.0004	0.0301	*Sporobacter(100)*
		104		0.8338	0.0001	0.0150	*Lactobacillus(100)*
		110		0.7496	0.0003	0.0301	*Paludibacter(100)*
		143		0.8018	0.0001	0.0150	*Paludibacter(100)*
		268		0.8018	0.0001	0.0150	*Paludibacter(100)*
		59	*Fut2*$^{+/+}$ dam	0.8062	0.0003	0.0301	*Robinsoniella(100)*
	TP1-TP11	2	*Fut2*$^{-/-}$ dam	0.7918	0.0001	0.0029	*Robinsoniella(100)*
		3		0.7508	0.0016	0.0141	*Anaerospora(100)*
		7		0.8089	0.0001	0.0029	*Meniscus(100)*
		19		0.7626	0.0022	0.0180	*Tannerella(100)*
		29		0.6941	0.0008	0.0091	*Bacteroides(100)*
		36		0.7151	0.0001	0.0029	*Alistipes(100)*
		42		0.7197	0.0052	0.0337	*Prevotella(100)*
		44		0.7035	0.0002	0.0037	*Butyricimonas(100)*
		50		0.4636	0.0033	0.0233	*Alistipes(100)*
		71		0.6843	0.0002	0.0037	*Parabacteroides(100)*
		82		0.5647	0.0009	0.0098	*Bacteroides(100)*
		83		0.5669	0.0001	0.0029	*Sporobacter(100)*
		104		0.5774	0.0033	0.0233	*Lactobacillus(100)*
		110		0.5519	0.0010	0.0104	*Paludibacter(100)*
		111		0.5787	0.0020	0.0173	*Barnesiella(100)*
		135		0.6445	0.0015	0.0135	*Escherichia/Shigella(100)*
		143		0.6980	0.0001	0.0029	*Paludibacter(100)*
		144		0.6337	0.0043	0.0291	*Robinsoniella(100)*
		148		0.5000	0.0001	0.0029	*Paludibacter(100)*
		152		0.6259	0.0001	0.0029	*Anaerophaga(100)*
		159		0.6210	0.0026	0.0202	*Streptophyta(100)*
		189		0.4867	0.0002	0.0037	*Anaerophaga(100)*
		206		0.4874	0.0024	0.0190	*Mahella(100)*
		225		0.5878	0.0009	0.0098	*Pseudoflavonifractor(100)*
		243		0.6194	0.0003	0.0050	*Anaerophaga(100)*
		268		0.6376	0.0001	0.0029	*Paludibacter(100)*
		291		0.5282	0.0032	0.0233	*Paludibacter(100)*
		325		0.4665	0.0005	0.0067	*Marvinbryantia(100)*
		330		0.4145	0.0076	0.0473	*Robinsoniella(100)*
		486		0.4736	0.0027	0.0207	*Alistipes(100)*
		539		0.4866	0.0002	0.0037	*Oscillibacter(100)*
		592		0.4668	0.0002	0.0037	*Robinsoniella(100)*
		631		0.4381	0.0007	0.0082	*Sporobacterium(100)*
		4	*Fut2*$^{+/+}$ dam	0.7788	0.0004	0.0057	*Meniscus(100)*
		5		0.7473	0.0002	0.0037	*Helicobacter(100)*
		11		0.8116	0.0031	0.0230	*Anaerophaga(100)*
		21		0.8862	0.0001	0.0029	*Bacteroides(100)*

		25		0.7415	0.0049	0.0322	*Paludibacter(100)*
		31		0.7104	0.0030	0.0226	*Clostridium XlVa(100)*
		38		0.7370	0.0001	0.0029	*Prevotella(100)*
		51		0.7683	0.0001	0.0029	*Paludibacter(100)*
		57		0.7719	0.0001	0.0029	*Alistipes(100)*
		59		0.7963	0.0001	0.0029	*Robinsoniella(100)*
		66		0.4928	0.0013	0.0126	*Acetitomaculum(100)*
		75		0.7432	0.0001	0.0029	*Parasutterella(100)*
		81		0.4455	0.0079	0.0485	*Tannerella(100)*
		101		0.5189	0.0004	0.0057	*Escherichia/Shigella(100)*
		124		0.5201	0.0002	0.0037	*Meniscus(100)*
		131		0.5174	0.0022	0.0180	*Tannerella(100)*
		137		0.6465	0.0002	0.0037	*Lachnobacterium(100)*
		160		0.6674	0.0004	0.0057	*Paludibacter(100)*
		170		0.5596	0.0022	0.0180	*Robinsoniella(100)*
		177		0.5765	0.0006	0.0076	*Halomonas(100)*
		192		0.6256	0.0001	0.0029	*Robinsoniella(100)*
		201		0.4330	0.0015	0.0135	*Clostridium XlVa(100)*
		204		0.6221	0.0001	0.0029	*Barnesiella(100)*
		214		0.4183	0.0014	0.0131	*Anaerophaga(100)*
		224		0.5832	0.0003	0.0050	*Anaerophaga(100)*
		228		0.5478	0.0013	0.0126	*Lachnobacterium(100)*
		232		0.5936	0.0005	0.0067	*Prevotella(100)*
		252		0.5441	0.0010	0.0104	*Paludibacter(100)*
		255		0.4472	0.0007	0.0082	*Prevotella(100)*
		263		0.5493	0.0006	0.0076	*Paludibacter(100)*
		298		0.5374	0.0053	0.0339	*Rikenella(100)*
		311		0.5554	0.0043	0.0291	*Bacteroides(100)*
		320		0.5403	0.0040	0.0279	*Papillibacter(100)*
		326		0.4743	0.0003	0.0050	*Robinsoniella(100)*
		345		0.5381	0.0024	0.0190	*Paludibacter(100)*
		350		0.5563	0.0004	0.0057	*Robinsoniella(100)*
		352		0.4974	0.0045	0.0300	*Clostridium XlVa(100)*
		375		0.4330	0.0004	0.0057	*Alistipes(100)*
		392		0.4183	0.0014	0.0131	*Heliobacillus(100)*
		412		0.4330	0.0012	0.0122	*Oscillibacter(100)*
		491		0.5000	0.0002	0.0037	*Meniscus(100)*
		515		0.4478	0.0066	0.0416	*Robinsoniella(100)*
		540		0.4729	0.0007	0.0082	*Paludibacter(100)*

Table S4: Indicator species level OTUs associated to *Fut2* genotype/*secretor* status, or the mouse breeding direction (founded by *Fut2* $^{-/-}$ or *Fut2* $^{+/+}$ grand dam) in the single and combined mucosal locations (gray shading highlights taxa with multiple associations of a similar category).

Factor	Tissue location	OTU-ID	Association	*IndVal.g*	*P*-value	*q*-value	OTU Classification (RDP)
Fut2	Jejunum	-	-	-	-	-	-
	Ileum	-	-	-	-	-	-
	Cecum	-	-	-	-	-	-
	Colon	-	-	-	-	-	-
	all locations	-	-	-	-	-	-
Secretor	Jejunum	-	-	-	-	-	-

	Ileum	-	-	-	-	-	-
	Cecum	-	-	-	-	-	-
	Colon	-	-	-	-	-	-
	all locations	38	*Non-sec*	0.6683	0.0002	0.0333	*Prevotella(100)*
		228		0.5265	0.0001	0.0333	*Lachnobacterium(100)*
Breeding direction	Jejunum	-	-	-	-	-	-
	Ileum	7	*Fut2*$^{-/-}$ dam	0.9204	0.0001	0.0151	*Meniscus(100)*
		665		0.7845	0.0001	0.0151	*Anaerophaga(100)*
	Cecum	7	*Fut2*$^{-/-}$ dam	0.8623	0.0002	0.0156	*Meniscus(100)*
		16		0.8465	0.0009	0.0492	*Anaerophaga(100)*
		83		0.7071	0.0001	0.0091	*Sporobacter(100)*
		104		0.8452	0.0001	0.0091	*Lactobacillus(100)*
		110		0.7036	0.0001	0.0091	*Paludibacter(100)*
		111		0.7112	0.0008	0.0486	*Barnesiella(100)*
		210		0.8864	0.0001	0.0091	*Robinsoniella(100)*
		294		0.7071	0.0007	0.0479	*Robinsoniella(100)*
		476		0.8018	0.0001	0.0091	*Paludibacter(100)*
		628		0.6547	0.0010	0.0497	*Meniscus(100)*
		57	*Fut2*$^{+/+}$ dam	0.8983	0.0001	0.0091	*Alistipes(100)*
	Colon	7	*Fut2*$^{-/-}$ dam	0.8480	0.0013	0.0500	*Meniscus(100)*
		71		0.8167	0.0008	0.0470	*Parabacteroides(100)*
		83		0.6547	0.0010	0.0470	*Sporobacter(100)*
		143		0.8018	0.0001	0.0070	*Paludibacter(100)*
		148		0.8018	0.0001	0.0070	*Paludibacter(100)*
		210		0.9636	0.0001	0.0070	*Robinsoniella(100)*
		217		0.7729	0.0010	0.0470	*Anaerophaga(100)*
		264		0.6547	0.0013	0.0500	*Acetitomaculum(100)*
		294		0.8256	0.0001	0.0070	*Robinsoniella(100)*
		476		0.8452	0.0001	0.0070	*Paludibacter(100)*
		628		0.7071	0.0001	0.0070	*Meniscus(100)*
	all locations	1	*Fut2*$^{-/-}$ dam	0.7637	0.0114	0.0218	*Paludibacter(100)*
		7		0.8715	0.0001	0.0007	*Meniscus(100)*
		16		0.7613	0.0009	0.0038	*Anaerophaga(100)*
		18		0.7725	0.0035	0.0084	*Butyricimonas(100)*
		19		0.7603	0.0041	0.0095	*Tannerella(100)*
		23		0.7679	0.0030	0.0080	*Barnesiella(100)*
		24		0.7546	0.0352	0.0430	*Rikenella(100)*
		29		0.6981	0.0008	0.0036	*Bacteroides(100)*
		33		0.4102	0.0246	0.0341	*Anaeroplasma(100)*
		35		0.7776	0.0001	0.0007	*Robinsoniella(100)*
		36		0.6928	0.0005	0.0026	*Alistipes(100)*
		40		0.7435	0.0152	0.0250	*Rikenella(100)*
		41		0.7623	0.0006	0.0030	*Paludibacter(100)*
		43		0.6386	0.0140	0.0242	*Barnesiella(100)*
		44		0.7492	0.0001	0.0007	*Butyricimonas(100)*
		45		0.6873	0.0249	0.0341	*Meniscus(100)*
		46		0.7703	0.0019	0.0065	*Barnesiella(100)*
		52		0.7076	0.0147	0.0245	*Acinetobacter(100)*
		58		0.6104	0.0341	0.0427	*Butyrivibrio(100)*
		65		0.5914	0.0387	0.0454	*Acinetobacter(100)*
		71		0.7951	0.0001	0.0007	*Parabacteroides(100)*
		80		0.7692	0.0002	0.0013	*Pelagibaca(100)*
		82		0.6656	0.0001	0.0007	*Bacteroides(100)*
		83		0.6931	0.0001	0.0007	*Sporobacter(100)*
		86		0.6686	0.0042	0.0096	*Tannerella(100)*

	98		0.6930	0.0009	0.0038	*Prevotella(100)*
	104		0.4567	0.0143	0.0242	*Lactobacillus(100)*
	107		0.6714	0.0007	0.0033	*Alistipes(100)*
	110		0.3692	0.0408	0.0467	*Paludibacter(100)*
	111		0.6893	0.0001	0.0007	*Barnesiella(100)*
	114		0.4613	0.0108	0.0210	*Paludibacter(100)*
	116		0.5206	0.0118	0.0223	*Anaerophaga(100)*
	125		0.4070	0.0279	0.0360	*Meniscus(100)*
	135		0.4778	0.0233	0.0330	*Escherichia/Shigella(100)*
	138		0.6614	0.0007	0.0033	*Paludibacter(100)*
	142		0.5368	0.0012	0.0047	*Bilophila(100)*
	143		0.7370	0.0001	0.0007	*Paludibacter(100)*
	148		0.6369	0.0001	0.0007	*Paludibacter(100)*
	151		0.6336	0.0004	0.0025	*Halomonas(100)*
	158		0.5537	0.0171	0.0275	*Clostridium XlVa(100)*
	161		0.4756	0.0279	0.0360	*Helicobacter(100)*
	164		0.4694	0.0185	0.0294	*Lachnobacterium(100)*
	166		0.5626	0.0222	0.0321	*Syntrophococcus(100)*
	169		0.5132	0.0353	0.0430	*Lachnobacterium(100)*
	198		0.5387	0.0214	0.0319	*Butyricimonas(100)*
	206		0.4517	0.0081	0.0165	*Mahella(100)*
	210		0.7875	0.0001	0.0007	*Robinsoniella(100)*
	213		0.3925	0.0395	0.0459	*Barnesiella(100)*
	216		0.6214	0.0001	0.0007	*Acetitomaculum(100)*
	217		0.5627	0.0015	0.0056	*Anaerophaga(100)*
	221		0.4177	0.0330	0.0417	*Clostridium XlVa(100)*
	222		0.5463	0.0037	0.0088	*Oscillibacter(100)*
	227		0.5783	0.0129	0.0233	*Paludibacter(100)*
	230		0.3863	0.0198	0.0303	*Sporosalibacterium(100)*
	243		0.4139	0.0129	0.0233	*Anaerophaga(100)*
	244		0.5804	0.0005	0.0026	*Syntrophococcus(100)*
	261		0.4815	0.0423	0.0477	*Mucispirillum(100)*
	273		0.4919	0.0001	0.0007	*Limibacter(100)*
	280		0.5088	0.0054	0.0115	*Robinsoniella(100)*
	285		0.4590	0.0099	0.0195	*Parasporobacterium(100)*
	291		0.3939	0.0142	0.0242	*Paludibacter(100)*
	294		0.6121	0.0001	0.0007	*Robinsoniella(100)*
	300		0.4940	0.0032	0.0080	*Paludibacter(100)*
	302		0.4642	0.0194	0.0301	*Rikenella(100)*
	306		0.4834	0.0167	0.0272	*Barnesiella(100)*
	319		0.4907	0.0002	0.0013	*Paludibacter(100)*
	335		0.5085	0.0012	0.0047	*Asaccharobacter(100)*
	349		0.4136	0.0425	0.0477	*Limibacter(100)*
	351		0.5131	0.0045	0.0098	*Alistipes(100)*
	364		0.5279	0.0026	0.0079	*Pseudoflavonifractor(100)*
	370		0.4901	0.0005	0.0026	*Robinsoniella(100)*
	377		0.4349	0.0023	0.0071	*Clostridium XlVa(100)*
	425		0.4382	0.0097	0.0194	*Oscillibacter(100)*
	443		0.5306	0.0001	0.0007	*Robinsoniella(100)*
	445		0.4349	0.0128	0.0233	*Oscillibacter(100)*
	461		0.4960	0.0001	0.0007	*Meniscus(100)*
	463		0.4308	0.0132	0.0234	*Alistipes(100)*
	472		0.4351	0.0032	0.0080	*Rikenella(100)*
	476		0.6062	0.0001	0.0007	*Paludibacter(100)*
	494		0.4287	0.0260	0.0349	*Shuttleworthia(100)*
	506		0.4338	0.0023	0.0071	*Robinsoniella(100)*

		513		0.4560	0.0027	0.0080	*Paludibacter(100)*
		534		0.4139	0.0322	0.0411	*Robinsoniella(100)*
		628		0.5182	0.0001	0.0007	*Meniscus(100)*
		678		0.4131	0.0078	0.0161	*Butyricimonas(100)*
		6	*Fut2*$^{+/+}$ dam	0.5249	0.0044	0.0098	*Meniscus(100)*
		21		0.6990	0.0409	0.0467	*Bacteroides(100)*
		26		0.3914	0.0214	0.0319	*Robinsoniella(100)*
		34		0.7467	0.0045	0.0098	*Tannerella(100)*
		56		0.6217	0.0385	0.0454	*Robinsoniella(100)*
		57		0.7825	0.0001	0.0007	*Alistipes(100)*
		59		0.5905	0.0030	0.0080	*Robinsoniella(100)*
		81		0.4220	0.0377	0.0450	*Tannerella(100)*
		88		0.5427	0.0364	0.0439	*Limibacter(100)*
		101		0.5452	0.0188	0.0295	*Escherichia/Shigella(100)*
		103		0.4919	0.0033	0.0081	*Turicibacter(100)*
		124		0.5250	0.0218	0.0319	*Meniscus(100)*
		128		0.5630	0.0345	0.0428	*Terasakiella(100)*
		131		0.5449	0.0031	0.0080	*Tannerella(100)*
		137		0.5556	0.0258	0.0349	*Lachnobacterium(100)*
		140		0.6117	0.0022	0.0071	*Hydrogenoanaerobacterium(100)*
		147		0.4437	0.0274	0.0360	*Meniscus(100)*
		197		0.4048	0.0225	0.0322	*Paludibacter(100)*
		201		0.5266	0.0005	0.0026	*Clostridium XlVa(100)*
		235		0.4826	0.0028	0.0080	*Lachnobacterium(100)*
		256		0.4730	0.0023	0.0071	*Meniscus(100)*
		293		0.4278	0.0216	0.0319	*Howardella(100)*
		326		0.4322	0.0031	0.0080	*Robinsoniella(100)*
		329		0.4447	0.0029	0.0080	*Parabacteroides(100)*
		340		0.3957	0.0133	0.0234	*Paludibacter(100)*
		342		0.5087	0.0013	0.0050	*Lactobacillus(100)*
		395		0.4720	0.0055	0.0115	*Syntrophococcus(100)*
		401		0.4189	0.0275	0.0360	*Propionibacterium(100)*
		402		0.4688	0.0017	0.0060	*Meniscus(100)*
		629		0.4569	0.0017	0.0060	*Syntrophococcus(100)*
		790		0.3777	0.0246	0.0341	*Paludibacter(100)*

Table S5: Final linear mixed model results of alpha diversity for each respective time point according to *Fut2* genotype/*secretor* status, or the mouse breeding lineage (founded by *Fut2* $^{-/-}$ or *Fut2* $^{+/+}$ grand dam).

Alpha diversity	Time point	Model Factor	*DF*	*F*-value	*P*-value
Species Richness	TP1	*Intercept*	1,20	204.763	<0.0001
		Fut2	2,20	2.472	0.1098
		Direction	1,8	4.258	0.0730
		Fut2 × Direction	2,20	3.817	0.0394
	TP3	*Intercept*	1,24	79.444	<0.0001
		Direction	1,8	4.032	0.0795
	TP5*	*Intercept*	1,24	260.387	<0.0001
		Direction	1,8	3.119	0.1154
	TP11	Intercept	1,24	151.493	<0.0001
Shannon Entropy	TP1**	*Intercept*	1,24	258.911	<0.0001

	TP3	*Intercept*	1,22	1131.119	<0.0001
		Fut2	2,22	2.889	0.0769
	TP5	*Intercept*	1,24	847.781	<0.0001
	TP11	*Intercept*	1,22	1912.295	<0.0001
		Fut2	2,22	5.300	0.0132
Net Relatedness Index (NRI)	TP1	*Intercept*	1,24	24.167	0.0001
		Direction	1,8	2.656	0.1418
	TP3	*Intercept*	1,23	5.878	0.0236
		Secretor	1,23	3.323	0.0813
	TP5	*Intercept*	1,24	5.109	0.0332
	TP11	*Intercept*	1,24	0.321	0.5765
Nearest Taxon Index (NTI)	TP1	*Intercept*	1,22	52.758	<0.0001
		Secretor	1,22	0.029	0.8672
		Direction	1,8	16.269	0.0038
		Secretor × Direction	1,22	7.260	0.0132
	TP3	*Intercept*	1,24	9.463	0.0052
	TP5	*Intercept*	1,24	9.836	0.0045
		Direction	1,8	3.150	0.1138
	TP11	*Intercept*	1,24	146.348	<0.0001

* $X^{1/2}$ transformed; ** X^3 transformed

Table S6: Final linear mixed model results of alpha diversity metrics for each respective location of the gastrointestinal tract (*Fut2* genotype/*secretor* status, or the mouse breeding lineage (founded by *Fut2* $^{-/-}$ or *Fut2* $^{+/+}$ grand dam)).

Alpha diversity	Tissue location	Model Factors	*DF*	*F*-value	*P*-value
Species Richness	Jejunum	*Intercept*	1,21	102.78	< 0.0001
	Ileum	*Intercept*	1,25	119.744	< 0.0001
		Direction	1,9	10.614	0.0099
	Cecum	*Intercept*	1,27	331.427	< 0.0001
	Colon	*Intercept*	1,26	347.451	< 0.0001
Shannon Entropy	Jejunum	*Intercept*	1,21	1149.763	< 0.0001
	Ileum*	*Intercept*	1,25	175.859	< 0.0001
		Direction	1,9	9.885	0.0119
	Cecum	*Intercept*	1,27	2044.081	< 0.0001
	Colon*	*Intercept*	1,26	224.544	< 0.0001
Net Relatedness Index (NRI)	Jejunum	*Intercept*	1,21	10.116	0.0045
	Ileum	*Intercept*	1,25	32.91	< 0.0001
		Direction	1,9	2.85	0.1257
	Cecum	*Intercept*	1,27	53.195	< 0.0001
	Colon	*Intercept*	1,26	0.571	0.4568
Nearest Taxon Index (NTI)	Jejunum	*Intercept*	1,21	47.35	< 0.0001
		Direction	1,8	3.712	0.0902
	Ileum	*Intercept*	1,25	72.999	< 0.0001
		Direction	1,9	3.55	0.0922
	Cecum	*Intercept*	1,27	14.791	0.0007

	Colon	*Intercept*	1,26	133.855	< 0.0001

* X^2 transformed; **

Table S7: Test for community difference between *Fut2* genotype and breeding direction among fecal time points, based on shared abundance (Bray-Curtis), shared presence (Jaccard), the phylogenetic relatedness (unweighted UniFrac) and distribution of species (Euclidean/Redundancy Analysis).

Time point	Distance	Factors	*F*-value	*P*-value	R^2	adj. R^2
TP1	Bray-Curtis	*Fut2*	0.9279	0.6761	0.1779	0.0311
		Direction	1.8334	0.0016		
		Fut2 × Direction	1.1851	0.0994		
	Jaccard	*Fut2*	0.9559	0.7455	0.1632	0.0137
		Direction	1.4333	0.0014		
		Fut2 × Direction	1.0572	0.1672		
	UniFrac (unweighted)	*Fut2*	0.9624	0.5511	0.1780	0.0312
		Direction	1.6766	0.0096		
		Fut2 × Direction	1.2302	0.0690		
	Redundancy Analysis (Hellinger Distance)	*Fut2*	0.8782	0.6961	0.1932	0.0491
		Direction	2.3231	0.0012		
		Fut2 × Direction	1.3121	0.0856		
TP3	Bray-Curtis	*Fut2*	1.1171	0.1826	0.2014	0.0588
		Direction	2.1688	0.0014		
		Fut2 × Direction	1.3296	0.0378		
	Jaccard	*Fut2*	0.9911	0.5173	0.1625	0.0129
		Direction	1.3665	0.0008		
		Fut2 × Direction	1.0415	0.1794		
	UniFrac (unweighted)	*Fut2*	1.0245	0.3495	0.1673	0.0186
		Direction	1.6726	0.0034		
		Fut2 × Direction	0.9527	0.6123		
	Redundancy Analysis (Hellinger Distance)	*Fut2*	1.0875	0.2839	0.2249	0.0865
		Direction	3.0691	0.0004		
		Fut2 × Direction	1.4402	0.0444		
TP5	Bray-Curtis	*Fut2*	0.8862	0.8084	0.1690	0.0207
		Direction	1.3480	0.0482		
		Fut2 × Direction	1.2879	0.0386		
	Jaccard	*Fut2*	0.9223	0.9742	0.1591	0.0089

		Direction	1.3367	0.0008		
		Fut2 × Direction	1.0576	0.1264		
	UniFrac (unweighted)	*Fut2*	0.8948	0.8666	0.1664	0.0175
		Direction	1.5973	0.0052		
		Fut2 × Direction	1.1003	0.1588		
	Redundancy Analysis (Hellinger Distance)	*Fut2*	0.8369	0.8316	0.1840	0.0383
		Direction	1.8280	0.0070		
		Fut2 × Direction	1.4057	0.0260		
TP11	Bray-Curtis	*Fut2*	1.0359	0.3397	0.1646	0.0155
		Direction	1.4103	0.0352		
		Fut2 × Direction	1.0179	0.3837		
	Jaccard	*Fut2*	1.0459	0.1208	0.1623	0.0128
		Direction	1.3721	0.0004		
		Fut2 × Direction	0.9812	0.6607		
	UniFrac (unweighted)	*Fut2*	1.1230	0.0948	0.1693	0.0209
		Direction	1.5453	0.0012		
		Fut2 × Direction	0.9570	0.6645		
	Redundancy Analysis (Hellinger Distance)	*Fut2*	1.0446	0.3369	0.1628	0.0133
		Direction	1.6299	0.0176		
		Fut2 × Direction	0.8622	0.8094		

Table S8: Test for community difference between *secretor* status and breeding direction among fecal time points, based on shared abundance (Bray-Curtis), shared presence (Jaccard), the phylogenetic relatedness (unweighted UniFrac) and distribution of species (Euclidean/Redundancy Analysis).

Time point	Distance	Factors	*F*-value	*P*-value	R^2	adj. R^2
TP1	Bray-Curtis	*Secretor*	0.8488	0.7950	0.1083	0.0191
		Direction	1.8690	0.0010		
		Secretor × Direction	0.9252	0.6099		
	Jaccard	*Secretor*	0.8785	0.9764	0.0968	0.0065

		Direction	1.4703	0.0004		
		Secretor × Direction	0.8667	0.9870		
	UniFrac (unweighted)	*Secretor*	0.8105	0.8992	0.0984	0.0082
		Direction	1.7282	0.0056		
		Secretor × Direction	0.7341	0.9908		
	Redundancy Analysis (Hellinger Distance)	*Secretor*	0.7436	0.8428	0.1173	0.0290
		Direction	2.3813	0.0018		
		Secretor × Direction	0.8624	0.6465		
TP3	Bray-Curtis	*Secretor*	0.9329	0.5403	0.1218	0.0339
		Direction	2.2361	0.0008		
		Secretor × Direction	0.9906	0.4225		
	Jaccard	*Secretor*	0.9447	0.7964	0.1001	0.0101
		Direction	1.3982	0.0004		
		Secretor × Direction	0.9929	0.4673		
	UniFrac (unweighted)	*Secretor*	0.8562	0.8610	0.1019	0.0121
		Direction	1.7483	0.0028		
		Secretor × Direction	0.7997	0.9570		
	Redundancy Analysis (Hellinger Distance)	*Secretor*	0.9577	0.4535	0.1483	0.0631
		Direction	3.1498	0.0006		
		Secretor × Direction	1.1157	0.2581		
TP5	Bray-Curtis	*Secretor*	0.8680	0.7521	0.0980	0.0078
		Direction	1.3440	0.0592		
		Secretor × Direction	1.0487	0.3231		
	Jaccard	*Secretor*	0.9285	0.8780	0.0980	0.0078
		Direction	1.3350	0.0010		
		Secretor × Direction	0.9975	0.4641		
	UniFrac (unweighted)	*Secretor*	0.8774	0.8142	0.1013	0.0114
		Direction	1.5808	0.0046		
		Secretor × Direction	0.9238	0.6789		
	Redundancy Analysis	*Secretor*	0.8543	0.6901	0.1109	0.0220
		Direction	1.7999	0.0100		

	(Hellinger Distance)	*Secretor* × Direction	1.0886	0.2929		
TP11	Bray-Curtis	*Secretor*	0.9515	0.5281	0.0993	0.0093
		Direction	1.4453	0.0282		
		Secretor × Direction	0.9117	0.6361		
	Jaccard	*Secretor*	1.0361	0.2312	0.1007	0.0108
		Direction	1.4030	0.0002		
		Secretor × Direction	0.9198	0.9562		
	UniFrac (unweighted)	*Secretor*	1.0302	0.3397	0.1045	0.0149
		Direction	1.5640	0.0014		
		Secretor × Direction	0.9054	0.7846		
	Redundancy Analysis (Hellinger Distance)	*Secretor*	0.9943	0.4325	0.1019	0.0121
		Direction	1.6835	0.0124		
		Secretor × Direction	0.7261	0.9522		

Table S9: Community differences of mucosal associated microbial communities between genotypes and breeding directions, based on shared abundance (Bray-Curtis), shared presence (Jaccard), the phylogenetic relatedness (unweighted UniFrac) and distribution of species (Euclidean/Redundancy Analysis).

Tissue location	Distance	Factors	*F*-values	*P*-values	R^2	adj. R^2
Jejunum	Bray-Curtis	*Fut2*	0.9891	0.4715	0.1660	-0.0008
		Direction	1.1465	0.1878		
		Fut2 × Direction	0.9259	0.6559		
	Jaccard	*Fut2*	1.0094	0.3965	0.1715	0.0058
		Direction	1.2782	0.0456		
		Fut2 × Direction	0.9384	0.7237		
	UniFrac (unweighted)	*Fut2*	0.9386	0.6207	0.1754	0.0105
		Direction	1.4409	0.0436		
		Fut2 × Direction	0.9994	0.4473		
	Redundancy	*Fut2*	0.9740	0.4939	0.1645	-0.0026

	Analysis (Hellinger Distance)	Direction	1.3600	0.1016		
		Fut2 × Direction	0.8066	0.8332		
Ileum	Bray-Curtis	*Fut2*	1.2114	0.1942	0.2004	0.0671
		Direction	2.8928	0.0050		
		Fut2 × Direction	1.1012	0.2855		
	Jaccard	*Fut2*	0.9733	0.5857	0.1550	0.0142
		Direction	1.6254	0.0004		
		Fut2 × Direction	0.9665	0.6269		
	UniFrac (unweighted)	*Fut2*	0.9631	0.5345	0.1651	0.0260
		Direction	1.8576	0.0054		
		Fut2 × Direction	1.0751	0.2660		
	Redundancy Analysis (Hellinger Distance)	*Fut2*	1.4972	0.1132	0.2604	0.1371
		Direction	4.7254	0.0022		
		Fut2 × Direction	1.4212	0.1496		
Cecum	Bray-Curtis	*Fut2*	1.1182	0.1152	0.1450	0.0114
		Direction	1.3637	0.0184		
		Fut2 × Direction	0.9133	0.8010		
	Jaccard	*Fut2*	0.9844	0.6607	0.1440	0.0103
		Direction	1.4326	0.0002		
		Fut2 × Direction	0.9912	0.5843		
	UniFrac (unweighted)	*Fut2*	0.9269	0.8702	0.1430	0.0090
		Direction	1.5509	0.0002		
		Fut2 × Direction	0.9664	0.6713		
	Redundancy Analysis (Hellinger Distance)	*Fut2*	1.2213	0.0610	0.1536	0.0214
		Direction	1.5877	0.0064		
		Fut2 × Direction	0.8893	0.8054		
Colon	Bray-Curtis	*Fut2*	0.9427	0.6011	0.1383	-0.0007
		Direction	1.3628	0.0662		
		Fut2 × Direction	0.8632	0.8204		
	Jaccard	*Fut2*	0.9552	0.7832	0.1515	0.0147
		Direction	1.6381	0.0002		

		Fut2 × Direction	0.9936	0.5187		
	UniFrac (unweighted)	*Fut2*	0.9428	0.7307	0.1524	0.0156
		Direction	1.7274	0.0002		
		Fut2 × Direction	0.9795	0.5577		
	Redundancy Analysis (Hellinger Distance)	*Fut2*	0.8818	0.6939	0.1391	0.0002
		Direction	1.5029	0.0626		
		Fut2 × Direction	0.8710	0.7079		

Table S10: Community differences of mucosal associated microbial communities between *secretor* status and breeding directions, based on shared abundance (Bray-Curtis), shared presence (Jaccard), the phylogenetic relatedness (unweighted UniFrac) and distribution of species (Euclidean/Redundancy Analysis).

Tissue location	Distance	Factors	*F*-values	*P*-values	R^2	adj. R^2
Jejunum	Bray-Curtis	*Secretor*	0.9260	0.5877	0.0913	-0.0097
		Direction	1.1132	0.2354		
		Secretor × Direction	0.6726	0.9884		
	Jaccard	*Secretor*	1.1069	0.1630	0.1068	0.0076
		Direction	1.3043	0.0388		
		Secretor × Direction	0.8176	0.9652		
	UniFrac (unweighted)	*Secretor*	1.0516	0.3021	0.1074	0.0082
		Direction	1.4723	0.0360		
		Secretor × Direction	0.7234	0.9748		
	Redundancy Analysis (Hellinger Distance)	*Secretor*	0.9583	0.4629	0.0927	-0.0081
		Direction	1.3228	0.1160		
		Secretor × Direction	0.4778	0.9992		
Ileum	Bray-Curtis	*Secretor*	0.9557	0.4219	0.1264	0.0445
		Direction	2.7056	0.0050		
		Secretor × Direction	0.9684	0.4003		
	Jaccard	*Secretor*	0.9622	0.5779	0.1025	0.0183

		Direction	1.6137	0.0006		
		Secretor × Direction	1.0779	0.2230		
	UniFrac (unweighted)	*Secretor*	1.0622	0.3005	0.1190	0.0365
		Direction	1.8622	0.0046		
		Secretor × Direction	1.3998	0.0552		
	Redundancy Analysis (Hellinger Distance)	*Secretor*	1.2447	0.2110	0.1710	0.0933
		Direction	4.2789	0.0030		
		Secretor × Direction	1.0766	0.2859		
Cecum	Bray-Curtis	*Secretor*	1.2660	0.0468	0.0943	0.0144
		Direction	1.3794	0.0152		
		Secretor × Direction	0.8965	0.7573		
	Jaccard	*Secretor*	0.9783	0.6397	0.0908	0.0106
		Direction	1.4516	0.0002		
		Secretor × Direction	0.9646	0.7648		
	UniFrac (unweighted)	*Secretor*	0.8337	0.9814	0.0879	0.0074
		Direction	1.5740	0.0002		
		Secretor × Direction	0.8697	0.9290		
	Redundancy Analysis (Hellinger Distance)	*Secretor*	1.4601	0.0192	0.1023	0.0231
		Direction	1.6199	0.0074		
		Secretor × Direction	0.7958	0.8872		
Colon	Bray-Curtis	*Secretor*	0.9245	0.5463	0.0826	-0.0008
		Direction	1.3595	0.0676		
		Secretor × Direction	0.6856	0.9894		
	Jaccard	*Secretor*	0.9898	0.5067	0.0966	0.0144
		Direction	1.6437	0.0002		
		Secretor × Direction	0.8942	0.9262		
	UniFrac (unweighted)	*Secretor*	0.9798	0.5281	0.0979	0.0159
		Direction	1.7406	0.0004		
		Secretor × Direction	0.8615	0.8638		
	Redundancy Analysis	*Secretor*	0.8724	0.6233	0.0825	-0.0009
		Direction	1.5030	0.0628		

(Hellinger Distance)	*Secretor* × Direction	0.5919	0.9890		

Table S11: Importance of single genera on relative network stability as the mean relative change of the repsect metric along the removal sequcene. Taxa marked grey indicate taxa with repeated occurrence in another metric or time point (indicator genera for # secretor, ## non-secretor status, † *Fut2*$^{+/+}$ dam, †† *Fut2*$^{-/-}$ dam).

Metric	Time point	Taxonomy (RDP 9)	Importance
Size biggest component	TP1	*Pseudoflavonifractor*	0.0200
		Enterorhabdus	0.0195
		Parasutterella	0.0191
		Odoribacter	0.0119
		Alistipes	0.0116
		Clostridiales uncl.	0.0107
		Anaerovorax	0.0106
		Limibacter	0.0106
		Deltaproteobacteria uncl.	0.0105
		Coprobacillus	0.0104
	TP3	*Leuconostoc*	0.0867
		Bacilli uncl.	0.0454
		Lachnospiracea incertae sedis	0.0383
		Enterorhabdus	0.0341
		Alphaproteobacteria uncl.	0.0334
		Mucispirillum	0.0324
		Sutterellaceae uncl.	0.0277
		Segetibacter	0.0265
		Deltaproteobacteria uncl.	0.0257
		Parabacteroides	0.0245
	TP5	*Lachnospiraceae uncl.* †	0.0627
		Phycisphaera	0.0428
		Limibacter	0.0398
		Pantoea	0.0385
		Deltaproteobacteria uncl.	0.0380
		Halomonas	0.0375
		Butyricimonas	0.0329
		Streptophyta	0.0317
		Desulfovibrionaceae uncl.	0.0303
		Syntrophococcus	0.0299
	TP11	*Burkholderiales incertae sedis uncl.*	0.1418
		Alphaproteobacteria uncl.	0.1332
		Clostridia uncl.	0.1177
		Lachnospiracea incertae sedis	0.1033
		Ruminococcus ##	0.0945
		Syntrophococcus	0.0933
		Paraprevotella	0.0920
		Legionella	0.0865
		Prevotellaceae uncl. ††	0.0734
		Gemella	0.0701
Closeness	TP1	*Pseudoflavonifractor*	0.4551
		Enterorhabdus	0.4210
		Parasutterella	0.4152
		Robinsoniella	0.1478
		Anaerophaga	0.1292
		Erysipelotrichaceae incertae sedis	0.0994
		Alistipes	0.0919
		Odoribacter	0.0847

		Desulfocurvus	0.0711
		Deltaproteobacteria uncl.	0.0663
	TP3	*Leuconostoc*	0.5658
		Bacilli uncl.	0.4865
		Lachnospiracea incertae sedis	0.4036
		Enterorhabdus	0.3410
		Alphaproteobacteria uncl.	0.3325
		Mucispirillum	0.3205
		Sutterellaceae uncl.	0.3117
		Gemella	0.2968
		Parabacteroides	0.2284
		Streptococcus	0.2178
	TP5	*Lachnospiraceae uncl.* †	0.4466
		Limibacter	0.3457
		Halomonas	0.3285
		Desulfovibrionaceae uncl.	0.2974
		Deltaproteobacteria uncl.	0.2901
		Pantoea	0.2882
		Phycisphaera	0.2548
		Butyricimonas	0.2407
		Duganella	0.2164
		Asaccharobacter	0.2152
	TP11	*Burkholderiales incertae sedis uncl.*	0.3209
		Alphaproteobacteria uncl.	0.3172
		Ruminococcus ##	0.2960
		Clostridia uncl.	0.2523
		Syntrophococcus	0.2517
		Prevotellaceae uncl. ††	0.2513
		Lachnospiracea incertae sedis	0.2386
		Paraprevotella	0.1974
		Streptophyta	0.1925
		Phycisphaera	0.1731
Diameter	TP1	*Clostridium IV*	0.1585
		Odoribacter	0.0667
		Parasutterella	0.0633
		Anaerophaga	0.0616
		Lactobacillus	0.0495
		Lachnospiraceae uncl. †	0.0351
		Bacteroidales uncl.	0.0314
		Prevotella ††	0.0250
		Ruminococcus ##	0.0208
		Alistipes	0.0152
	TP3	*Parasutterella* ††	0.1785
		Clostridiales uncl.	0.1424
		Ruminococcus ##	0.1017
		Bacteroidales uncl.	0.0823
		Sphingobacteriales uncl.	0.0812
		Porphyromonadaceae uncl.	0.0801
		Asaccharobacter	0.0738
		Lactobacillaceae uncl.	0.0685
		Lachnospiraceae uncl. †	0.0646
		Clostridium IV	0.0550
	TP5	*Butyricimonas*	0.1229
		TM7 genus incertae sedis	0.1142
		Lactococcus	0.1065

		Collimonas	0.0966
		Lachnospiraceae uncl. †	0.0928
		Desulfovibrionaceae uncl.	0.0876
		Barnesiella †	0.0830
		Clostridium XI	0.0741
		Butyricicoccus	0.0660
		Lactobacillus	0.0630
	TP11	*Clostridia uncl.*	0.1646
		Lachnospiracea incertae sedis	0.1591
		Plantibacter	0.1503
		Parabacteroides	0.1431
		Enterobacteriaceae uncl.	0.1348
		Parasutterella	0.1288
		Turicibacter	0.1142
		Clostridium XlVa	0.1064
		Ruminococcus ##	0.0910
		Citrobacter	0.0890
Number of components	TP1	*Pseudoflavonifractor*	0.8105
		Parasutterella	0.7979
		Enterorhabdus	0.7951
		Odoribacter	0.1271
		Alistipes	0.1044
		Robinsoniella	0.0658
		Anaerophaga	0.0622
		Erysipelotrichaceae incertae sedis	0.0482
		Clostridiales uncl.	0.0316
		Anaerovorax	0.0164
	TP3	*Leuconostoc*	1.1339
		Alphaproteobacteria uncl.	0.8712
		Lachnospiracea incertae sedis	0.8676
		Bacilli uncl.	0.8641
		Enterorhabdus	0.8461
		Sutterellaceae uncl.	0.7715
		Bilophila	0.4282
		Segetibacter	0.4118
		Mucispirillum	0.4111
		Byssovorax	0.4054
	TP5	*Pantoea*	0.8197
		Limibacter	0.8138
		Lachnospiraceae uncl. †	0.7949
		Phycisphaera	0.4998
		Butyricimonas	0.4683
		Deltaproteobacteria uncl.	0.4508
		Duganella	0.4371
		Streptophyta	0.4333
		Halomonas	0.4328
		Gemella	0.4322
	TP11	*Streptophyta*	0.5103
		Prevotellaceae uncl. ††	0.4800
		Alphaproteobacteria uncl.	0.3792
		Syntrophococcus	0.3466
		Gemella	0.3216
		Marvinbryantia	0.3043
		Burkholderiales incertae sedis uncl.	0.2732
		Lachnospiracea incertae sedis	0.2605

		Paraprevotella	0.1992
		Ruminococcus ##	0.1882
Transitivity	TP1	*Alistipes*	0.0690
		Odoribacter	0.0465
		Lachnospiraceae uncl. †	0.0454
		Proteobacteria uncl.	0.0429
		Parabacteroides	0.0426
		Alphaproteobacteria uncl.	0.0396
		Bacteroidales uncl.	0.0377
		Porphyromonadaceae uncl.	0.0354
		Clostridiales uncl.	0.0348
		Bacteria uncl.	0.0330
	TP3	*Lachnospiraceae uncl.* †	0.1119
		Prevotella ††	0.1069
		Prevotellaceae uncl. ††	0.1027
		Escherichia/Shigella ††	0.0989
		Ruminococcaceae uncl.	0.0911
		Clostridium XlVa	0.0863
		Bacteroides †	0.0680
		Alistipes	0.0670
		Clostridiales uncl.	0.0667
		Coriobacteriaceae uncl.	0.0645
	TP5	*Parabacteroides*	0.1674
		Oscillibacter	0.1274
		Alistipes	0.1137
		Ruminococcaceae uncl.	0.1038
		Lactobacillus	0.1008
		Clostridium XI	0.0916
		Bacteroidales uncl.	0.0912
		Lachnospiraceae uncl. †	0.0834
		Bacteria uncl.	0.0820
		Syntrophococcus	0.0779
	TP11	*Lachnospiraceae uncl.* †	0.3077
		Alistipes	0.2179
		Clostridiales uncl.	0.1793
		Ruminococcaceae uncl.	0.1745
		Oscillibacter	0.1592
		Firmicutes uncl.	0.1438
		Bacteria uncl.	0.1338
		Lactobacillales uncl.	0.1103
		Lachnospiracea incertae sedis	0.0922
		Alphaproteobacteria uncl.	0.0894

Table S12: Importance of single OTUs on relative network stability as the mean relative change of the repsect metric along the removal sequcene. Taxa marked grey indicate taxa with repeated occurrence in another metric or time point (indicator OTUs for † *Fut2*$^{+/+}$ dam, †† *Fut2*$^{-/-}$ dam).

Metric	Time points	OTU	Importance	OTU Taxonomy (RDP 9)
Size biggest component	TP1	12	0.0807	*Bacteroidetes;Bacteroidia;Bacteroidales;Bacteroidaceae;Bacteroides;*
		270	0.0663	*Bacteroidetes;Bacteroidia;Bacteroidales;Rikenellaceae;Rikenella;*
		91	0.0625	*Bacteroidetes;Bacteroidia;Bacteroidales;Rikenellaceae;Alistipes;*
		24	0.0548	*Bacteroidetes;Bacteroidia;Bacteroidales;Rikenellaceae;Rikenella;*
		6142	0.0500	*Firmicutes;Clostridia;Clostridiales;Lachnospiraceae;Roseburia;*
		5 †	0.0486	*Proteobacteria;Epsilonproteobacteria;Campylobacterales;Helicobacteraceae;Helicobacter;*
		599	0.0474	*Firmicutes;Clostridia;Clostridiales;Ruminococcaceae;Pseudoflavonifractor;*
		9	0.0405	*Bacteroidetes;Bacteroidia;Bacteroidales;Rikenellaceae;Rikenella;*
		1068	0.0400	*Bacteroidetes;Bacteroidia;Bacteroidales;Rikenellaceae;Alistipes;*
		20	0.0393	*Deferribacteres;Deferribacteres;Deferribacterales;Deferribacteraceae;Mucispirillum;*
	TP3	35	0.1531	*Firmicutes;Clostridia;Clostridiales;Lachnospiraceae;Robinsoniella;*
		25 †	0.1069	*Bacteroidetes;Bacteroidia;Bacteroidales;Porphyromonadaceae;Paludibacter;*
		51 †	0.1045	*Bacteroidetes;Bacteroidia;Bacteroidales;Porphyromonadaceae;Paludibacter;*
		6082	0.0922	*Firmicutes;Clostridia;Clostridiales;Ruminococcaceae;Acetanaerobacterium;*
		196	0.0780	*Firmicutes;Clostridia;Clostridiales;Lachnospiraceae;Robinsoniella;*
		160 †	0.0747	*Bacteroidetes;Bacteroidia;Bacteroidales;Porphyromonadaceae;Paludibacter;*
		48	0.0744	*Firmicutes;Clostridia;Clostridiales;Lachnospiraceae;Robinsoniella;*
		781	0.0698	*Firmicutes;Clostridia;Clostridiales;Lachnospiraceae;Clostridium XlVb;*
		11 †	0.0664	*Bacteroidetes;Bacteroidia;Bacteroidales;Marinilabiaceae;Anaerophaga;*
		74	0.0655	*Bacteroidetes;Bacteroidia;Bacteroidales;Porphyromonadaceae;Odoribacter;*
	TP5	146	0.2214	*Bacteroidetes;Bacteroidia;Bacteroidales;Porphyromonadaceae;Paludibacter;*
		248	0.1775	*Firmicutes;Clostridia;Clostridiales;Lachnospiraceae;Robinsoniella;*
		260	0.1467	*Firmicutes;Clostridia;Clostridiales;Ruminococcaceae;Oscillibacter;*
		426	0.1436	*Firmicutes;Clostridia;Clostridiales;Ruminococcaceae;Pseudoflavonifractor;*
		120	0.1409	*Bacteroidetes;Bacteroidia;Bacteroidales;Porphyromonadaceae;Paludibacter;*
		9	0.1384	*Bacteroidetes;Bacteroidia;Bacteroidales;Rikenellaceae;Rikenella;*
		671	0.1304	*Bacteroidetes;Bacteroidia;Bacteroidales;Porphyromonadaceae;Paludibacter;*
		354	0.1282	*Bacteroidetes;Bacteroidia;Bacteroidales;Prevotellaceae;Paraprevotella;*
		37	0.1177	*Bacteroidetes;Bacteroidia;Bacteroidales;Porphyromonadaceae;Paludibacter;*
		118	0.1139	*Bacteroidetes;Bacteroidia;Bacteroidales;Porphyromonadaceae;Paludibacter;*

	TP11	161	0.3070	*Proteobacteria;Epsilonproteobacteria;Campylobacterales;Helicobacteraceae;Helicobacter;*
		76	0.2905	*Bacteroidetes;Bacteroidia;Bacteroidales;Porphyromonadaceae;Paludibacter;*
		195	0.2354	*Bacteroidetes;Bacteroidia;Bacteroidales;Marinilabiaceae;Alkaliflexus;*
		6836	0.2100	*Firmicutes;Clostridia;Clostridiales;Ruminococcaceae;Clostridium IV;*
		412 †	0.1926	*Firmicutes;Clostridia;Clostridiales;Ruminococcaceae;Oscillibacter;*
		957	0.1903	*Proteobacteria;Deltaproteobacteria;Desulfovibrionales;Desulfovibrionaceae;Desulfocurvus;*
		120	0.1704	*Bacteroidetes;Bacteroidia;Bacteroidales;Porphyromonadaceae;Paludibacter;*
		19 ††	0.1641	*Bacteroidetes;Bacteroidia;Bacteroidales;Porphyromonadaceae;Tannerella;*
		457	0.1509	*Bacteroidetes;Bacteroidia;Bacteroidales;Rikenellaceae;Rikenella;*
		7 ††	0.1341	*Bacteroidetes;Sphingobacteria;Sphingobacteriales;Cytophagaceae;Meniscus;*
Closeness	TP1	12	0.0818	*Bacteroidetes;Bacteroidia;Bacteroidales;Bacteroidaceae;Bacteroides;*
		270	0.0669	*Bacteroidetes;Bacteroidia;Bacteroidales;Rikenellaceae;Rikenella;*
		91	0.0621	*Bacteroidetes;Bacteroidia;Bacteroidales;Rikenellaceae;Alistipes;*
		24	0.0541	*Bacteroidetes;Bacteroidia;Bacteroidales;Rikenellaceae;Rikenella;*
		6142	0.0505	*Firmicutes;Clostridia;Clostridiales;Lachnospiraceae;Roseburia;*
		5 †	0.0477	*Proteobacteria;Epsilonproteobacteria;Campylobacterales;Helicobacteraceae;Helicobacter;*
		599	0.0467	*Firmicutes;Clostridia;Clostridiales;Ruminococcaceae;Pseudoflavonifractor;*
		1068	0.0396	*Bacteroidetes;Bacteroidia;Bacteroidales;Rikenellaceae;Alistipes;*
		9	0.0395	*Bacteroidetes;Bacteroidia;Bacteroidales;Rikenellaceae;Rikenella;*
		20	0.0394	*Deferribacteres;Deferribacteres;Deferribacterales;Deferribacteraceae;Mucispirillum;*
	TP3	35	0.1608	*Firmicutes;Clostridia;Clostridiales;Lachnospiraceae;Robinsoniella;*
		25 †	0.1114	*Bacteroidetes;Bacteroidia;Bacteroidales;Porphyromonadaceae;Paludibacter;*
		51 †	0.1091	*Bacteroidetes;Bacteroidia;Bacteroidales;Porphyromonadaceae;Paludibacter;*
		6082	0.0960	*Firmicutes;Clostridia;Clostridiales;Ruminococcaceae;Acetanaerobacterium;*
		196	0.0798	*Firmicutes;Clostridia;Clostridiales;Lachnospiraceae;Robinsoniella;*
		48	0.0793	*Firmicutes;Clostridia;Clostridiales;Lachnospiraceae;Robinsoniella;*
		160 †	0.0760	*Bacteroidetes;Bacteroidia;Bacteroidales;Porphyromonadaceae;Paludibacter;*
		781	0.0726	*Firmicutes;Clostridia;Clostridiales;Lachnospiraceae;Clostridium XlVb;*
		11 †	0.0702	*Bacteroidetes;Bacteroidia;Bacteroidales;Marinilabiaceae;Anaerophaga;*
		74	0.0692	*Bacteroidetes;Bacteroidia;Bacteroidales;Porphyromonadaceae;Odoribacter;*
	TP5	146	0.2442	*Bacteroidetes;Bacteroidia;Bacteroidales;Porphyromonadaceae;Paludibacter;*
		248	0.1946	*Firmicutes;Clostridia;Clostridiales;Lachnospiraceae;Robinsoniella;*
		260	0.1616	*Firmicutes;Clostridia;Clostridiales;Ruminococcaceae;Oscillibacter;*
		426	0.1560	*Firmicutes;Clostridia;Clostridiales;Ruminococcaceae;Pseudoflavonifractor;*

		120	0.1555	*Bacteroidetes;Bacteroidia;Bacteroidales;Porphyromonadaceae;Paludibacter;*
		9	0.1531	*Bacteroidetes;Bacteroidia;Bacteroidales;Rikenellaceae;Rikenella;*
		671	0.1426	*Bacteroidetes;Bacteroidia;Bacteroidales;Porphyromonadaceae;Paludibacter;*
		354	0.1418	*Bacteroidetes;Bacteroidia;Bacteroidales;Prevotellaceae;Paraprevotella;*
		37	0.1300	*Bacteroidetes;Bacteroidia;Bacteroidales;Porphyromonadaceae;Paludibacter;*
		118	0.1258	*Bacteroidetes;Bacteroidia;Bacteroidales;Porphyromonadaceae;Paludibacter;*
	TP11	161	0.3424	*Proteobacteria;Epsilonproteobacteria;Campylobacterales;Helicobacteraceae;Helicobacter;*
		76	0.3236	*Bacteroidetes;Bacteroidia;Bacteroidales;Porphyromonadaceae;Paludibacter;*
		195	0.2613	*Bacteroidetes;Bacteroidia;Bacteroidales;Marinilabiaceae;Alkaliflexus;*
		6836	0.2323	*Firmicutes;Clostridia;Clostridiales;Ruminococcaceae;Clostridium IV;*
		412 †	0.2131	*Firmicutes;Clostridia;Clostridiales;Ruminococcaceae;Oscillibacter;*
		957	0.2104	*Proteobacteria;Deltaproteobacteria;Desulfovibrionales;Desulfovibrionaceae;Desulfocurvus;*
		120	0.1869	*Bacteroidetes;Bacteroidia;Bacteroidales;Porphyromonadaceae;Paludibacter;*
		19 ††	0.1809	*Bacteroidetes;Bacteroidia;Bacteroidales;Porphyromonadaceae;Tannerella;*
		457	0.1684	*Bacteroidetes;Bacteroidia;Bacteroidales;Rikenellaceae;Rikenella;*
		7 ††	0.1516	*Bacteroidetes;Sphingobacteria;Sphingobacteriales;Cytophagaceae;Meniscus;*
Diameter	TP1	2126	0.0517	*Bacteroidetes;Bacteroidia;Bacteroidales;Porphyromonadaceae;Barnesiella;*
		1485	0.0495	*Bacteroidetes;Bacteroidia;Bacteroidales;Porphyromonadaceae;Paludibacter;*
		20	0.0448	*Deferribacteres;Deferribacteres;Deferribacterales;Deferribacteraceae;Mucispirillum;*
		300	0.0443	*Bacteroidetes;Bacteroidia;Bacteroidales;Porphyromonadaceae;Paludibacter;*
		5258	0.0436	*Firmicutes;Clostridia;Clostridiales;Lachnospiraceae;Robinsoniella;*
		5738	0.0408	*Bacteroidetes;Bacteroidia;Bacteroidales;Porphyromonadaceae;Paludibacter;*
		1546	0.0395	*Bacteroidetes;Bacteroidia;Bacteroidales;Rikenellaceae;Alistipes;*
		304	0.0383	*Bacteroidetes;Bacteroidia;Bacteroidales;Porphyromonadaceae;Parabacteroides;*
		5 †	0.0364	*Proteobacteria;Epsilonproteobacteria;Campylobacterales;Helicobacteraceae;Helicobacter;*
		1307	0.0358	*Bacteroidetes;Bacteroidia;Bacteroidales;Rikenellaceae;Alistipes;*
	TP3	35	0.1903	*Firmicutes;Clostridia;Clostridiales;Lachnospiraceae;Robinsoniella;*
		781	0.0879	*Firmicutes;Clostridia;Clostridiales;Lachnospiraceae;Clostridium XlVb;*
		5125	0.0834	*Firmicutes;Clostridia;Clostridiales;Clostridiaceae 1;Anaerobacter;*
		1249	0.0799	*Firmicutes;Clostridia;Clostridiales;Lachnospiraceae;Lachnobacterium;*
		49	0.0751	*Firmicutes;Clostridia;Clostridiales;Clostridiaceae 1;Anaerobacter;*
		74	0.0739	*Bacteroidetes;Bacteroidia;Bacteroidales;Porphyromonadaceae;Odoribacter;*
		1809	0.0735	*Tenericutes;Mollicutes;Anaeroplasmatales;Anaeroplasmataceae;Anaeroplasma;*
		23	0.0733	*Bacteroidetes;Bacteroidia;Bacteroidales;Porphyromonadaceae;Barnesiella;*
		3 ††	0.0720	*Firmicutes;Negativicutes;Selenomonadales;Veillonellaceae;Anaerospora;*

		48	0.0716	*Firmicutes;Clostridia;Clostridiales;Lachnospiraceae;Robinsoniella;*
	TP5	146	0.1802	*Bacteroidetes;Bacteroidia;Bacteroidales;Porphyromonadaceae;Paludibacter;*
		120	0.1535	*Bacteroidetes;Bacteroidia;Bacteroidales;Porphyromonadaceae;Paludibacter;*
		354	0.1483	*Bacteroidetes;Bacteroidia;Bacteroidales;Prevotellaceae;Paraprevotella;*
		9	0.1341	*Bacteroidetes;Bacteroidia;Bacteroidales;Rikenellaceae;Rikenella;*
		179	0.1243	*Bacteroidetes;Bacteroidia;Bacteroidales;Porphyromonadaceae;Paludibacter;*
		37	0.1151	*Bacteroidetes;Bacteroidia;Bacteroidales;Porphyromonadaceae;Paludibacter;*
		260	0.1106	*Firmicutes;Clostridia;Clostridiales;Ruminococcaceae;Oscillibacter;*
		671	0.1086	*Bacteroidetes;Bacteroidia;Bacteroidales;Porphyromonadaceae;Paludibacter;*
		248	0.1045	*Firmicutes;Clostridia;Clostridiales;Lachnospiraceae;Robinsoniella;*
		383	0.0997	*Firmicutes;Clostridia;Clostridiales;Lachnospiraceae;Robinsoniella;*
	TP11	161	0.2041	*Proteobacteria;Epsilonproteobacteria;Campylobacterales;Helicobacteraceae;Helicobacter;*
		76	0.2019	*Bacteroidetes;Bacteroidia;Bacteroidales;Porphyromonadaceae;Paludibacter;*
		957	0.1106	*Proteobacteria;Deltaproteobacteria;Desulfovibrionales;Desulfovibrionaceae;Desulfocurvus;*
		19 ††	0.1018	*Bacteroidetes;Bacteroidia;Bacteroidales;Porphyromonadaceae;Tannerella;*
		6836	0.1013	*Firmicutes;Clostridia;Clostridiales;Ruminococcaceae;Clostridium IV;*
		195	0.1010	*Bacteroidetes;Bacteroidia;Bacteroidales;Marinilabiaceae;Alkaliflexus;*
		412 †	0.1002	*Firmicutes;Clostridia;Clostridiales;Ruminococcaceae;Oscillibacter;*
		7 ††	0.0931	*Bacteroidetes;Sphingobacteria;Sphingobacteriales;Cytophagaceae;Meniscus;*
		457	0.0916	*Bacteroidetes;Bacteroidia;Bacteroidales;Rikenellaceae;Rikenella;*
		114	0.0722	*Bacteroidetes;Bacteroidia;Bacteroidales;Porphyromonadaceae;Paludibacter;*
Number of components	TP1	512	0.0123	*Firmicutes;Clostridia;Clostridiales;Lachnospiraceae;Robinsoniella;*
		5007	0.0119	*Firmicutes;Clostridia;Clostridiales;Lachnospiraceae;Robinsoniella;*
		136	0.0091	*Bacteroidetes;Bacteroidia;Bacteroidales;Porphyromonadaceae;Paludibacter;*
		7476	0.0091	*Firmicutes;Clostridia;Clostridiales;Clostridiaceae 4;Thermotalea;*
		2126	0.0090	*Bacteroidetes;Bacteroidia;Bacteroidales;Porphyromonadaceae;Barnesiella;*
		91	0.0090	*Bacteroidetes;Bacteroidia;Bacteroidales;Rikenellaceae;Alistipes;*
		270	0.0087	*Bacteroidetes;Bacteroidia;Bacteroidales;Rikenellaceae;Rikenella;*
		1286	0.0087	*Proteobacteria;Epsilonproteobacteria;Campylobacterales;Helicobacteraceae;Helicobacter;*
		133	0.0087	*Proteobacteria;Betaproteobacteria;Burkholderiales;Sutterellaceae;Parasutterella;*
		1134	0.0087	*Firmicutes;Clostridia;Clostridiales;Ruminococcaceae;Papillibacter;*
	TP3	896	0.0162	*Bacteroidetes;Bacteroidia;Bacteroidales;Rikenellaceae;Rikenella;*
		2714	0.0145	*Firmicutes;Clostridia;Clostridiales;Lachnospiraceae;Lactonifactor;*
		1638	0.0143	*Firmicutes;Clostridia;Clostridiales;Lachnospiraceae;Roseburia;*
		215	0.0132	*Proteobacteria;Alphaproteobacteria;Rhizobiales;Beijerinckiaceae;Methylovirgula;*

		167	0.0123	*Bacteroidetes;Bacteroidia;Bacteroidales;Porphyromonadaceae;Paludibacter;*
		35	0.0116	*Firmicutes;Clostridia;Clostridiales;Lachnospiraceae;Robinsoniella;*
		2063	0.0115	*Bacteroidetes;Bacteroidia;Bacteroidales;Porphyromonadaceae;Paludibacter;*
		51 †	0.0109	*Bacteroidetes;Bacteroidia;Bacteroidales;Porphyromonadaceae;Paludibacter;*
		25 †	0.0106	*Bacteroidetes;Bacteroidia;Bacteroidales;Porphyromonadaceae;Paludibacter;*
		135 ††	0.0093	*Proteobacteria;Gammaproteobacteria;Enterobacteriales;Enterobacteriaceae;Escherichia/Shigella;*
	TP5	27	0.0121	*Bacteroidetes;Bacteroidia;Bacteroidales;Porphyromonadaceae;Paludibacter;*
		248	0.0115	*Firmicutes;Clostridia;Clostridiales;Lachnospiraceae;Robinsoniella;*
		112	0.0100	*Firmicutes;Clostridia;Clostridiales;Lachnospiraceae;Robinsoniella;*
		18	0.0095	*Bacteroidetes;Bacteroidia;Bacteroidales;Porphyromonadaceae;Butyricimonas;*
		3853	0.0093	*Firmicutes;Clostridia;Clostridiales;Lachnospiraceae;Robinsoniella;*
		900	0.0092	*Bacteroidetes;Bacteroidia;Bacteroidales;Porphyromonadaceae;Barnesiella;*
		171	0.0092	*Firmicutes;Clostridia;Clostridiales;Lachnospiraceae;Robinsoniella;*
		375 †	0.0092	*Bacteroidetes;Bacteroidia;Bacteroidales;Rikenellaceae;Alistipes;*
		3505	0.0092	*Bacteroidetes;Bacteroidia;Bacteroidales;Rikenellaceae;Alistipes;*
		1277	0.0091	*Bacteroidetes;Bacteroidia;Bacteroidales;Porphyromonadaceae;Paludibacter;*
	TP11	268 ††	0.0176	*Bacteroidetes;Bacteroidia;Bacteroidales;Porphyromonadaceae;Paludibacter;*
		343	0.0171	*Bacteroidetes;Bacteroidia;Bacteroidales;Marinilabiaceae;Anaerophaga;*
		4 †	0.0168	*Bacteroidetes;Sphingobacteria;Sphingobacteriales;Cytophagaceae;Meniscus;*
		29 ††	0.0143	*Bacteroidetes;Bacteroidia;Bacteroidales;Bacteroidaceae;Bacteroides;*
		33	0.0136	*Tenericutes;Mollicutes;Anaeroplasmatales;Anaeroplasmataceae;Anaeroplasma;*
		19 ††	0.0118	*Bacteroidetes;Bacteroidia;Bacteroidales;Porphyromonadaceae;Tannerella;*
		7 ††	0.0101	*Bacteroidetes;Sphingobacteria;Sphingobacteriales;Cytophagaceae;Meniscus;*
		692	0.0101	*Firmicutes;Clostridia;Clostridiales;Lachnospiraceae;Roseburia;*
		1741	0.0100	*Firmicutes;Clostridia;Clostridiales;Ruminococcaceae;Oscillibacter;*
		331	0.0099	*Bacteroidetes;Bacteroidia;Bacteroidales;Marinilabiaceae;Anaerophaga;*
Transitivity	TP1	29 ††	0.1289	*Bacteroidetes;Bacteroidia;Bacteroidales;Bacteroidaceae;Bacteroides;*
		36 ††	0.0977	*Bacteroidetes;Bacteroidia;Bacteroidales;Rikenellaceae;Alistipes;*
		19 ††	0.0817	*Bacteroidetes;Bacteroidia;Bacteroidales;Porphyromonadaceae;Tannerella;*
		292	0.0685	*Firmicutes;Clostridia;Clostridiales;Lachnospiraceae;Marvinbryantia;*
		26	0.0648	*Firmicutes;Clostridia;Clostridiales;Lachnospiraceae;Robinsoniella;*
		17	0.0608	*Bacteroidetes;Bacteroidia;Bacteroidales;Marinilabiaceae;Anaerophaga;*
		4 †	0.0598	*Bacteroidetes;Sphingobacteria;Sphingobacteriales;Cytophagaceae;Meniscus;*
		132	0.0595	*Proteobacteria;Betaproteobacteria;Burkholderiales;Oxalobacteraceae;Herbaspirillum;*

	69	0.0561	*Bacteroidetes;Bacteroidia;Bacteroidales;Rikenellaceae;Rikenella;*
	1	0.0523	*Bacteroidetes;Bacteroidia;Bacteroidales;Porphyromonadaceae;Paludibacter;*
TP3	1	0.1194	*Bacteroidetes;Bacteroidia;Bacteroidales;Porphyromonadaceae;Paludibacter;*
	25 †	0.1099	*Bacteroidetes;Bacteroidia;Bacteroidales;Porphyromonadaceae;Paludibacter;*
	76	0.1068	*Bacteroidetes;Bacteroidia;Bacteroidales;Porphyromonadaceae;Paludibacter;*
	99	0.0878	*Proteobacteria;Gammaproteobacteria;Chromatiales;Ectothiorhodospiraceae;Alkalispirillum;*
	2 ††	0.0851	*Firmicutes;Clostridia;Clostridiales;Lachnospiraceae;Robinsoniella;*
	132	0.0834	*Proteobacteria;Betaproteobacteria;Burkholderiales;Oxalobacteraceae;Herbaspirillum;*
	29 ††	0.0815	*Bacteroidetes;Bacteroidia;Bacteroidales;Bacteroidaceae;Bacteroides;*
	89	0.0802	*Proteobacteria;Epsilonproteobacteria;Campylobacterales;Helicobacteraceae;Helicobacter;*
	36 ††	0.0799	*Bacteroidetes;Bacteroidia;Bacteroidales;Rikenellaceae;Alistipes;*
	120	0.0731	*Bacteroidetes;Bacteroidia;Bacteroidales;Porphyromonadaceae;Paludibacter;*
TP5	1	0.2303	*Bacteroidetes;Bacteroidia;Bacteroidales;Porphyromonadaceae;Paludibacter;*
	154	0.1433	*Bacteroidetes;Bacteroidia;Bacteroidales;Porphyromonadaceae;Barnesiella;*
	29 ††	0.1361	*Bacteroidetes;Bacteroidia;Bacteroidales;Bacteroidaceae;Bacteroides;*
	36 ††	0.1360	*Bacteroidetes;Bacteroidia;Bacteroidales;Rikenellaceae;Alistipes;*
	152 ††	0.1283	*Bacteroidetes;Bacteroidia;Bacteroidales;Marinilabiaceae;Anaerophaga;*
	19 ††	0.1242	*Bacteroidetes;Bacteroidia;Bacteroidales;Porphyromonadaceae;Tannerella;*
	258	0.1217	*Firmicutes;Bacilli;Lactobacillales;Lactobacillaceae;Lactobacillus;*
	92	0.1192	*Firmicutes;Clostridia;Clostridiales;Lachnospiraceae;Lachnobacterium;*
	13	0.1180	*Firmicutes;Clostridia;Clostridiales;Lachnospiraceae;Robinsoniella;*
	112	0.1176	*Firmicutes;Clostridia;Clostridiales;Lachnospiraceae;Robinsoniella;*
TP11	29 ††	0.2822	*Bacteroidetes;Bacteroidia;Bacteroidales;Bacteroidaceae;Bacteroides;*
	16	0.1998	*Bacteroidetes;Bacteroidia;Bacteroidales;Marinilabiaceae;Anaerophaga;*
	23	0.1958	*Bacteroidetes;Bacteroidia;Bacteroidales;Porphyromonadaceae;Barnesiella;*
	35	0.1934	*Firmicutes;Clostridia;Clostridiales;Lachnospiraceae;Robinsoniella;*
	5 †	0.1708	*Proteobacteria;Epsilonproteobacteria;Campylobacterales;Helicobacteraceae;Helicobacter;*
	38 †	0.1575	*Bacteroidetes;Bacteroidia;Bacteroidales;Prevotellaceae;Prevotella;*
	132	0.1461	*Proteobacteria;Betaproteobacteria;Burkholderiales;Oxalobacteraceae;Herbaspirillum;*
	2 ††	0.1453	*Firmicutes;Clostridia;Clostridiales;Lachnospiraceae;Robinsoniella;*
	3 ††	0.1435	*Firmicutes;Negativicutes;Selenomonadales;Veillonellaceae;Anaerospora;*
	36 ††	0.1098	*Bacteroidetes;Bacteroidia;Bacteroidales;Rikenellaceae;Alistipes;*

Table S13: Comparison of network disintegration (based on the number of subnetworks) between empirical and simulated random graphs via two-sided Kolmogorov-Smirnov test.

		Failure		Attack	
Random graph model	Time point	*D*-value	$P_{Bonferroni}$	*D*-value	$P_{Bonferroni}$
Degree Sequence (similar degree distribution)	TP1	0.07407	1.00000	0.62963	0.00018
	TP3	0.08000	1.00000	0.16000	1.00000
	TP5	0.04545	1.00000	0.13636	1.00000
	TP11	0.68421	0.00062	0.78947	0.00006
Barabási (power=1, similar number of vertices)	TP1	1.00000	1.50364×10^{-11}	1.00000	1.50364×10^{-11}
	TP3	0.76000	1.00566×10^{-6}	0.84000	1.74637×10^{-7}
	TP5	0.72727	0.00003	0.81818	3.21452×10^{-6}
	TP11	0.89474	1.59116×10^{-7}	1.00000	4.48224×10^{-8}
Barabási (power=2, similar number of vertices)	TP1	1.00000	1.50364×10^{-11}	1.00000	1.50364×10^{-11}
	TP3	0.76000	1.00566×10^{-6}	1.00000	1.11104×10^{-10}
	TP5	0.72727	0.00003	1.00000	2.23157×10^{-9}
	TP11	0.89474	1.59116×10^{-7}	1.00000	4.48224×10^{-8}
Barabási (power=4, similar number of vertices)	TP1	1.00000	1.50364×10^{-11}	1.00000	1.50364×10^{-11}
	TP3	0.76000	1.00566×10^{-6}	1.00000	1.11104×10^{-10}
	TP5	0.72727	0.00003	1.00000	2.23157×10^{-9}
	TP11	0.89474	1.59116×10^{-7}	1.00000	4.48224×10^{-8}
Erdös-Renyi (random) (similar number of vertices and edges)	TP1	1.00000	1.50364×10^{-11}	0.70370	0.00001
	TP3	0.52000	0.00768	0.40000	0.14652
	TP5	0.50000	0.02916	0.45455	0.08492
	TP11	0.31579	1.00000	0.42105	0.27554
Small World (preferential reattachment, k=0.6, similar number of vertices)	TP1	1.00000	1.50364×10^{-11}	0.70370	0.00001
	TP3	0.52000	0.00768	0.40000	0.14652
	TP5	0.50000	0.02916	0.45455	0.08492
	TP11	0.31579	1.00000	0.42105	0.27554
Small World (preferential reattachment, k=0.8, similar number of vertices)	TP1	1.00000	1.50364×10^{-11}	0.70370	0.00001
	TP3	0.52000	0.00768	0.40000	0.14652
	TP5	0.50000	0.02916	0.40909	0.20141
	TP11	0.31579	1.00000	0.42105	0.27554
Power-law (degree distribution, power=4, similar number of vertices)	TP1	0.96296	1.07064×10^{-10}	0.70370	0.00001
	TP3	0.44000	0.05935	0.28000	1.00000
	TP5	0.40909	0.19739	0.31818	0.86150
	TP11	0.26316	1.00000	0.47368	0.11262
Power-law (degree distribution, power=6, similar number of vertices)	TP1	1.00000	1.50364×10^{-11}	0.70370	0.00001
	TP3	0.48000	0.02246	0.36000	0.31329
	TP5	0.45455	0.08019	0.45455	0.08492
	TP11	0.26316	1.00000	0.42105	0.27554
Power-law (degree distribution, power=8, similar number of vertices)	TP1	1.00000	1.50364×10^{-11}	0.70370	0.00001
	TP3	0.52000	0.00768	0.40000	0.14652
	TP5	0.50000	0.02916	0.45455	0.08492
	TP11	0.26316	1.00000	0.42105	0.27554

Supplementary references:

Clauset, A., Newman, M.E.J., and Moore, C. (2004). Finding community structure in very large networks. *Physical Review E* 70**,** 6.

Goto, Y., Obata, T., Kunisawa, J., Sato, S., Ivanov, I.I., Lamichhane, A., Takeyama, N., Kamioka, M., Sakamoto, M., Matsuki, T., Setoyama, H., Imaoka, A., Uematsu, S., Akira, S., Domino, S.E., Kulig, P., Becher, B., Renauld, J.-C., Sasakawa, C., Umesaki, Y., Benno, Y., and Kiyono, H. (2014). Innate lymphoid cells regulate intestinal epithelial cell glycosylation. *Science* 345**,** 1254009.

Newman, M.E.J., and Girvan, M. (2004). Finding and evaluating community structure in networks. *Phys Rev E Stat Nonlin Soft Matter Phys* 69**,** 026113.
